# Clinical Improvements From Telemedicine Interventions for Managing Type 2 Diabetes Compared With Usual Care: Systematic Review, Meta-Analysis, and Meta-Regression

**DOI:** 10.2196/70429

**Published:** 2026-02-18

**Authors:** Shujie Jiang, Xianru Gao, Haiqing Diao, Yang Zhang, Guangyu Lu, Xiaoguang Liu, Yuping Li

**Affiliations:** 1 School of Nursing Faculty of Medicine Yangzhou University Yangzhou China; 2 Jining Public Health Medical Center Jining China; 3 School of Basic Medical Sciences & School of Public Health Faculty of Medicine Yangzhou University Yangzhou China; 4 Neuro-Intensive Care Unit, Department of Neurosurgery Northern Jiangsu People's Hospital Affiliated to Yangzhou University Yangzhou China

**Keywords:** telemedicine, type 2 diabetes mellitus, systematic review, meta-analysis, metaregression

## Abstract

**Background:**

Type 2 diabetes mellitus (T2DM) is a prevalent chronic metabolic disorder that poses substantial challenges to global health care systems and patient management. Telemedicine, defined as the use of information and communication technologies to enhance health care delivery, has emerged as a potential tool to improve access to care and facilitate the management of T2DM.

**Objective:**

This systematic review and meta-analysis aimed to evaluate the clinical effectiveness of various telemedicine interventions compared with usual care in glycemic control, and cardiovascular health in adults with T2DM.

**Methods:**

A comprehensive literature search was conducted across databases such as PubMed, Cochrane Library, and Web of Science for randomized controlled trials (RCTs) published up to August 23, 2024. Eligible RCTs compared telemedicine interventions with usual care in adults with T2DM. The primary outcome assessed was hemoglobin A_1c_ (HbA_1c_) levels, while the secondary outcomes included mean glucose, fasting blood glucose, BMI, weight, systolic blood pressure, diastolic blood pressure, high-density lipoprotein cholesterol, and low-density lipoprotein cholesterol. The quality of the included studies was examined via the Cochrane risk-of-bias tool. Data were extracted and analyzed using a random-effects model, and meta-regression was performed to explore potential moderators. The quality of the evidence was assessed via the Grading of Recommendations, Assessment, Development, and Evaluation approach.

**Results:**

A total of 58 RCTs, encompassing 13,942 participants, were included in the analysis. Our findings showed that telemedicine interventions significantly improved HbA_1c_ levels compared with usual care (mean difference [MD] –0.38, 95% CI –0.49 to –0.27; *Z*=6.94; *P*<.001), despite high heterogeneity (*I*²=96%). Significant effects were also found for fasting blood glucose (MD –11.29, 95% CI –17.65 to –4.93; *Z*=3.48; *P*<.001), weight (MD –1.33, 95% CI –2.23 to –0.44; *Z*=2.91; *P*=.004), BMI (MD –0.43, 95% CI –0.72 to –0.13; *Z*=2.84; *P*=.004), systolic blood pressure (MD –2.14, 95% CI –3.02 to –1.26; *Z*=4.76; *P*<.001), and diastolic blood pressure (MD –1.24, 95% CI –2.02 to –0.46; *Z*=1.10; *P*=.002). No significant between-group differences were found in high-density lipoprotein cholesterol and low-density lipoprotein cholesterol improvement. Subgroup analyses revealed that telemedicine delivered by physicians, dietitians, and researchers achieved the most significant reductions in HbA_1c_ levels. Short-term and long-term interventions showed significant HbA_1c_ improvements, while medium-term interventions did not achieve statistical significance. Meta-regression analysis did not identify any statistically significant moderators.

**Conclusions:**

This review highlights telemedicine’s superior effectiveness over usual care in improving HbA_1c_ levels in patients with T2DM, regardless of the type of intervention. Telemedicine led by physicians, dietitians, and researchers showed the greatest efficacy in managing blood glucose levels. Furthermore, telemedicine interventions show promise for monitoring weight and cardiovascular health in patients with T2DM.

**Trial Registration:**

PROSPERO CRD42024608130; https://www.crd.york.ac.uk/prospero/display_record.php?RecordID=608130

## Introduction

Diabetes is a chronic metabolic disorder with increasing prevalence worldwide, placing a significant burden on global health care systems [1]. Currently, it affects more than 537 million adults, a number projected to increase to 783.2 million by 2045 [2]. Type 2 diabetes mellitus (T2DM) accounts for 90% of all diabetes cases [3]. T2DM not only poses a significant burden on individuals and society but also leads to reduced life expectancy and impaired quality of life [3,4]. Poor glycemic control in T2DM increases the risk of complications such as retinopathy, nephropathy, neuropathy, and cardiovascular diseases, leading to disability and premature mortality [5,6].

The management of T2DM is challenging and requires personalized, lifelong care [7,8]. Key aspects of T2DM management include glycemic control, weight management, and cardiovascular health monitoring, all of which are critical for preventing and managing the condition [9-11]. Researchers have identified 5 essential components of diabetes care: nutrition, physical activity, glycemic control, medical care, and patient education [12]. As such, continuous management and regular follow-up are imperative [13]. Global digitalization offers innovative digital opportunities for intensive diabetes management [14]. Telemedicine, which has proven effective in managing chronic diseases [15], holds particular promise for improving health care access for underserved populations. First conceptualized in the 1970s, telemedicine refers to the “use of [information and communication technologies] to improve patient outcomes by increasing access to care and medical information” [16]. In diabetes management specifically, telemedicine interventions have shown promise in enhancing glycemic control outcomes [14,17]. However, a recent review indicated that mobile health (mHealth) tools within telemedicine have shown only modest effectiveness [18].

Existing reviews often focus on specific types of telemedicine tools [19-21], settings [22], or providers [23], which limit generalizability across broader diabetes care contexts. This highlights the need for a more comprehensive and inclusive systematic review that synthesizes findings across various contexts [20,22,24]. With the onset of the COVID-19 pandemic, outpatient services for patients with diabetes have been limited, creating an opportunity for health care providers to implement telemedicine for diabetes management. Consequently, a substantial number of recent studies may have been published, warranting an updated review. This study aims to evaluate the impact of various telemedicine interventions on clinical outcomes in patients with T2DM compared with usual care through a systematic review and meta-analysis, providing valuable insights for future clinical practice and research.

## Methods

This systematic review followed the PRISMA (Preferred Reporting Items for Systematic Reviews and Meta-Analyses; checklist provided in Multimedia Appendix 1) guidelines and was registered in the PROSPERO database (CRD 42024608130).

### Search Strategy

We searched the following databases from inception to August 2024 to identify eligible randomized controlled trials (RCTs): Embase, PubMed, Scopus, CINAHL, and Web of Science. The search strategy was optimized to capture studies in telemedicine, people with T2DM, and glycemic control. Telemedicine is a broad and evolving field encompassing various aspects of remote health care delivery. While our search strategies primarily focused on the MeSH (Medical Subject Headings) term “telemedicine,” it is important to acknowledge that this field includes a wide range of related terms, such as “telemonitoring,” “telehealth,” “mobile health,” “mHealth,” “eHealth,” “teleconsultation,” and “telemetry.” The full search strategy is shown in the Multimedia Appendix 2 [25-82].

### Eligibility Criteria

RCTs that compared telemedicine interventions with usual care in adults (aged 18 years and older) with T2DM were included. The primary outcomes include an assessment of effectiveness based on clinical indicators, such as changes in hemoglobin A_1c_ (HbA_1c_) levels, mean glucose, fasting blood glucose (FBG), BMI, weight, systolic blood pressure (SBP), diastolic blood pressure (DBP), high-density lipoprotein cholesterol (HDL-c), and low-density lipoprotein cholesterol (LDL-c).

Here, telemedicine is defined as a form of health care delivery using electronic information and telecommunications, telemedicine facilitates information exchange, education, counseling, monitoring, and management between health care professionals and patients [83,84]. The usual care refers to face-to-face care, standard care, or traditional care.

Studies were excluded if they (1) reported findings from pregnant patients, patients with type 1 diabetes mellitus, and patients with prediabetes or other comorbidities; (2) reported populations at high risk only for diabetes or prediabetes; (3) not reported primary outcomes (changes of HbA_1c_ levels); (4) were published as conference abstracts, case reports, reviews, posters, comments, letters, and research protocols; or (5) were published in languages other than English.

### Outcomes

The primary outcome measure was a change in HbA_1c_ levels, while the secondary outcomes measure included changes in mean glucose, FBG, BMI, weight, SBP, DBP, HDL-c, and LDL-c. Telemedicine interventions were categorized into four types based on the mode of delivery [85]: (1) synchronous, involving real-time communication (eg, video or telephone consultations); (2) asynchronous, involving “store-and-forward” technologies such as messaging or email; (3) hybrid, defined as interventions that combined 2 or more telemedicine modalities, for example, in the study by Yang et al [25], patients uploaded daily records and received feedback and reminders through an application (asynchronous), in addition to receiving monthly telephone consultations (synchronous), thus meeting the criteria for hybrid telemedicine; and (4) unspecified, referring to studies that did not clearly report the telemedicine modality used and could not be classified as synchronous, asynchronous, or hybrid telemedicine.

In addition, the telemedicine interventions were categorized into 9 distinct content types: monitoring, counseling, education, reminders, training, feedback, medication management, treatment, and supervision [86]. The duration of interventions was also classified into 3 categories based on their length: short-term interventions (lasting up to 3 months), midterm interventions (lasting between 3 and 6 months), and long-term interventions (lasting 6 months or more) [87].

### Study Selection and Data Extraction

Duplicate studies were identified and removed using EndNote. The remaining studies were screened independently by 2 authors (JSJ and GXR) in a sequential manner of title, abstract, and full-text screening. Conflicts were settled by consulting a third author (LGY). Our data extraction included publication details (title, author, and year), study characteristics (country, purpose, blinding and randomization method, and year of publication), participant demographics, intervention details, comparison details, and results (primary and secondary outcomes with their SDs, SEs of the mean, and 95% CIs).

### Quality Assessment

The quality of the included RCTs was evaluated using the Grading of Recommendations Assessment, Development, and Evaluation (GRADE) approach [88]. This method is widely used to assess the certainty of evidence and the strength of recommendations. It provides a structured framework for judging the quality of evidence in systematic reviews. The GRADE system categorizes the certainty of evidence into 4 levels: high, moderate, low, and very low (Table S1 in Section 4 in Multimedia Appendix 2). Using the Cochrane risk-of-bias tool for RCTs, we evaluated the risk of bias and classified each trial as having a low, high, or unclear risk of bias for each area. Six bias domains are included in the tool: selection (random sequence generation and allocation concealment), performance, detection, attritions, reporting, and other biases [89]. Each trial’s risk of bias was evaluated independently by 2 reviewers (JSJ and XRG). In the event of a disagreement, consensus decision-making was used to achieve the most agreeable decision to all.

### Data Synthesis and Analysis

All outcomes were reported as means and SDs. If SDs were not reported and could not be obtained from study authors, they were estimated using available information such as SEs, 95% CIs, or *P* values, in accordance with the Cochrane Handbook. HbA_1c_ values were presented as percentages in accordance with the National Glycohemoglobin Standardization Program [90]; therefore, the values from studies that reported HbA_1c_ in millimoles per mole were converted to HbA_1c_ %. Likewise, mean glucose, FBG, HDL-c, and LDL-c reported in millimoles per liter were converted to milligrams per deciliter.

All statistical analyses were performed in Review Manager (version 5.4.1; Cochrane Collaboration) and Stata 16 (Stata Statistical Software: Release 16, StataCorp 2019; StataCorp LLC). Reported medians, IQRs, ranges, and CIs were transformed to means and SDs by traditional methods [91,92]. An overall treatment effect was estimated with a meta-analysis of the pool of included studies based on the mean difference (MD). Heterogeneity was assessed statistically using *I*^2^ tests. The results were combined with a random-effects model (due to heterogeneity, ie, an *I*^2^ statistic >50%). Subgroup analyses were conducted based on the characteristics of the intervention, including the type of telemedicine, the telemedicine provider, and the duration of the intervention. Univariate a priori subgroup analyses based on meta-regression of the telemedicine characteristics were conducted in Stata and combined with post hoc analyses of the association of study and patient characteristics with the treatment effect of telemedicine. Publication bias was evaluated using visual inspection of the funnel plot and the Egger test, both performed in Stata.

### Ethical Considerations

This study is a systematic review and meta-analysis based on previously published RCTs. No new human participants were involved, and no new data were collected. Therefore, ethics approval and informed consent were not required. All data used in this review were extracted from publicly available articles, and no identifiable personal information was involved.

## Results

**Figure 1:**
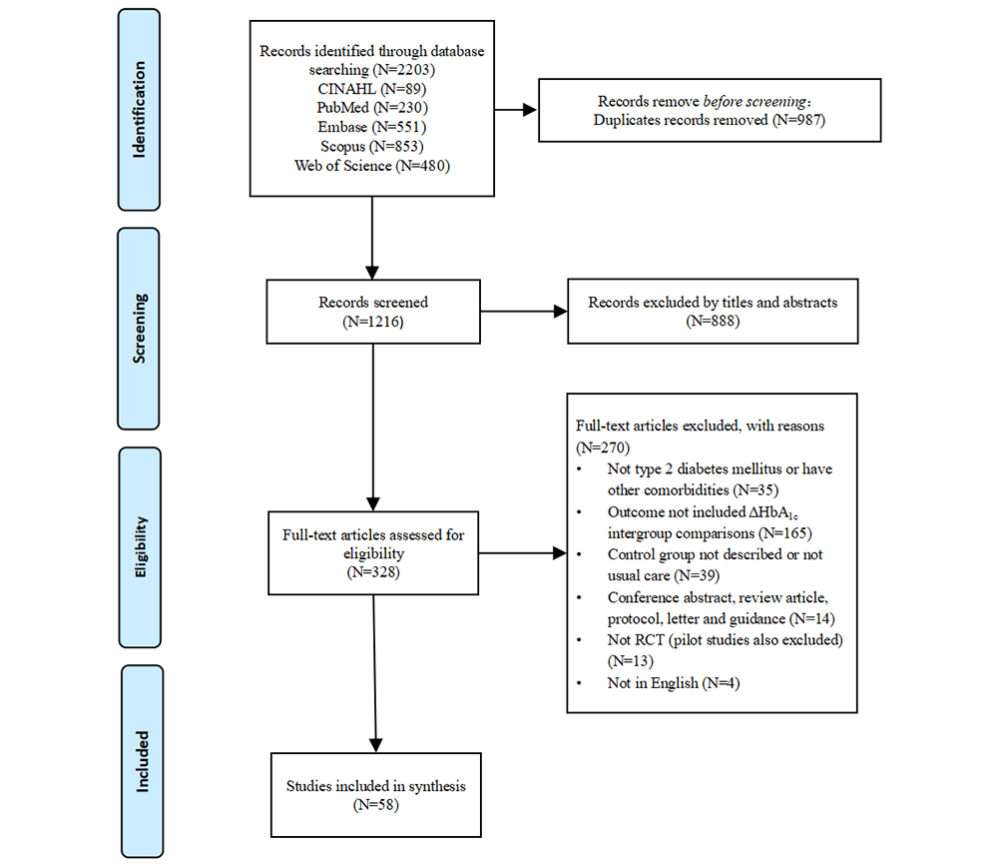
Flowchart summarizing the process of study selection. HbA_1c_: glycated hemoglobin; RCT: randomized controlled trial.

A total of 2203 studies were retrieved from 5 databases. After removing 987 duplicates, 1216 studies remained. Titles and abstracts were screened to determine eligibility, resulting in the exclusion of 888 studies. The full text of the remaining 328 studies was reviewed (Section 3 in Multimedia Appendix 2), and 58 studies were ultimately deemed eligible for analysis (Figure 1).

### Basic Characteristics of the Included Studies

A total of 58 RCTs, encompassing 13,942 participants, were included in the analysis. The detailed basic information, sample characteristics, type of telemedicine used, and clinical outcomes of the included studies are summarized in Table S2 in Section 4 in Multimedia Appendix 2.

The number of published studies has shown a steady increase over recent decades, with the most studies in the last 5 years accounting for 41% (24/58). Geographically, the studies were primarily conducted in Asia (18/58, 31%), Europe (18/58, 31%), and North America (17/58, 29%). Additional studies were conducted in Africa (1/58, 2%), South America (1/58, 2%), and Oceania (2/58, 3%; Figure S1 in Section 5 in Multimedia Appendix 2). Notably, 72% (42/58) of the studies originated from the high-income countries (Table 1). The most common settings for these studies were hospitals (20/58, 34%) and primary health care facilities (20/58, 34%).

**Table 1:** Characteristics of the randomized controlled trial studies (N=58).

Characteristics			Values
**Year of publication, n (%)**			
	2005-2009	3 (5)	
	2010-2014	11 (19)	
	2015-2019	20 (34)	
	2020-2024	24 (41)	
**Study location, n (%)**			
	Africa	2 (3)	
	Asia	18 (31)	
	Europe	18 (31)	
	North America	17 (29)	
	Oceania	2 (3)	
	South America	1 (2)	
**Whether the study site is in a** **high-income** **country, n (%)**			
	Yes	42 (72)	
	No	16 (28)	
**Study setting, n (%)**			
	Hospital	20 (34)	
	Primary care	20 (34)	
	Not reported	6 (10)	
	Others	12 (21)	
Total number of participants in included studies, n			13,942
**Mean age of participants, median (range)**			
	Intervention group	55.99 (33.00-73.05)	
	Control group	56.70 (32.40-73.04)	
**Proportion of male participants in percentage, median (range)**			
	Intervention group	51.7 (22.73-80.2)	
	Control group	53.00 (0-81.30)	
Trial length in months, median (range)			6 (3-24)

The median age of participants in the intervention group was 55.99 (range 33.00-73.05) years, while the control group’s median age was 56.70 (range 32.40-73.04) years. Male participants constituted a median of 51.7% in the intervention group and 53.0% in the control group (Table 2).

**Table 2:** Summary of descriptive characteristics of included papers.

Author (year)	Country	Setting	Sample size, I^a^/C^b^ (n)	Type of telemedicine	Intervention duration
Anzaldo-Campos et al (2016) [30]	Mexico	Family medical unit	102/100	Remote monitoring	10 months
Arora et al (2014) [31]	United States	Hospital	64/64	Asynchronous	6 months
Azelton et al (2021) [32]	United States	Family medical unit	16/14	Remote monitoring	12 weeks
Basudev et al (2016) [33]	United Kingdom	NR^c^	93/115	NR	12 months
Bentley et al (2016) [34]	United Kingdom	NR	9/9	Remote monitoring	12 weeks
Capozza et al (2015) [72]	United States	Primary care	58/35	Asynchronous	6 months
Cho et al (2017) [35]	South Korea	Hospital	244/240	Remote monitoring	24 weeks
Christensen et al (2022) [36]	Denmark	Hospital	81/55	Asynchronous	24 months
Christensen et al (2022) [37]	Denmark	Primary care	100/70	Synchronous and asynchronous	6 months
Dale et al (2009) [38]	United Kingdom	Primary care	44/97	Synchronous	6 months
Dario et al (2017) [39]	Italy	Local health authority	208/91	Remote monitoring	12 months
Dunkel et al (2023) [40]	Germany	NR	86/65	Synchronous and remote monitoring	12 months
Eakin et al (2014) [41]	Australia	Primary care	151/151	Synchronous	18 months
Farmer et al (2021) [42]	Southern Africa	Primary care	558/561	Asynchronous	12 months
Franc et al (2019) [73]	France	Hospital	C-G1: 62; I-G2: 64; I-G3: 63	Synchronous	13 months
Gerber et al (2023) [74]	United States	Primary care	109/112	Synchronous	12 months
Gong et al (2020) [43]	Australia	NR	93/94	Synchronous and remote monitoring	12 months
Greenwood et al (2015) [76]	United States	Primary care	45/45	Remote monitoring	6 months
Haghighinejad et al (2022) [26]	Iran	Hospital	I-G1: 50; I-G2: 50; C-G3: 46	Asynchronous	3 months
Hee-Sung (2007) [44]	South Korea	Hospital	12/15	Synchronous	12 weeks
Hoda et al (2023) [45]	India	Hospital	50/50	Synchronous and asynchronous	3 months
Holmen et al (2014) [27]	Norway	Hospital	I-G1: 50; I-G2: 50; C-G3: 50	Remote monitoring; synchronous and remote monitoring	12 months
Hsu et al (2016) [46]	United States	Hospital	20/20	Synchronous and asynchronous	12 ±2 weeks
Jantraporn et al (2019) [71]	Thailand	Primary care	26/27	Synchronous	12 weeks
Jarab et al (2012) [47]	Jordan	Hospital	85/86	Synchronous	6 months
Jeong et al (2018) [28]	South Korea	Hospital	I-G1: 113; I-G2: 112; C-G3: 113	Remote monitoring; synchronous and remote monitoring	24 weeks
Kempf et al (2017) [48]	Germany	Institute	93/74	Synchronous and remote monitoring	12 weeks
Kempf et al (2023) [49]	Germany	Institute	192/275	Synchronous and asynchronous	12 months
Khanna et al (2014) [77]	United States	Primary care	38/37	Synchronous	12 weeks
Kitazawa et al (2024) [50]	Japan	Institute	86/82	Remote monitoring	12 weeks
Kleinman et al (2016) [78]	India	NR	45/45	NR	6 months
Klingeman et al (2017) [79]	United States	Primary care	30/30	Synchronous and asynchronous	1 year
Kooiman et al (2018) [51]	United States	Health care organizations	40/32	Remote monitoring	12 weeks
Lauffenburger et al (2019) [52]	United States	Health insurer	700/700	Synchronous	12 months
Lee et al (2022) [29]	South Korea	Hospital	I-G1: 91; I-G2: 91; C-G3: 87	Remote monitoring; synchronous and remote monitoring	26 weeks
Lee et al (2020) [53]	Malaysia	Primary care	120/120	Remote monitoring	6 months
Leong et al (2022) [54]	China	Hospital	91/90	Synchronous and asynchronous	3 months
Lim et al (2021) [55]	Singapore	Primary care	99/105	Asynchronous and remote monitoring	6 months
Liou et al (2014) [56]	China	Primary care	54/41	Synchronous	6 months
Lorig et al (2008) [57]	Spain	Primary care	219/198	Synchronous	6 months
Luley et al (2011) [58]	Germany	Hospital	35/35	Remote monitoring	6 months
María Gómez et al (2022) [75]	Colombia	Hospital	41/45	Synchronous and asynchronous	3 months
Mitchell et al (2023) [59]	United States	Primary care	158/151	Synchronous	6 months
Orsama et al (2013) [60]	Finland	NR	24/24	Remote monitoring	10 months
Oseran et al (2022) [61]	United States	Primary care	130/130	Synchronous	6 months
Parsons et al (2019) [62]	United Kingdom	Hospital	148/151	Synchronous	12 months
Quinn et al (2011) [63]	United States	Primary care	62/56	Remote monitoring	12 months
Sachmechi et al (2023) [64]	United States	Hospital	39/39	Synchronous	12 weeks
Sarayani et al (2018) [65]	Iran	Hospital	50/50	Synchronous	3 months
Sun et al (2019) [66]	China	Hospital	44/47	Synchronous and remote monitoring	3 months
Tang et al (2013) [80]	United States	Institute	202/213	Asynchronous and remote monitoring	12 months
Torbjørnsen et al (2014) [67]	Norway	Study center	50/50	Synchronous and asynchronous	4 months
Turnin et al (2021) [81]	France	Hospital	128/135	Remote monitoring	1 year
Vaughan et al (2021) [82]	United States	Clinic	44/45	Synchronous	6 months
Yin et al (2022) [68]	China	Clinic	52/47	Remote monitoring	6 months
Zhang et al (2024) [69]	China	Primary care	1038/1034	Remote monitoring	24 months
Yang et al (2022) [25]	China	Primary care	50/50	Synchronous and asynchronous	12 months
Yang et al (2020) [70]	South Korea	Primary care	150/97	Asynchronous	3 months

^a^I: intervention group.

^b^C: control group.

^c^NR: not reported.

^d^G: group.

### Description of Telemedicine Interventions

Among the included studies, synchronous telemedicine, remote monitoring, and hybrid telemedicine were equally prevalent, with each accounting for 29% (18/63) of studies. Asynchronous telemedicine was used in 11% (7/63), and 3% (2/63) of studies did not specify the type of telemedicine used (Figure S3A in Section 5 in Multimedia Appendix 2). The most frequently implemented intervention components were monitoring (29 studies), counseling (29 studies), and education (24 studies; Figure S3B in Section 5 in Multimedia Appendix 2). Tools commonly used in telemedicine included telephone calls (42%), remote monitoring devices (39%), apps (36%), and text messages (32%; Figure S3C in Section 5 in Multimedia Appendix 2).

### Risk of Bias in the Included Studies

**Figure 2:**
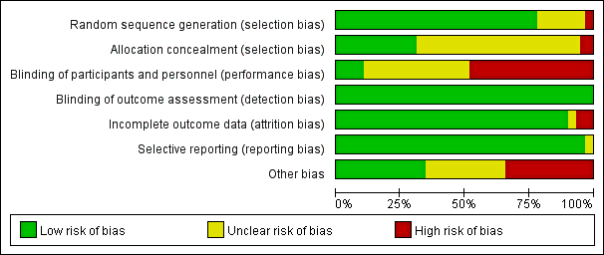
RoB assessment: summary plot. RoB: risk of bias.

The risk-of-bias assessment for the 58 included studies is summarized in Figure 2. All studies reported random sequence generation, with 47 (81%) studies assessed as low risk and 11 (19%) as unclear risk. For allocation concealment, 18 (31%) studies were judged as low risk, while 37 (64%) were unclear and 3 (5%) were rated as high risk. Blinding of participants and personnel was the most frequently identified source of bias. Only 6 (10%) studies were considered low risk, whereas 22 (38%) were unclear and 30 (51%) were at high risk. In contrast, all studies were rated as low risk for blinding of outcome assessment, as most primary outcomes were objective laboratory measures such as HbA_1c_. With respect to incomplete outcome data, 52 (90%) studies had a low risk of bias, 2 (3%) were unclear, and 4 (7%) were considered high risk due to missing data without adequate explanation. Selective reporting bias was generally low across the studies, with 56 (97%) studies judged to be low risk and 2 (3%) as unclear. For other sources of bias, 20 (34%) studies were considered low risk, 18 (31%) were unclear, and 20 (34%) were rated as high risk. Common issues included potential conflicts of interest, such as sponsorship from device companies or external funding sources. Overall, the methodological quality of the included studies was considered moderate. Details of the evaluation process are shown in Table S4 in Section 4 in Multimedia Appendix 2.

### Effects of Telemedicine on the Clinical Indicators of Patients With T2DM

Among the 58 included studies, 4 [26-29] used 2 types of telemedicine interventions, leading to a total of 62 intervention groups. Detailed intergroup comparisons for primary and secondary outcomes are shown in Table 3. Of the 58 studies, 47 provided sufficient quantitative data (eg, MDs with SDs, CIs, or SEs) to be included in the meta-analysis of HbA_1c_ outcomes. The remaining 11 studies, although included in the systematic review, did not report complete statistical information and were therefore not included in the quantitative synthesis for HbA_1c_. Overall, all trials demonstrated a positive impact of telemedicine on glycemic control (including changes in HbA_1c_, mean glucose, and FBG), weight management (change in weight and BMI), and cardiovascular health monitoring (including changes in SBP, DBP, LDL-c, and HDL-c).

**Table 3:** Effect of telemedicine on primary outcome (change of HbA_1c_^a^) and secondary outcome.

Clinical indicators outcome	HbA_1c_ (N=62)	Mean glucose (n=2)	FBG^b^ (N=15)	Weight (N=20)	BMI (N=20)	SBP^c^ (N=23)	DBP^d^ (N=22)	HDL-c^e^ (N=12)	LDL-c^f^ (N=14)
☆^g^	23	—^h^	6	6	7	4	2	—	1
+^i^	8	1	4	2	2	4	2	2	1
=^j^	31	1	5	11	11	14	18	9	12
×^k^	—	—	—	1	—	1	—	1	—

aHbA_1c_: hemoglobin A_1c_.

^b^FBG: fasting blood glucose.

^c^SBP: systolic blood pressure.

^d^DBP: diastolic blood pressure.

^e^HDL-c: high-density lipoprotein cholesterol.

^f^LDL-c: low-density lipoprotein cholesterol.

^g^Significant difference results in the intervention group compared with the control group; *P*<.01.

^h^Not available.

^i^Statistically significant results in the intervention group compared with the control group; *P*<.05.

^j^No significant difference results in the intervention group compared with the control group; *P*<.05.

^k^Statistically significant results in the control group compared with the intervention group.

### Meta-Analysis of the Effects of Telemedicine on Primary Outcome (△HbA_1c_)

#### Overall Meta-Analysis of △HbA_1c_ Between Intervention and Control Groups

**Figure 3:**
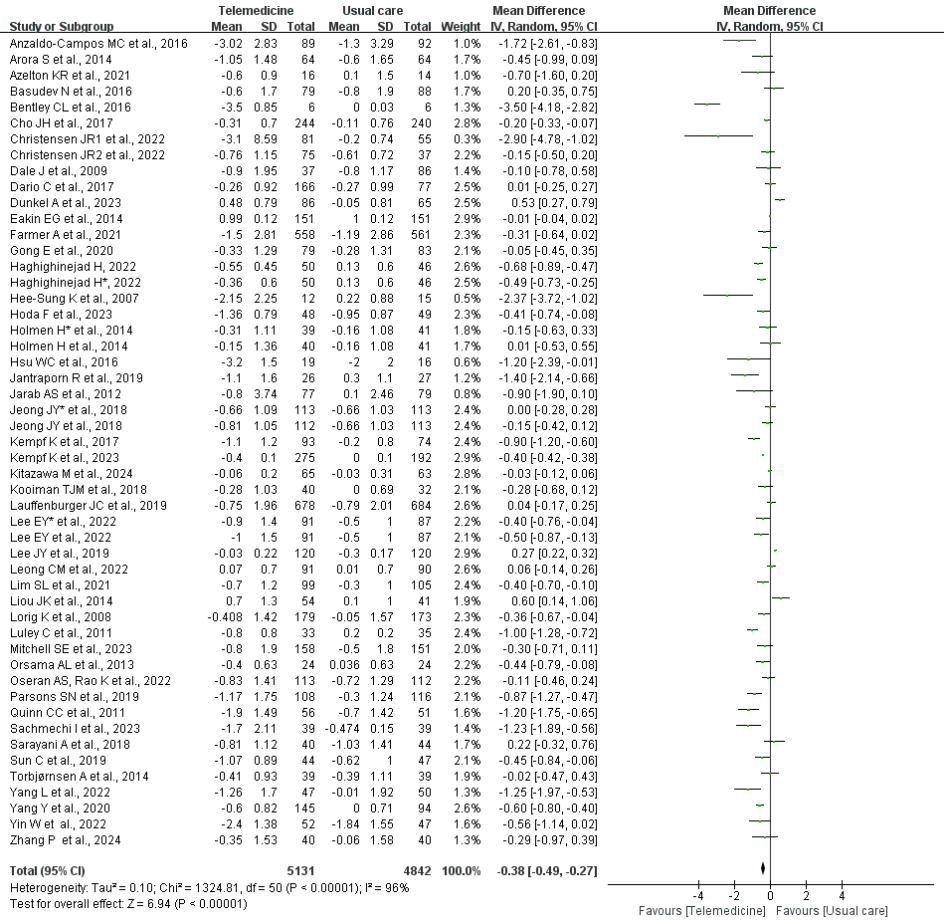
Forest plot of the overall meta-analysis of the change in HbA_1c_ between the telemedicine intervention group and the control group (47 studies [28,36-81]). *A study used 2 different telemedicine tools for the intervention, which are used here to differentiate. HbA_1c_: glycated hemoglobin.

A total of 47 studies [25-71] reported data on the change in HbA_1c_ levels between groups following telemedicine interventions, with 4 [26-29] studies including 2 types of telemedicine. The analysis revealed a high level of heterogeneity (*I*^2^=96%). Consequently, a random-effects model was used. The pooled results showed a significant reduction in HbA_1c_ levels in the telemedicine group compared with the control group (MD –0.38, 95% CI –0.49 to –0.27; *Z*=6.94; *P*<.001; Figure 3 [25-71]).

#### Subgroup Meta‑Analysis of △HbA_1c_ by Type of Telemedicine, Duration of Telemedicine Intervention, and Telemedicine Provider

**Figure 4:**
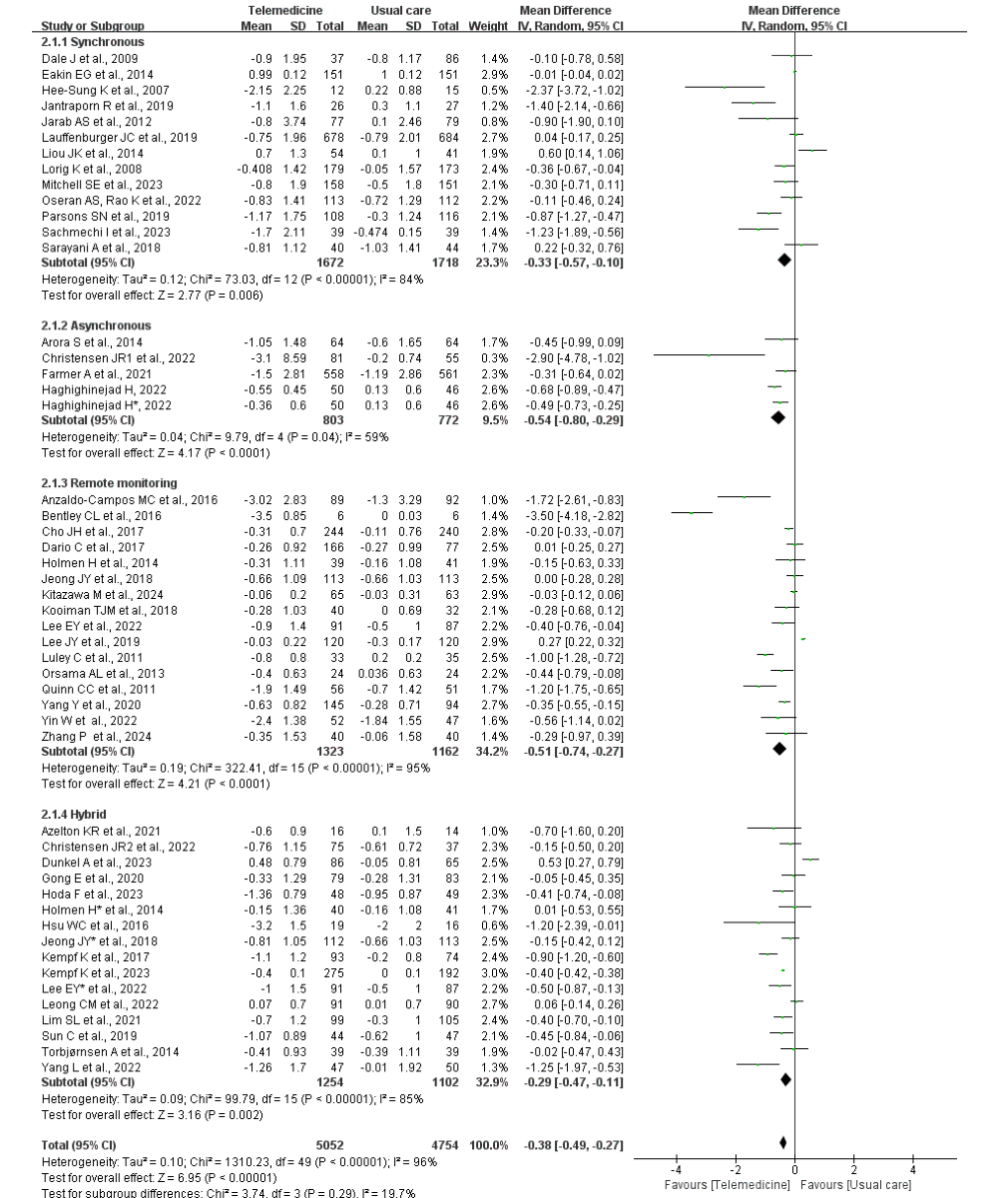
Forest plot of the subgroup meta-analysis of the change in HbA_1c_ by type of telemedicine intervention (46 studies [28,36-42,44-81]). *A study used 2 different telemedicine tools for the intervention, which are used here to differentiate. HbA_1c_: glycated hemoglobin.

For subgroups based on telemedicine type—synchronous, asynchronous, remote monitoring, and hybrid—telemedicine interventions consistently showed a greater reduction in HbA_1c_ compared with usual care. However, substantial heterogeneity was observed in all subgroups (Figure 4 [25-32,34-71]). The test for subgroup differences was not statistically significant (^2^_3_=3.74, *P*=.29; *I*^2^=19.7%).

**Figure 5:**
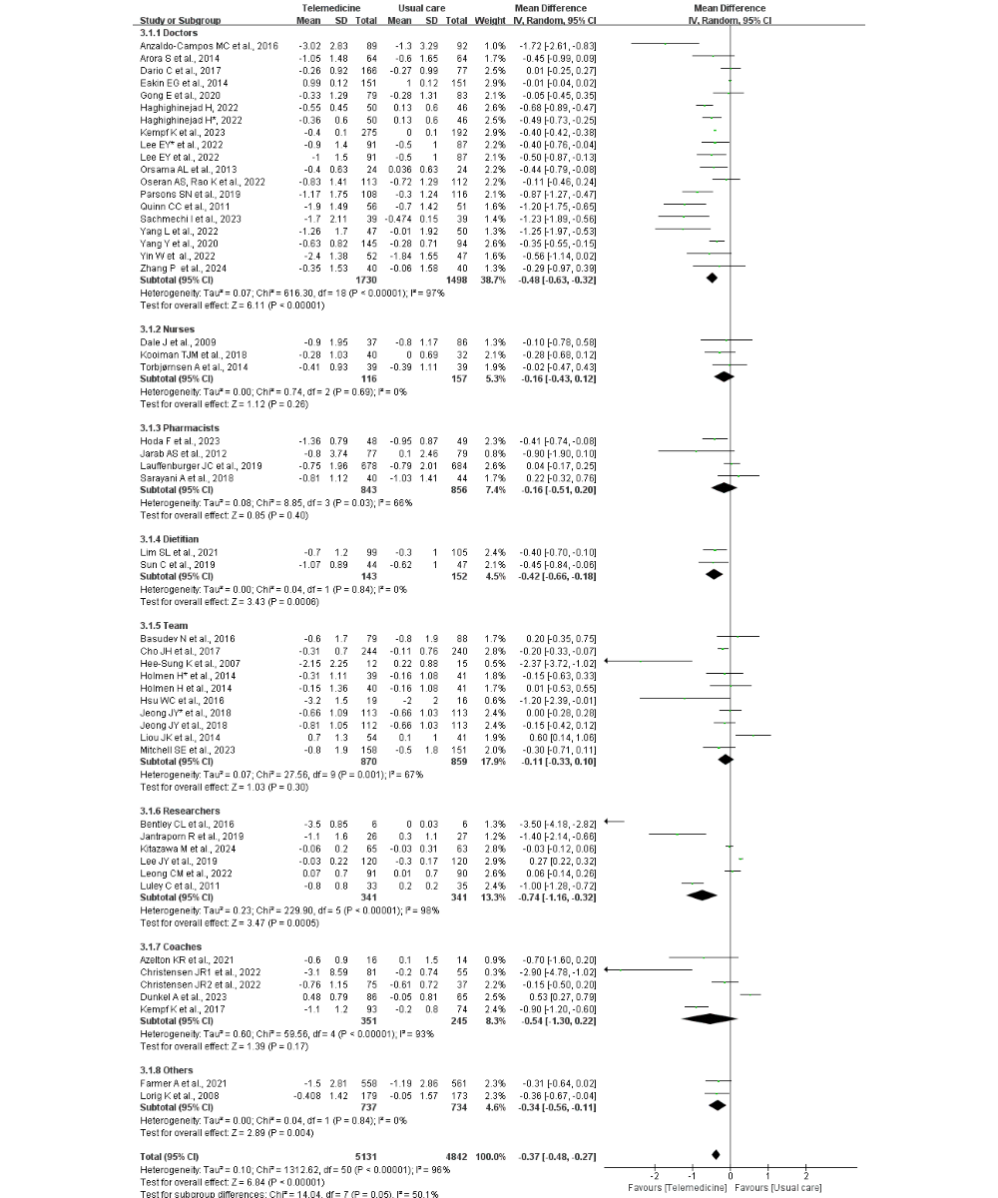
Forest plot of the subgroup meta-analysis of the change in HbA_1c_ by telemedicine provider (47 studies [28,36-81]). *A study used 2 different telemedicine tools for the intervention, which are used here to differentiate. HbA_1c_: glycated hemoglobin.

Eight subgroups were identified based on telemedicine providers (Figure 5 [25-71]). Telemedicine delivered by physicians, dietitians, and researchers showed a significantly greater reduction in HbA_1c_ levels compared with usual care but with very high heterogeneity in the physician and researcher subgroups. However, telemedicine delivered by nurses, pharmacists, medical teams, and coaches did not show statistically significant differences between the intervention and control groups. The between-subgroup difference approached statistical significance (^2^_7_=14.04, *P*=.05; *I*^2^=50.1%).

**Figure 6:**
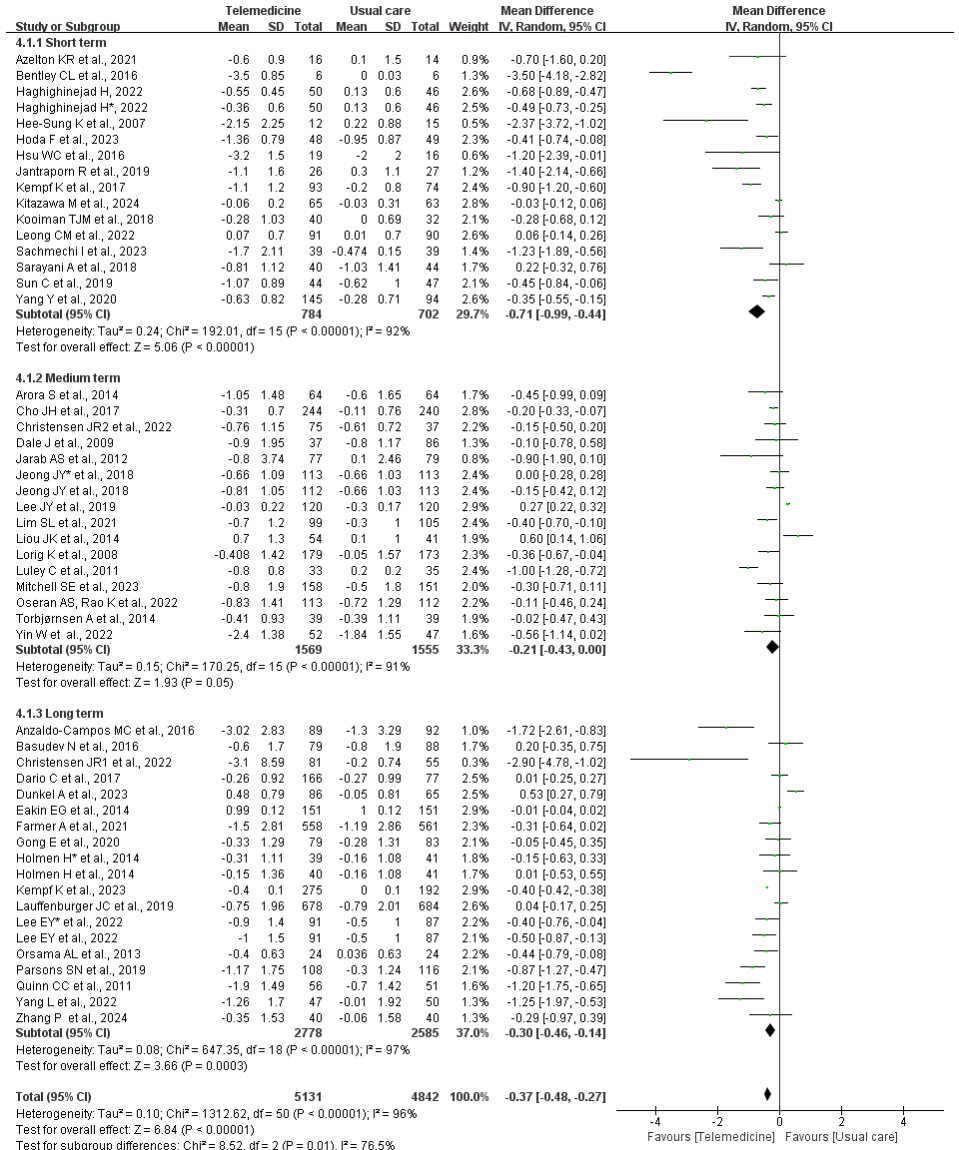
Forest plot of the subgroup meta-analysis of the change in HbA_1c_ by duration of telemedicine intervention (47 studies [28,36-81]). *A study used 2 different telemedicine tools for the intervention, which are used here to differentiate. HbA_1c_: glycated hemoglobin.

Subgroup analysis by intervention duration revealed significant reductions in HbA_1c_ levels for both short-term and long-term interventions. In contrast, medium-term interventions showed no statistically significant difference in HbA_1c_ changes between telemedicine and usual care (Figure 6 [25-71]). The test for subgroup differences indicated significant variation across durations (^2^_2_=8.52, *P*=.01; *I*^2^=76.5%).

### Meta-Analysis of the Effects of Telemedicine in Secondary Outcomes

#### Meta-Analysis of △FBG Between Intervention and Control Groups

A total of 13 studies [25,26,28,29,35,44,47,48,53,55,68-70] provided data on changes in FBG between groups, comprising 16 group comparisons (Figure S4A in Section 5 in Multimedia Appendix 2). A high level of heterogeneity was observed (*I*²=99%), prompting the use of a random-effects model. The analysis revealed a significant reduction in FBG levels in the intervention group (MD –11.29, 95% CI –17.65 to –4.93; *Z*=3.48; *P*<.001).

#### Meta-Analysis of △Weight Between Intervention and Control Groups

Nineteen studies [25,27-29,34-37,41,48-51,55,58,60,62,70] reported data related to weight change between groups (Figure S4B in Section 5 in Multimedia Appendix 2). The results showed high heterogeneity (*I*²=97%), and a random-effects model was applied. The pooled analysis indicated a significant reduction in weight in the intervention group (MD –1.33, 95% CI –2.23 to –0.44; *Z*=2.91; *P*=.004).

#### Meta-Analysis of △BMI Between Intervention and Control Groups

The BMI data (Figure S4C in Section 5 in Multimedia Appendix 2) were derived from 20 group comparisons across included studies [25,28-30,33,37,40,42,47-51,55,56,58,62,68,70], indicating high heterogeneity (*I*^2=^93%). Using a random-effects model, the intervention group exhibited a significant reduction in BMI (MD –0.43, 95% CI –0.72 to –0.13; *Z*=2.84; *P*=.004).

#### Meta-Analysis of △SBP Between Intervention and Control Groups

Data from 20 group comparisons on SBP changes [7,25,28,30,32,33,35,37,41,42,49,50,53,55,56,60,63,68-70] showed moderate heterogeneity (*I*^2=^64%; Figure S4D in Section 5 in Multimedia Appendix 2). Analyses showed more significant changes in SBP levels in the intervention group (MD –2.14, 95% CI –3.02 to –1.26; *Z*=4.76; *P*<.001).

#### Meta-Analysis of △DBP Between Intervention and Control Groups

Nineteen studies [25, 28, 30, 32, 33, 35, 37, 41, 47, 49, 50, 53, 55, 56, 60, 63, 68-70] provided data on DBP changes (Figure S4E in Section 5 in Multimedia Appendix 2). The results showed a moderate level of heterogeneity with an *I*^2^ value of 50%. Analyses showed more significant changes in DBP levels in the intervention group (MD –1.24, 95% CI –2.02 to –0.46; *Z*=1.10; *P*=.002).

#### Meta-Analysis of △LDL-c Between Intervention and Control Groups

A total of 8 studies [28-30,47,55,68-70] reported data related to LDL-c changes between groups (Figure S4F in Section 5 in Multimedia Appendix 2). The results showed a very high degree of heterogeneity (*I*^2^=100%). Analyses showed more significant changes in LDL-c levels in the intervention group (MD –0.69, 95% CI –11.69 to 10.31; *Z*=0.12; *P*=.90).

#### Meta-Analysis of △HDL-c Between Intervention and Control Groups

A total of 7 studies [28,30,47,55,68-70] reported data related to HDL-c changes between groups (Figure S4G in Section 5 in Multimedia Appendix 2). The results showed very high heterogeneity with an *I*^2^ value of 99%. The analysis showed more significant changes in HDL-c levels in the control group (MD –3.41, 95% CI –2.67 to 9.49; *Z*=1.10; *P*=.27).

### Meta-Rregression Analysis of Telemedicine

In this meta-regression analysis, none of the variables, including study location, whether the study was conducted in a high-income country, study setting, type of telemedicine, provider, and duration of intervention, significantly influenced the effect of telemedicine on HbA_1c_ levels (*P*>.05). Although the provider variable approached statistical significance (*P*=.084), no clear associations were found overall (Table 4).

**Table 4:** Association between study covariates and effect of telemedicine on HbA_1c_^a^% (meta-regression).

Variable	Coefficient	SE	Two-tailed *t* test (*df*)	*P* value > |t|	95% CI
Study location (continent)	0.031	0.141	0.220 (44)	.826	–0.254 to 0.317
Whether the study site is in a high-income country	0.112	0.275	0.410 (44)	.687	–0.442 to 0.666
Study setting	–0.072	0.101	–0.710 (44)	.480	–0.274 to 0.131
Type of telemedicine	–0.124	0.116	–1.070 (44)	.291	–0.357 to 0.110
Provider of telemedicine	0.084	0.048	1.770 (44)	.084	–0.012 to 0.180
Duration of telemedicine	0.142	0.134	1.070 (44)	.293	–0.127 to 0.412
Intercept	–0.815	0.836	–0.970 (44)	.335	–2.500 to 0.871

^a^HbA_1c_: hemoglobin A_1c_.

### Publication Bias

**Figure 7:**
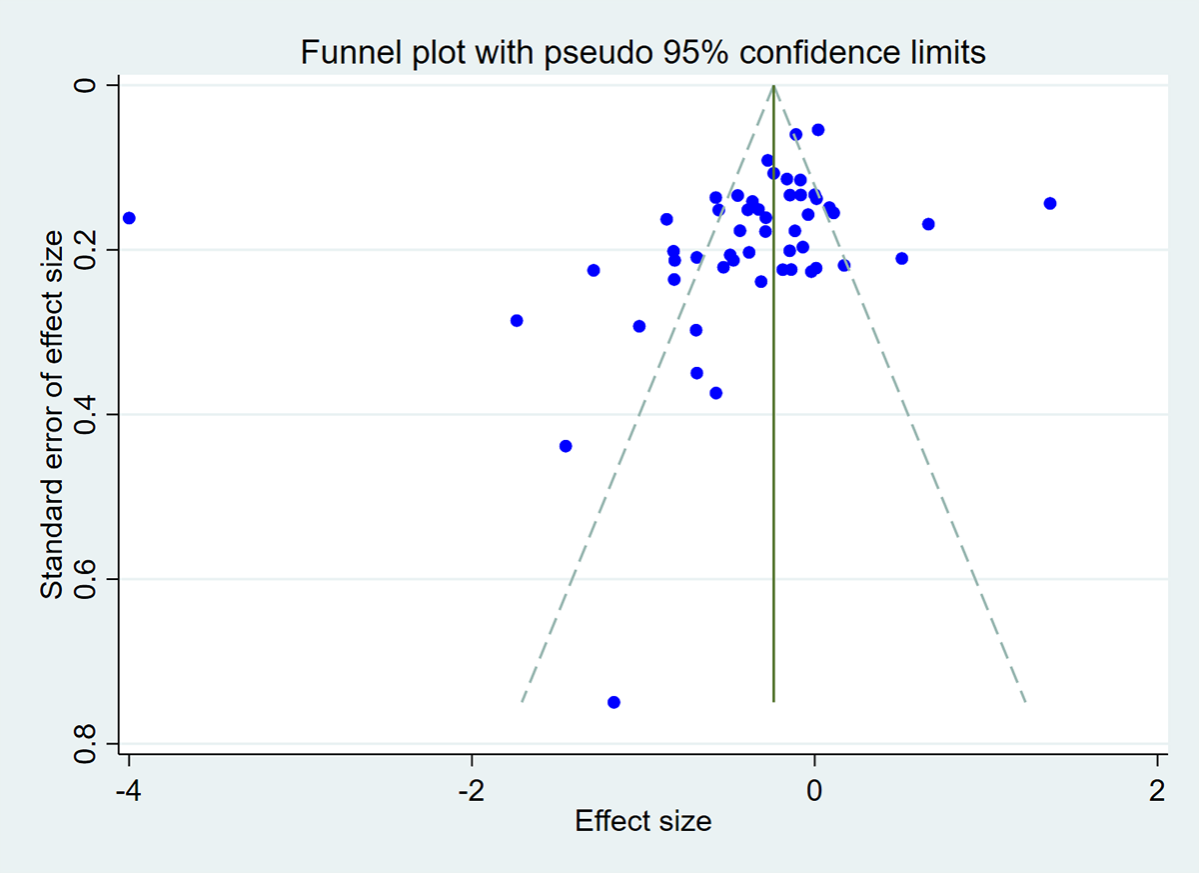
Funnel plot of effect size versus standard error for change in HbA_1c_.

Publication bias was assessed through both visual inspection of the funnel plot and formal statistical methods (Figure 7). Egger regression analysis showed borderline significance (*t*=–1.98; *P*=.054), suggesting potential asymmetry. To further assess this, we performed a trim-and-fill analysis using a random-effects model. When imputing missing studies on the left side, the method suggested 14 potentially missing studies, resulting in an adjusted effect size of –0.607 (95% CI –0.800 to –0.414), compared with the observed –0.379 (95% CI –0.579 to –0.180). In contrast, no studies were imputed on the right, and the effect estimate remained unchanged. This asymmetry indicates that publication bias may be present, possibly underestimating the true effect. Therefore, findings should be interpreted with caution.

### Sensitivity Analysis

In the primary meta-analysis of HbA_1c_ outcomes, significant heterogeneity (*I*²=96%) was observed. Despite this, sensitivity analysis indicated that the overall effect remained robust, as no single study had a disproportionate impact on the results (Figure S5 in Section 5 in Multimedia Appendix 2).

## Discussion

### Principal Findings

This systematic review and meta-analysis synthesized data from 58 RCTs to evaluate the effectiveness of telemedicine interventions in managing glycemic control, weight, and cardiovascular health in patients with T2DM. The results showed that telemedicine was more effective than usual care, especially in improving HbA_1c_ levels. All types of telemedicine—synchronous, asynchronous, remote monitoring, and hybrid—significantly reduced HbA_1c_ levels. Interventions delivered by physicians and dietitians had a greater impact on HbA_1c_ than those delivered by other providers, suggesting that provider expertise may affect outcomes. In addition, telemedicine demonstrated greater effectiveness in short- and long-term interventions, while mid-term interventions showed no statistically significant difference compared with usual care.

This review found that the use of telemedicine in patients with T2DM has been more extensively studied in high-income countries. Consistent with previous findings [87,93], the majority of the evidence (72%) originates from high-income countries, with the United States contributing the highest proportion (28%). Telemedicine remains in the early stages in low-income countries. Several factors may explain this trend, including the digital divide, legal barriers, and reimbursement challenges. The digital divide, characterized by differences in access to telecommunications technologies based on geographic and socioeconomic factors (eg, rural areas, low income, and low education levels), is a major barrier to the adoption of telemedicine [94]. In addition, legal barriers such as national licensing, practice laws, certification requirements, and liability issues also limit the use of telemedicine [94]. Legal and policy changes regarding reimbursement can ensure that the convenience and benefits of telemedicine are equitably accessible and enjoyed by the public, especially vulnerable groups. Limited reimbursement policies constrain the adoption of telemedicine, especially for underserved populations. Legal and policy reforms that ensure equitable reimbursement can enhance the accessibility and benefits of telemedicine for vulnerable groups [94,95]. National and local-level advocacy is needed to support legislation that can address reimbursement challenges and improve access to telemedicine.

The included studies exhibited substantial heterogeneity (*I*²=94%), a common finding in telemedicine reviews, where heterogeneity typically ranges from 65% to 99% [17,22,86,93]. Hence, this level of large heterogeneity found in the present review is not unexpected when compared with similar reviews. Several factors may account for the observed heterogeneity. First, differences in intervention characteristics—including provider type, modality (eg, synchronous, asynchronous, and remote monitoring), and intensity—likely contributed to variability in outcomes. Previous evidence has suggested that moderate- and high-intensity interventions tend to be more effective than low-intensity ones [96]. Second, differences in patient characteristics, such as comorbidities, digital health literacy, or engagement levels, may have influenced the effectiveness of interventions [97]. Only a few included studies explicitly assessed digital literacy, with most only including basic criteria such as the ability to read or operate a device. Finally, the inclusion of studies conducted in the early 2000s may have added to the heterogeneity, as telemedicine technologies and approaches have evolved significantly over the past 2 decades [98].

Based on the pooled analysis of the included trials, telemedicine emerges as a promising approach for providing effective consultation, monitoring, and management for patients with T2DM. The finding demonstrated that telemedicine interventions positively affect glycemic management, consistent with previous studies [14,99]. HbA_1c_ is an important indicator of long-term blood glucose control, and lower HbA_1c_ levels are linked to reduced risks of diabetes-related complications. Evidence suggests that a 1% decrease in HbA_1c_ is associated with a 37% reduction in microvascular complications, a 21% reduction in diabetes-related mortality, and a 14% decrease in the risk of myocardial infarction [100]. In this meta-analysis, telemedicine interventions were associated with a greater reduction in HbA_1c_ compared with usual care (MD –0.38, 95% CI –0.49 to –0.27; *P*<.001). These findings indicate that telemedicine has the potential not only to match, but in some aspects exceed, the clinical benefits of usual care in the management of T2DM.

Telemedicine may also provide broader benefits for managing metabolic and cardiovascular risk factors in patients with T2DM. Compared with usual care, telemedicine interventions showed greater improvements in FBG, body weight, BMI, SBP, and DBP. Although slight improvements in LDL-c and HDL-c levels were observed in the intervention group, no significant differences were found. This finding is consistent with a previous meta-analysis conducted in primary care settings, which also reported no significant differences between telemedicine and usual care in lipid-related outcomes [22]. However, the absence of significant effects on lipid profiles does not necessarily imply a lack of cardiovascular benefit. One possible explanation is that most telemedicine interventions for T2DM primarily focused on glycemic control, with fewer targeting lipid management specifically. Cardiovascular disease remains the leading cause of death among patients with T2DM [101]. This highlights that, beyond glycemic control, managing cardiovascular risk factors is also essential in T2DM population [102]. This review suggests that telemedicine plays an important role in improving glycemic control and cardiovascular-related health indicators in patients with T2DM. They also underscore the need for more standardized, comprehensive intervention protocols and better integration of multidisciplinary teams to enhance the overall effectiveness of telemedicine in future research and practice [103].

This study observed that different intervention modalities—including synchronous, asynchronous, remote monitoring, and hybrid models—were significantly more effective in improving HbA_1c_ levels compared with usual care, aligning with findings from prior reviews [20,104,105]. However, some studies suggested that synchronous and asynchronous interventions did not consistently outperform usual care. For instance, an earlier review indicated that synchronous and asynchronous teleconsultations did not lead to statistically significant reductions in HbA_1c_ levels [106]. Furthermore, while synchronous teleconsultations showed more substantial HbA_1c_ improvements, no significant benefits were observed in BMI, blood pressure, or cardiovascular health [20,105]. These discrepancies may be attributed to advancements in telemedicine technology, the enhancement of platform functionality, and the increasing standardization of service content. Differences in study design, including intervention frequency, methodology standardization, and varying content, could also play a significant role in influencing the outcomes observed across studies. Further research is needed to better understand these variations and optimize telemedicine interventions for more consistent and comprehensive clinical benefits.

The effectiveness of telemedicine interventions may vary depending on the type of health care provider involved. This review found that interventions led by doctors, dietitians, and researchers were significantly effective in improving HbA_1c_ levels compared with usual care. However, no significant differences were observed in the subgroups involving nurses, pharmacists, and coaches when compared with usual care. This discrepancy may be attributed to the limited ability of these providers to adjust treatment and management based on patients’ evolving conditions, highlighting the critical role of doctor involvement, guidance, and supervision in telemedicine interventions. Moreover, remote monitoring requires more complex operations, relying on the accuracy of devices and patients’ skills [107]. A previous study emphasized the importance of pretraining both patients and health care providers to enhance the reliability of remote monitoring [108]. Therefore, health care professionals need appropriate training, technical support, and clear guidelines to seamlessly integrate telemedicine into existing electronic health systems.

Although meta-regression analysis did not identify any statistically significant moderators of effect size, some variables showed trends that may have practical implications and warrant further investigation. For example, interventions delivered by physicians, dietitians, or researchers showed a trend toward greater improvements in HbA_1c_ (coefficient=0.084; *P*=.084), suggesting that provider expertise may influence outcomes. This is consistent with findings from subgroup analyses. Similarly, longer intervention durations (coefficient=0.142; *P*=.293) also demonstrated a nonsignificant positive trend, aligning with the notion that sustained engagement may enhance the effectiveness of disease management. A previous review has suggested that low-intensity telemedicine interventions are less effective than moderate- or high-intensity ones in supporting self-management among patients with type 2 diabetes [96]. The lack of statistically significant associations may be due to the complex nature of telemedicine interventions, residual confounding, and the predominantly categorical format of moderator variables, which may limit the sensitivity of meta-regression models to detect subtle effects. These observations highlight the importance of designing tailored telemedicine strategies that consider provider qualifications, intervention duration, and delivery context—that is, the health care setting, technological infrastructure, and patient engagement environment where telemedicine is implemented [103].

### Implications for Telemedicine Use on Future Public Health Emergencies

Beyond routine disease management, telemedicine also holds substantial potential in maintaining continuity of care during public health emergencies. It has already been used to manage disruptions in medical services caused by disasters such as the wildfires in Australia and Hurricane Harvey [109,110]. While the exact timing of natural disasters or infectious disease pandemics may be unpredictable, disruptions to medical services are certain to recur in the future [108]. This review focused on comparing telemedicine with usual care and demonstrated that telemedicine interventions achieved superior outcomes in patients with T2DM. These findings highlight the potential of telemedicine not only as a complementary strategy but also as an effective alternative to conventional care in situations where usual services are disrupted. To optimize the implementation of telemedicine in clinical practice, careful coordination with in-person health care systems is essential. This includes developing structured workflows involving health care professionals (eg, physicians, pharmacists, and dietitians) and support services (eg, scheduling departments, laboratories, and pharmacies). Patient stratification based on clinical needs is also crucial, as certain individuals—such as newly diagnosed patients or those starting therapies requiring specific training (eg, insulin injections)—may benefit more from hybrid care models that combine virtual assessment with in-person visits. Finally, establishing a robust, well-integrated telemedicine management system is critical to ensuring the continuous delivery of high-quality care for individuals with chronic conditions such as diabetes, particularly during emergencies.

### Strengths and Limitations

This meta-analysis has several advantages. First, to our knowledge, it is the first systematic review and meta-analysis of the management of T2DM that does not restrict the types of telemedicine interventions, tools, providers, or settings, and includes a wide range of important clinical outcomes. Second, we performed several important subgroup analyses based on the type of telemedicine, intervention content, and providers. Third, our study includes a sufficient number of long-term studies. In addition, we did not impose a time limit on published studies to ensure that earlier studies were not overlooked.

However, several limitations must be considered. First, a high level of heterogeneity was observed in the results, likely due to variations in the content of telemedicine interventions, types of media used, providers, and intervention durations. While heterogeneity is common in large meta-analyses, it remains a factor that could impact interpretation [111]. Second, due to the nature of telemedicine, blinding was not possible, which may have influenced the quality of the included studies and introduced potential bias. Third, in our subgroup analyses, the number of studies was limited and displayed high heterogeneity, so the findings should be interpreted cautiously. Further research is needed to strengthen the reliability of these results. Finally, the exclusion of non-English studies could be a potential source of publication bias.

### Conclusions

The results of this review demonstrate that telemedicine interventions, regardless of the type, are more effective than usual care in improving HbA_1c_ levels in patients with T2DM. In addition, telemedicine interventions led by physicians, dietitians, and researchers showed greater effectiveness in managing blood glucose levels. While the evidence for the impact of telemedicine on FBG, mean glucose, BMI, weight, blood pressure, LDL-c, and HDL-c is not as robust as for HbA_1c_, some studies have already highlighted the potential benefits of telemedicine for these outcomes.

## Data Availability

All data generated or analyzed during this study are included in this published article and its Multimedia Appendices.

## Appendix

Multimedia Appendix 1 PRISMA checklist.

Multimedia Appendix 2 Detailed list of included and excluded studies and supplementary figures and tables related to the meta-analysis.

## Abbreviations

DBP: diastolic blood pressure
FBG: fasting blood glucose
GRADE: Grading of Recommendations Assessment, Development, and Evaluation
HbA_1c_: hemoglobin A_1c_
HDL-c: high-density lipoprotein cholesterol
LDL-c: low-density lipoprotein cholesterol
MD: mean difference
MeSH: Medical Subject Headings
mHealth: mobile health
PRISMA: Preferred Reporting Items for Systematic Review and Meta-Analyses
RCT: randomized controlled trial
SBP: systolic blood pressure
T2DM: type 2 diabetes mellitus
