# Supplementary material for: Clinical Improvements From Telemedicine Interventions for Managing Type 2 Diabetes Compared With Usual Care: Systematic Review, Meta-Analysis, and Meta-Regression
Source: JMIR Mhealth Uhealth. 2026 Feb 18;14:e70429. doi: 10.2196/70429 (PMC12961393; doi:10.2196/70429)
Supplement: Multimedia Appendix 2 [file mhealth_v14i1e70429_app2.docx]

# Multimedia Appendix 2

# Section 1: Search strategy and databases

| **Database** | **Search terms** | **No. of studies generated** |
| --- | --- | --- |
| CINAHL | #1 SU Diabetes Mellitus,Type 2 OR TI Type 2 Diabetes OR TI Type 2 Diabetes Mellitus OR AB Type 2 Diabetes OR AB Type 2 Diabetes Mellitus  #2 SU telemedicine OR SU telemetry OR SU telemonitoring OR TI telemedicine OR TI telemetry OR TI telemonitoring OR TI virtual medicine OR TI mobile health OR TI mhealth OR TI telehealth OR TI ehealth OR TI telecare OR TI telemetries AB telemedicine OR AB telemetry OR AB telemonitoring OR AB virtual medicine OR AB mobile health OR AB mhealth OR AB telehealth OR AB ehealth OR AB telecare OR AB telemetries  #3 TI Randomized Controlled Trial OR AB Randomized Controlled Trial  #9 #1 AND #2 AND #3 | 89 |
| PubMed | #1 "diabetes mellitus, type 2"[MeSH Terms] OR "diabetes mellitus, type ii"[Title/Abstract] OR "type 2 diabetes mellitus"[Title/Abstract] OR "type 2 diabetes"[Title/Abstract] OR "diabetes, type 2"[Title/Abstract]  #2 "Telemedicine"[MeSH Terms] OR "virtual medicine"[Title/Abstract] OR "medicine, virtual"[Title/Abstract] OR "mobile health"[Title/Abstract] OR "health, mobile"[Title/Abstract] OR "mHealth"[Title/Abstract] OR "Telehealth"[Title/Abstract] OR "eHealth"[Title/Abstract] OR "Telecare"[Title/Abstract] OR "Telemetry"[MeSH Terms] OR "Telemetries"[Title/Abstract] OR "telemonitoring"[Title/Abstract]  #3 "Randomized Controlled Trial"[Publication Type] AND "randomized"[Title/Abstract]  #4 #1 AND #2 AND #3 | 230 |
| Scopus | #1 TITLE-ABS-KEY (Diabetes AND Mellitus AND Type 2) OR TITLE-ABS-KEY (Type 2 AND Diabetes AND Mellitus) TITLE-ABS-KEY (Type 2 AND Diabetes)  #2 TITLE-ABS-KEY (telemedicine) OR TITLE-ABS-KEY (mobile AND health) OR TITLE-ABS-KEY (mhealth) OR TITLE-ABS-KEY (telehealth) OR TITLE-ABS-KEY (ehealth) OR TITLE-ABS-KEY (virtual AND medicine) OR TITLE-ABS-KEY (telecare) OR TITLE-ABS-KEY (ehealth) OR TITLE-ABS-KEY (telemetry) OR TITLE-ABS-KEY (telemonitoring) OR TITLE-ABS-KEY (telemetries)  #3 TITLE-ABS-KEY (randomized controlled trial) | 853 |
| EMBASE | #1 'telemedicine'/exp  #2 'telemetry'/exp  #3 'telemonitoring'/exp  #4 'telemedicine':ti,ab,kw OR 'telemetry':ti,ab,kw OR 'telemonitoring':ti,ab,kw OR 'virtual medicine':ti,ab,kw OR 'mobile health':ti,ab,kw OR 'mhealth':ti,ab,kw OR 'telehealth':ti,ab,kw OR 'ehealth':ti,ab,kw OR 'telecare':ti,ab,kw OR 'telemetries':ti,ab,kw  #5 #1 OR #2 OR #3 OR #4  #6 'non insulin dependent diabetes mellitus'/exp  #7 'type 2 diabetes':ti,ab,kw OR 'non insulin dependent diabetes mellitus':ti,ab,kw  #8 #6 OR #7  #9 'randomized controlled trial'/exp  #10 #5 AND #8 AND #9 | 551 |
| Web of Science | #1 (TS=(telemedicine) OR AB=(telemedicine) OR AB=(health, mobile) OR AB=(mHealth) OR AB=(Telehealth) OR AB=(eHealth) OR AB=(virtual medicine) OR AB=(telecare) OR AB=(ehealth)) OR (TS=(telemetry) OR AB=(telemetry) OR AB=(telemetries)) OR(TS=(telemonitoring) OR AB=(telemonitoring))  #2 TS=(Diabetes Mellitus,Type 2) OR AB=(Diabetes Mellitus,Type 2) OR AB=(Type 2 Diabetes Mellitus) OR AB=(Type 2 Diabetes)  #3 TS=(randomized controlled trial) OR AB=(randomized controlled trial) OR TI =(randomized controlled trial)  #4 #1 AND #2 AND #3 | 480 |
| Total | - | 2,203 |

# Section 2: Articles included in full text [1-58]

[1] ANZALDO-CAMPOS M C, CONTRERAS S, VARGAS-OJEDA A, et al. Dulce wireless Tijuana: A randomized control trial evaluating the impact of project Dulce and short-term mobile technology on glycemic control in a family medicine clinic in Northern Mexico [J]. Diabetes Technol Ther, 2016, 18(4): 240-51.

[2] ARORA S, PETERS A L, BURNER E, et al. Trial to examine text message-based mHealth in emergency department patients with diabetes (TExT-MED): a randomized controlled trial [J]. Annals of emergency medicine, 2014, 63(6): 745-54.e6.

[3] AZELTON K R, CROWLEY A P, VENCE N, et al. Digital Health Coaching for Type 2 Diabetes: Randomized Controlled Trial of Healthy at Home [J]. Frontiers in Digital Health, 2021, 3.

[4] BASUDEV N, CROSBY-NWAOBI R, THOMAS S, et al. A prospective randomized controlled study of a virtual clinic integrating primary and specialist care for patients with Type 2 diabetes mellitus [J]. Diabetic medicine : a journal of the British Diabetic Association, 2016, 33(6): 768-76.

[5] BENTLEY C L, OTESILE O, BACIGALUPO R, et al. Feasibility study of portable technology for weight loss and HbA1c control in type 2 diabetes [J]. BMC medical informatics and decision making, 2016, 16(1).

[6] CAPOZZA K, WOOLSEY S, GEORGSSON M, et al. Going mobile with diabetes support: A randomized study of a text message-based personalized behavioral intervention for type 2 diabetes self-care [J]. Diabetes Spectr, 2015, 28(2): 83-91.

[7] CHO J H, KIM H S, YOO S H, et al. An Internet-based health gateway device for interactive communication and automatic data uploading: Clinical efficacy for type 2 diabetes in a multi-centre trial [J]. Journal of telemedicine and telecare, 2017, 23(6): 595-604.

[8] CHRISTENSEN J R, HESSELDAL L, OLESEN T B, et al. Long-term weight loss in a 24-month primary care-anchored telehealth lifestyle coaching program: Randomized controlled trial [J]. Journal of telemedicine and telecare, 2022, 28(10): 764-70.

[9] CHRISTENSEN J R, LAURSEN D H, LAURIDSEN J T, et al. Reversing Type 2 Diabetes in a Primary Care-Anchored eHealth Lifestyle Coaching Programme in Denmark: A Randomised Controlled Trial [J]. Nutrients, 2022, 14(16).

[10] DALE J, CARAMLAU I, STURT J, et al. Telephone peer-delivered intervention for diabetes motivation and support: The telecare exploratory RCT [J]. Patient Educ Couns, 2009, 75(1): 91-8.

[11] DARIO C, TOFFANIN R, CALCATERRA F, et al. Telemonitoring of Type 2 Diabetes Mellitus in Italy [J]. Telemedicine journal and e-health : the official journal of the American Telemedicine Association, 2017, 23(2): 143-52.

[12] DUNKEL A, VON STORCH K, HOCHHEIM M, et al. Long-term effects of a telemedically-assisted lifestyle intervention on glycemic control in patients with type 2 diabetes – A two-armed randomised controlled trial in Germany [J]. J Diabetes Metabolic Disord, 2023, 23(1): 519-32.

[13] EAKIN E G, WINKLER E A, DUNSTAN D W, et al. Living well with diabetes: 24-month outcomes from a randomized trial of telephone-delivered weight loss and physical activity intervention to improve glycemic control [J]. Diabetes care, 2014, 37(8): 2177-85.

[14] FARMER A, BOBROW K, LEON N, et al. Digital messaging to support control for type 2 diabetes (StAR2D): a multicentre randomised controlled trial [J]. BMC public health, 2021, 21(1).

[15] FRANC S, JOUBERT M, DAOUDI A, et al. Efficacy of two telemonitoring systems to improve glycaemic control during basal insulin initiation in patients with type 2 diabetes: The TeleDiab-2 randomized controlled trial [J]. Diabetes Obesity & Metabolism, 2019, 21(10): 2327-32.

[16] GERBER B, BIGGERS A, TILTON J J, et al. Mobile Health Intervention in Patients With Type 2 Diabetes A Randomized Clinical Trial [J]. JAMA network open, 2023, 6(9).

[17] GOMEZ A M, HENAO D C, MUNOZ O M, et al. Efficacy of the mHealth application in patients with type 2 diabetes transitioning from inpatient to outpatient care: A randomized controlled clinical trial [J]. Diabetes research and clinical practice, 2022, 189.

[18] GONG E, BAPTISTA S, RUSSELL A, et al. My diabetes coach, a mobile app⇓based interactive conversational agent to support type 2 diabetes self-management: randomized effectiveness-implementation trial [J]. Journal of medical Internet research, 2020, 22(11).

[19] GREENWOOD D A, BLOZIS S A, YOUNG H M, et al. Overcoming Clinical Inertia: A Randomized Clinical Trial of a Telehealth Remote Monitoring Intervention Using Paired Glucose Testing in Adults With Type 2 Diabetes [J]. Journal of medical Internet research, 2015, 17(7): e178.

[20] HAGHIGHINEJAD H, LIAGHAT L, MALEKPOUR F, et al. Comparing the effects of SMS-based education with group-based education and control group on diabetes management: a randomized educational program [J]. BMC Prim Care, 2022, 23(1).

[21] HEE-SUNG K. Impact of web-based nurse's education on glycosylated haemoglobin in type 2 diabetic patients [J]. J Clin Nurs, 2007, 16(7): 1361-6.

[22] HODA F, ARSHAD M, KHAN M A, et al. Impact of a mHealth Intervention in Type 2 Diabetes Mellitus Patients: A Randomized Clinical Trial [J]. SN Comprehensive Clinical Medicine, 2023, 5(1).

[23] HOLMEN H, TORBJORNSEN A, WAHL A K, et al. A Mobile Health Intervention for Self-Management and Lifestyle Change for Persons With Type 2 Diabetes, Part 2: One-Year Results From the Norwegian Randomized Controlled Trial RENEWING HEALTH [J]. JMIR mHealth and uHealth, 2014, 2(4).

[24] HSU W C, LAU K H, HUANG R, et al. Utilization of a Cloud-Based Diabetes Management Program for Insulin Initiation and Titration Enables Collaborative Decision Making Between Healthcare Providers and Patients [J]. Diabetes technology & therapeutics, 2016, 18(2): 59-67.

[25] JARAB A S, ALQUDAH S G, MUKATTASH T L, et al. Randomized controlled trial of clinical pharmacy management of patients with type 2 diabetes in an outpatient diabetes clinic in jordan [J]. Journal of Managed Care Pharmacy, 2012, 18(7): 516-26.

[26] JEONG J Y, JEON J H, BAE K H, et al. Smart Care Based on Telemonitoring and Telemedicine for Type 2 Diabetes Care: Multi-Center Randomized Controlled Trial [J]. Telemedicine journal and e-health : the official journal of the American Telemedicine Association, 2018, 24(8): 604-13.

[27] KEMPF K, ALTPETER B, BERGER J, et al. Efficacy of the Telemedical Lifestyle intervention Program TeLiPro in Advanced Stages of Type 2 Diabetes: A Randomized Controlled Trial [J]. Diabetes care, 2017, 40(7): 863-71.

[28] KEMPF K, DUBOIS C, ARNOLD M, et al. Effectiveness of the Telemedical Lifestyle Intervention Program TeLIPro for Improvement of HbA1c in Type 2 Diabetes: A Randomized-Controlled Trial in a Real-Life Setting [J]. Nutrients, 2023, 15(18).

[29] KHANNA R, STODDARD P J, GONZALES E N, et al. An automated telephone nutrition support system for Spanish-speaking patients with diabetes [J]. Journal of diabetes science and technology, 2014, 8(6): 1115-20.

[30] KITAZAWA M, TAKEDA Y, HATTA M, et al. Lifestyle Intervention With Smartphone App and isCGM for People at High Risk of Type 2 Diabetes: Randomized Trial [J]. J Clin Endocrinol Metab, 2024, 109(4): 1060-70.

[31] KLEINMAN N J, SHAH A, SHAH S, et al. Impact of the gather mHealth system on A1C: Primary results of a multisite randomized clinical trial among people with type 2 diabetes in India [J]. Diabetes care, 2016, 39(10): e169-e70.

[32] KLINGEMAN H, FUNNELL M, JHAND A, et al. Type 2 diabetes specialty clinic model for the accountable care organization era [J]. Journal of diabetes and its complications, 2017, 31(10): 1521-6.

[33] KOOIMAN T J M, DE GROOT M, HOOGENBERG K, et al. Self-tracking of Physical Activity in People With Type 2 Diabetes: A Randomized Controlled Trial [J]. Computers, informatics, nursing : CIN, 2018, 36(7): 340-9.

[34] LAUFFENBURGER J C, GHAZINOURI R, JAN S, et al. Impact of a novel pharmacist-delivered behavioral intervention for patients with poorly-controlled diabetes: The ENhancing outcomes through Goal Assessment and Generating Engagement in Diabetes Mellitus (ENGAGE-DM) pragmatic randomized trial [J]. PloS one, 2019, 14(4).

[35] LEE E Y, CHA S A, YUN J S, et al. Efficacy of Personalized Diabetes Self-care Using an Electronic Medical Record-Integrated Mobile App in Patients With Type 2 Diabetes: 6-Month Randomized Controlled Trial [J]. Journal of medical Internet research, 2022, 24(7): e37430.

[36] LEE J Y, CHAN C K Y, CHUA S S, et al. Telemonitoring and Team-Based Management of Glycemic Control on People with Type 2 Diabetes: a Cluster-Randomized Controlled Trial [J]. Journal of general internal medicine, 2020, 35(1): 87-94.

[37] LEONG C M, LEE T, CHIEN Y M, et al. Social Media-Delivered Patient Education to Enhance Self-management and Attitudes of Patients with Type 2 Diabetes During the COVID-19 Pandemic: Randomized Controlled Trial [J]. Journal of medical Internet research, 2022, 24(3).

[38] LIM S L, ONG K W, JOHAL J, et al. Effect of a Smartphone App on Weight Change and Metabolic Outcomes in Asian Adults with Type 2 Diabetes: A Randomized Clinical Trial [J]. JAMA network open, 2021, 4(6).

[39] LIOU J K, SOON M S, CHEN C H, et al. Shared care combined with telecare improves glycemic control of diabetic patients in a rural underserved community [J]. Telemedicine journal and e-health : the official journal of the American Telemedicine Association, 2014, 20(2): 175-8.

[40] LORIG K, RITTER P L, VILLA F, et al. Spanish diabetes self-management with and without automated telephone reinforcement: Two randomized trials [J]. Diabetes care, 2008, 31(3): 408-14.

[41] LULEY C, BLAIK A, RESCHKE K, et al. Weight loss in obese patients with type 2 diabetes: effects of telemonitoring plus a diet combination - the Active Body Control (ABC) Program [J]. Diabetes research and clinical practice, 2011, 91(3): 286-92.

[42] MITCHELL S E, BRAGG A, DE LA CRUZ B A, et al. Effectiveness of an Immersive Telemedicine Platform for Delivering Diabetes Medical Group Visits for African American, Black and Hispanic, or Latina Women With Uncontrolled Diabetes: The Women in Control 2.0 Noninferiority Randomized Clinical Trial [J]. Journal of medical Internet research, 2023, 25.

[43] ORSAMA A L, LäHTEENMäKI J, HARNO K, et al. Active assistance technology reduces glycosylated hemoglobin and weight in individuals with type 2 diabetes: Results of a theory-based randomized trial [J]. Diabetes Technol Ther, 2013, 16(SUPPL. 1): S62-S3.

[44] OSERAN A S, RAO K, CHANG Y, et al. HbA1c-Triggered Endocrinology Electronic Consultation for Type 2 Diabetes Management [J]. Journal of general internal medicine, 2022, 37(5): 1081-7.

[45] PARSONS S N, LUZIO S D, HARVEY J N, et al. Effect of structured self‐monitoring of blood glucose, with and without additional TeleCare support, on overall glycaemic control in non‐insulin treated Type 2 diabetes: the SMBG Study, a 12‐month randomized controlled trial [J]. Diabet Med, 2019, 36(5): 578-90.

[46] QUINN C C, SHARDELL M D, TERRIN M L, et al. Cluster-randomized trial of a mobile phone personalized behavioral intervention for blood glucose control [J]. Diabetes care, 2011, 34(9): 1934-42.

[47] SACHMECHI I, AMINI M, SALAM S, et al. Frequent Monitoring Of Blood Glucose Levels Via A Remote Patient Monitoring System Helps Improve Glycemic Control [J]. Journal of the Endocrine Society, 2023, 7: A519-A20.

[48] SARAYANI A, MASHAYEKHI M, NOSRATI M, et al. Efficacy of a telephone-based intervention among patients with type-2 diabetes; a randomized controlled trial in pharmacy practice [J]. International Journal of Clinical Pharmacy, 2018, 40(2): 345-53.

[49] SUN C, SUN L, XI S, et al. Mobile Phone-Based Telemedicine Practice in Older Chinese Patients with Type 2 Diabetes Mellitus: Randomized Controlled Trial [J]. JMIR mHealth and uHealth, 2019, 7(1): e10664.

[50] TANG P C, OVERHAGE J M, CHAN A S, et al. Online disease management of diabetes: Engaging and motivating patients online with enhanced resources-diabetes (EMPOWER-D), a randomized controlled trial [J]. J Am Med Informatics Assoc, 2013, 20(3): 526-34.

[51] TORBJORNSEN A, JENUM A K, SMåSTUEN M C, et al. A Low-Intensity Mobile Health Intervention With and Without Health Counseling for Persons With Type 2 Diabetes, Part 1: Baseline and Short-Term Results From a Randomized Controlled Trial in the Norwegian Part of RENEWING HEALTH [J]. JMIR mHealth and uHealth, 2014, 2(4).

[52] TURNIN M C, GOURDY P, MARTINI J, et al. Impact of a Remote Monitoring Programme Including Lifestyle Education Software in Type 2 Diabetes: Results of the Educ@dom Randomised Multicentre Study [J]. Diabetes Ther, 2021, 12(7): 2059-75.

[53] VAUGHAN E M, HYMAN D J, NAIK A D, et al. A Telehealth-supported, Integrated care with CHWs, and MEdication-access (TIME) Program for Diabetes Improves HbA1c: a Randomized Clinical Trial [J]. Journal of general internal medicine, 2021, 36(2): 455-63.

[54] YIN W, LIU Y, HU H, et al. Telemedicine management of type 2 diabetes mellitus in obese and overweight young and middle-aged patients during COVID-19 outbreak: A single-center, prospective, randomized control study [J]. PloS one, 2022, 17(9): e0275251.

[55] ZHANG P, TAO X, MA Y, et al. Improving the management of type 2 diabetes in China using a multifaceted digital health intervention in primary health care: the SMARTDiabetes cluster randomised controlled trial [J]. Lancet Reg Health West Pac, 2024, 49.

[56] YANG L, XU J, KANG C, et al. Effects of Mobile Phone Based Telemedicine Management in Patients With Type 2 Diabetes Mellitus: A Randomized Clinical Trial [J]. Am J Med Sci, 2022, 363(3): 224-31.

[57] YANG Y, LEE E Y, KIM H S, et al. Effect of a Mobile Phone-Based Glucose-Monitoring and Feedback System for Type 2 Diabetes Management in Multiple Primary Care Clinic Settings: Cluster Randomized Controlled Trial [J]. JMIR mHealth and uHealth, 2020, 8(2): e16266.

[58] JANTRAPORN R, PICHAYAPINYO P, LAGAMPAN S, et al. Effects of carbohydrate reduction program and telemonitoring on glycosylated hemoglobin in patients with poorly controlled type 2 diabetes: A randomized controlled trial [J]. J Med Assoc Thailand, 2019, 102(5): 523-9.

# Section 3: Exclude after reading the whole article

1. **Control group not described or not usual care [59-97]**

[59] BASTYR E J, ZHANG S, MOU J, et al. Performance of an Electronic Diary System for Intensive Insulin Management in Global Diabetes Clinical Trials [J]. Diabetes Technol Ther, 2015, 17(8): 571-9.

[60] BERGENSTAL R M, ANDERSON R L, BINA D M, et al. Impact of modem-transferred blood glucose data on clinician work efficiency and patient glycemic control [J]. Diabetes technology & therapeutics, 2005, 7(2): 241-7.

[61] BOLLYKY J B, BRAVATA D, YANG J, et al. Remote Lifestyle Coaching Plus a Connected Glucose Meter with Certified Diabetes Educator Support Improves Glucose and Weight Loss for People with Type 2 Diabetes [J]. Journal of diabetes research, 2018, 2018: 3961730.

[62] CHAO D Y P, LIN T M Y, MA W Y. Enhanced self-efficacy and behavioral changes among patients with diabetes: Cloud-based mobile health platform and mobile app service [J]. JMIR Diabetes, 2019, 4(2).

[63] CHO J H, LEE H C, LIM D J, et al. Mobile communication using a mobile phone with a glucometer for glucose control in Type 2 patients with diabetes: as effective as an Internet-based glucose monitoring system [J]. Journal of telemedicine and telecare, 2009, 15(2): 77-82.

[64] CROWLEY M J, TARKINGTON P E, BOSWORTH H B, et al. Effect of a Comprehensive Telehealth Intervention vs Telemonitoring and Care Coordination in Patients With Persistently Poor Type 2 Diabetes Control A Randomized Clinical Trial [J]. JAMA internal medicine, 2022, 182(9): 943-52.

[65] CROWLEY M J, TARKINGTON P E, BOSWORTH H B, et al. Comparing two telehealth interventions for patients with clinic-refractory type 2 diabetes: A randomized controlled trial [J]. Diabetes, 2021, 70(SUPPL 1).

[66] DUFFY R A, JEFFREYS A S, COFFMAN C J, et al. Evaluating Therapeutic Inertia in Two Telehealth Interventions for Type 2 Diabetes: Secondary Analyses of a Randomized Trial [J]. Telemedicine journal and e-health : the official journal of the American Telemedicine Association, 2024, 30(6): e1790-e7.

[67] ELLIS D A, NAAR-KING S, CHEN X, et al. Multisystemic therapy compared to telephone support for youth with poorly controlled diabetes: findings from a randomized controlled trial [J]. Annals of behavioral medicine : a publication of the Society of Behavioral Medicine, 2012, 44(2): 207-15.

[68] FENG Y H, ZHAO Y X, MAO L Q, et al. The Effectiveness of an eHealth Family-Based Intervention Program in Patients With Uncontrolled Type 2 Diabetes Mellitus (T2DM) in the Community Via WeChat: Randomized Controlled Trial [J]. JMIR mHealth and uHealth, 2023, 11(1).

[69] HEISLER M, CHOI H, MASE R, et al. Effectiveness of Technologically Enhanced Peer Support in Improving Glycemic Management Among Predominantly African American, Low-Income Adults With Diabetes [J]. The Diabetes educator, 2019, 45(3): 260-71.

[70] JOHNSON C M, MELKUS G D, REAGAN L, et al. Learning in a Virtual Environment to Improve Type 2 Diabetes Outcomes: Randomized Controlled Trial [J]. JMIR Form Res, 2023, 7.

[71] KOBE E A, LEWINSKI A, DANUS S, et al. Implementation of an intensive telehealth intervention for rural patients with uncontrolled diabetes [J]. Diabetes, 2020, 69.

[72] LARI H, NOROOZI A, TAHMASEBI R. Comparison of multimedia and SMS education on the physical activity of diabetic patients: An application of health promotion model [J]. Iran Red Crescent MedJ, 2018, 20(S1).

[73] LEVY N K, ORZECK-BYRNES N A, AIDASANI S R, et al. Transition of a text-based insulin titration program from a randomized controlled trial into real-world settings: Implementation study [J]. Journal of medical Internet research, 2018, 20(3).

[74] MAYBERRY L S, FELIX H C, HUDSON J, et al. Effectiveness-implementation trial comparing a family model of diabetes self-management education and support with a standard model [J]. Contemporary clinical trials, 2022, 121: 106921.

[75] MUNSHI M N, SEGAL A R, SUHL E, et al. Assessment of barriers to improve diabetes management in older adults [J]. Diabetes care, 2013, 36(3): 543-9.

[76] ST-JULES D E, HU L, WOOLF K, et al. An Evaluation of Alternative Technology- Supported Counseling Approaches to Promote Multiple Lifestyle Behavior Changes in Patients With Type 2 Diabetes and Chronic Kidney Disease [J]. J Renal Nutr, 2023, 33(1): 35-44.

[77] STONE R A, RAO R H, SEVICK M A, et al. Active Care Management Supported by Home Telemonitoring in Veterans With Type 2 Diabetes The DiaTel randomized controlled trial [J]. Diabetes care, 2010, 33(3): 478-84.

[78] TRIEF P M, FISHER L, SANDBERG J, et al. Health and Psychosocial Outcomes of a Telephonic Couples Behavior Change Intervention in Patients With Poorly Controlled Type 2 Diabetes: A Randomized Clinical Trial [J]. Diabetes care, 2016, 39(12): 2165-73.

[79] TU Y Z, CHANG Y T, CHIOU H Y, et al. The effects of continuous usage of a diabetes management app on glycemic control in real-world clinical practice: Retrospective analysis [J]. Journal of medical Internet research, 2021, 23(7).

[80] WOLF M S, SELIGMAN H, DAVIS T C, et al. Clinic-based versus outsourced implementation of a diabetes health literacy intervention [J]. Journal of general internal medicine, 2014, 29(1): 59-67.

[81] XU R, XING M, JAVAHERIAN K, et al. Improving HbA(1c) with Glucose Self-Monitoring in Diabetic Patients with EpxDiabetes, a Phone Call and Text Message-Based Telemedicine Platform: A Randomized Controlled Trial [J]. Telemedicine journal and e-health : the official journal of the American Telemedicine Association, 2020, 26(6): 784-93.

[82] YAN X, MATCHAR D B, SIVAPRAGASAM N, et al. Sequential Multiple Assignment Randomized Trial (SMART) to identify optimal sequences of telemedicine interventions for improving initiation of insulin therapy: A simulation study [J]. BMC medical research methodology, 2021, 21(1): 200.

[83] ZHOU W, CHEN M, YUAN J, et al. Welltang - A smart phone-based diabetes management application - Improves blood glucose control in Chinese people with diabetes [J]. Diabetes research and clinical practice, 2016, 116: 105-10.

[84] HöCHSMANN C, MüLLER O, AMBüHL M, et al. Novel Smartphone Game Improves Physical Activity Behavior in Type 2 Diabetes [J]. American journal of preventive medicine, 2019, 57(1): 41-50.

[85] HU X, DENG H, ZHANG Y, et al. Efficacy and safety of a decision support intervention for basal insulin self-titration assisted by the nurse in outpatients with T2DM: A randomized controlled trial [J]. Diabetes Metab Syndr Obes, 2021, 14: 1315-27.

[86] KIM E K, CHO Y M. The effect of a smartphone-based, patient-centered diabetes care system in patients with type 2 diabetes: A randomized, controlled trial for 24 weeks [J]. Diabetes care, 2019, 42(7): E126.

[87] KIM G, KIM S, LEE Y B, et al. A randomized controlled trial of an app-based intervention on physical activity and glycemic control in people with type 2 diabetes [J]. BMC Med, 2024, 22(1).

[88] KIRKLAND E B, MARSDEN J, ZHANG J, et al. Remote patient monitoring sustains reductions of hemoglobin A1c in underserved patients to 12 months [J]. Primary care diabetes, 2021, 15(3): 459-63.

[89] LEE M K, LEE D Y, AHN H Y, et al. A Novel User Utility Score for Diabetes Management Using Tailored Mobile Coaching: Secondary Analysis of a Randomized Controlled Trial [J]. JMIR mHealth and uHealth, 2021, 9(2): e17573.

[90] LI J, WEI D, LIU S, et al. Efficiency of an mHealth App and Chest-Wearable Remote Exercise Monitoring Intervention in Patients With Type 2 Diabetes: A Prospective, Multicenter Randomized Controlled Trial [J]. JMIR mHealth and uHealth, 2021, 9(2): e23338.

[91] MARKOWITZ J T, COUSINEAU T, FRANKO D L, et al. Text messaging intervention for teens and young adults with diabetes [J]. Journal of diabetes science and technology, 2014, 8(5): 1029-34.

[92] PRESLEY C, AGNE A, SHELTON T, et al. Mobile-Enhanced Peer Support for African Americans with Type 2 Diabetes: a Randomized Controlled Trial [J]. Journal of general internal medicine, 2020, 35(10): 2889-96.

[93] RODRíGUEZ-IDíGORAS M I, SEPúLVEDA-MUñOZ J, SáNCHEZ-GARRIDO-ESCUDERO R, et al. Telemedicine influence on the follow-up of type 2 diabetes patients [J]. Diabetes technology & therapeutics, 2009, 11(7): 431-7.

[94] SASLOW L R, MASON A E, KIM S, et al. An Online Intervention Comparing a Very Low-Carbohydrate Ketogenic Diet and Lifestyle Recommendations Versus a Plate Method Diet in Overweight Individuals With Type 2 Diabetes: A Randomized Controlled Trial [J]. Journal of medical Internet research, 2017, 19(2): e36.

[95] TIMURTAS E, INCEER M, MAYO N, et al. Technology-based and supervised exercise interventions for individuals with type 2 diabetes: Randomized controlled trial [J]. Primary care diabetes, 2022, 16(1): 49-56.

[96] WALKER E A, SHMUKLER C, ULLMAN R, et al. Results of a successful telephonic intervention to improve diabetes control in urban adults: A randomized trial [J]. Diabetes care, 2011, 34(1): 2-7.

[97] WAYNE N, F PEREZ D, M KAPLAN D, et al. Health Coaching Reduces HbA1c in Type 2 Diabetic Patients From a Lower-Socioeconomic Status Community: A Randomized Controlled Trial [J]. Journal of medical Internet research, 2015, 17(10): e224-19.

**2. Outcome not included ΔHbA1c intergroup comparisons [98-262]**

[98] AGARWAL P, MUKERJI G, DESVEAUX L, et al. Mobile app for improved self-management of type 2 diabetes: Multicenter pragmatic randomized controlled trial [J]. JMIR mHealth and uHealth, 2019, 7(1).

[99] BANU B, KO K C, KHAN M M H, et al. Effects of traditional versus m-Health educational interventions for diabetic patients: a randomised controlled trial in peripheral district of Bangladesh [J]. Diabetes Epidemiology and Management, 2023, 9.

[100] BOHINGAMU MUDIYANSELAGE S, STEVENS J, WATTS J J, et al. Personalised telehealth intervention for chronic disease management: A pilot randomised controlled trial [J]. Journal of telemedicine and telecare, 2019, 25(6): 343-52.

[101] FANG R H, DENG X X. Electronic messaging intervention for management of cardiovascular risk factors in type 2 diabetes mellitus: A randomised controlled trial [J]. J Clin Nurs, 2018, 27(3-4): 612-20.

[102] GITHINJI P, DAWSON J A, APPIAH D, et al. A Culturally Sensitive and Theory-Based Intervention on Prevention and Management of Diabetes: A Cluster Randomized Control Trial [J]. Nutrients, 2022, 14(23).

[103] GRILO S A, SHALLCROSS A J, OGEDEGBE G, et al. Food Insecurity and Effectiveness of Behavioral Interventions to Reduce Blood Pressure, New York City, 2012-2013 [J]. Preventing Chronic Disease, 2015, 12.

[104] HANNON T S, YAZEL-SMITH L G, HATTON A S, et al. Advancing diabetes management in adolescents: Comparative effectiveness of mobile self-monitoring blood glucose technology and family-centered goal setting [J]. Pediatr Diabetes, 2018, 19(4): 776-81.

[105] HANSEN C R, PERRILD H, KOEFOED B G, et al. Video consultations as add-on to standard care among patients with type 2 diabetes not responding to standard regimens: a randomized controlled trial [J]. European journal of endocrinology, 2017, 176(6): 727-36.

[106] HEITKEMPER E M, MAMYKINA L, TOBIN J N, et al. Baseline Characteristics and Technology Training of Underserved Adults With Type 2 Diabetes in the Mobile Diabetes Detective (MoDD) Randomized Controlled Trial [J]. Diabetes Educ, 2017, 43(6): 576-88.

[107] HIDRUS A, KUEH Y C, NORSAáDAH B, et al. Effects of brain breaks videos on the motives for the physical activity of Malaysians with type-2 diabetes mellitus [J]. International journal of environmental research and public health, 2020, 17(7).

[108] IDREES T, CASTRO-REVOREDO I A, OH H D, et al. Continuous Glucose Monitoring-Guided Insulin Administration in Long-Term Care Facilities: A Randomized Clinical Trial [J]. Journal of the American Medical Directors Association, 2024, 25(5): 884-8.

[109] IHEKORONYE M R, OSEMENE K P, OAMEN T E. Pharmacist-led intervention to improve treatment outcomes in type 2 diabetes: a randomized controlled trial [J]. J Pharm Health Serv Res, 2024, 15(2).

[110] IZQUIERDO R, LAGUA C T, MEYER S, et al. Telemedicine intervention effects on waist circumference and body mass index in the IDEATel project [J]. Diabetes technology & therapeutics, 2010, 12(3): 213-20.

[111] JAYASREE B, STALIN P. Efficacy of behavior change communication using mobile calls on glycemic control among Type 2 diabetic patients in an urban area of Pondicherry, South India: A randomized controlled trial [J]. J Edu Health Promotion, 2019, 8(1).

[112] JIA W, ZHANG P, ZHU D, et al. Evaluation of an mHealth-enabled hierarchical diabetes management intervention in primary care in China (ROADMAP): A cluster randomized trial [J]. PLoS medicine, 2021, 18(9): e1003754.

[113] JOHNSON E J, NILES B L, MORI D L. Targeted recruitment of adults with type 2 diabetes for a physical activity intervention [J]. Diabetes Spectr, 2015, 28(2): 99-105.

[114] KANG J, CHEN Y, ZHAO Y, et al. Effect of remote management on comprehensive management of diabetes mellitus during the COVID-19 epidemic [J]. Primary care diabetes, 2021, 15(3): 417-23.

[115] KEMPF K, RöHLING M, BANZER W, et al. High-Protein, Low-Glycaemic Meal Replacement Decreases Fasting Insulin and Inflammation Markers-A 12-Month Subanalysis of the ACOORH Trial [J]. Nutrients, 2021, 13(5).

[116] KERFOOT B P, GAGNON D R, MCMAHON G T, et al. A team-based online game improves blood glucose control in veterans with type 2 diabetes: A randomized controlled trial [J]. Diabetes care, 2017, 40(9): 1218-25.

[117] KHUNTI K, GRIFFIN S, BRENNAN A, et al. Promoting physical activity in a multi-ethnic population at high risk of diabetes: the 48-month PROPELS randomised controlled trial [J]. BMC Med, 2021, 19(1).

[118] KOBAYASHI T, TSUSHITA K, NOMURA E, et al. Automated Feedback Messages With Shichifukujin Characters Using IoT System-Improved Glycemic Control in People With Diabetes: A Prospective, Multicenter Randomized Controlled Trial [J]. Journal of diabetes science and technology, 2019, 13(4): 796-8.

[119] KU E J, PARK J I, JEON H J, et al. Clinical efficacy and plausibility of a smartphone-based integrated online real-time diabetes care system via glucose and diet data management: a pilot study [J]. Intern Med J, 2020, 50(12): 1524-32.

[120] KUMAR D, RAINA S, SHARMA S B, et al. Effectiveness of randomized control trial of mobile phone messages on control of fasting blood glucose in patients with type-2 diabetes mellitus in a Northern State of India [J]. Indian J Public Health, 2018, 62(3): 224-6.

[121] LEE J Y, LEE S W, NASIR N H, et al. Diabetes telemonitoring reduces the risk of hypoglycaemia during Ramadan: a pilot randomized controlled study [J]. Diabetic medicine : a journal of the British Diabetic Association, 2015, 32(12): 1658-61.

[122] LUCHSINGER J A, PALMAS W, TERESI J A, et al. Improved diabetes control in the elderly delays global cognitive decline [J]. The journal of nutrition, health & aging, 2011, 15(6): 445-9.

[123] MACPHERSON M M, MERRY K J, LOCKE S R, et al. Effects of mobile health prompts on self-monitoring and exercise behaviors following a diabetes prevention program: Secondary analysis from a randomized controlled trial [J]. JMIR mHealth and uHealth, 2019, 7(9).

[124] MAYBERRY L S, BERG C A, GREEVY R A, et al. Mixed-Methods Randomized Evaluation of FAMS: A Mobile Phone-Delivered Intervention to Improve Family/Friend Involvement in Adults' Type 2 Diabetes Self-Care [J]. Annals of behavioral medicine : a publication of the Society of Behavioral Medicine, 2021, 55(2): 165-78.

[125] MCCLINTOCK H F D, MORALES K H, SMALL D S, et al. A brief adherence intervention that improved glycemic control: mediation by patterns of adherence [J]. Journal of behavioral medicine, 2015, 38(1): 39-47.

[126] MORRISON J, AKTER K, JENNINGS H, et al. Learning from a diabetes mHealth intervention in rural Bangladesh: what worked, what did not and what next? [J]. Global Public Health, 2022, 17(7): 1299-313.

[127] MURALIDHARAN S, RANJANI H, ANJANA R M, et al. Change in cardiometabolic risk factors among Asian Indian adults recruited in a mHealth-based diabetes prevention trial [J]. Digit Health, 2021, 7.

[128] MURALIDHARAN S, RANJANI H, ANJANA R M, et al. Engagement and Weight Loss: Results from the Mobile Health and Diabetes Trial [J]. Diabetes technology & therapeutics, 2019, 21(9): 507-13.

[129] NELSON L A, SPIEKER A J, GREEVY R A, et al. Glycemic outcomes of a family-focused intervention for adults with type 2 diabetes: Main, mediated, and subgroup effects from the FAMS 2.0 RCT [Z]. 2023.10.1101/2023.09.11.23295374

[130] NELSON L A, WILLIAMSON S E, LESTOURGEON L M, et al. Retaining diverse adults with diabetes in a long-term trial: Strategies, successes, and lessons learned [J]. Contemporary clinical trials, 2021, 105: 106388.

[131] ORON T, FARFEL A, MULLER I, et al. A remote monitoring system for artificial pancreas support is safe, reliable, and user friendly [J]. Diabetes Technol Ther, 2014, 16(11): 699-705.

[132] PACAUD D, KELLEY H, DOWNEY A M, et al. Successful delivery of diabetes self-care education and follow-up through ehealth media [J]. Canadian journal of diabetes, 2012, 36(5): 257-62.

[133] PATNAIK L, PANIGRAHI S K, SAHOO A K, et al. Effectiveness of mobile application for promotion of physical activity among newly diagnosed patients of type II diabetes -A randomized controlled trial [J]. Int J Prev Med, 2022, 13(1): 54.

[134] PEACOCK O J, WESTERN M J, BATTERHAM A M, et al. Effect of novel technology-enabled multidimensional physical activity feedback in primary care patients at risk of chronic disease - The MIPACT study: A randomised controlled trial [J]. Int J Behav Nutr Phys Act, 2020, 17(1).

[135] PITT S, SJöBLOM L, BäLTER K, et al. The effect of an app-based dietary intervention on diet-related greenhouse gas emissions – results from a randomized controlled trial [J]. Int J Behav Nutr Phys Act, 2023, 20(1).

[136] PLOTNIKOFF R C, WILCZYNSKA M, COHEN K E, et al. Integrating smartphone technology, social support and the outdoor physical environment to improve fitness among adults at risk of, or diagnosed with, Type 2 Diabetes: Findings from the ‘eCoFit’ randomized controlled trial [J]. Prev Med, 2017, 105: 404-11.

[137] POIRIER J, BENNETT W L, JEROME G J, et al. Effectiveness of an Activity Tracker- and Internet-Based Adaptive Walking Program for Adults: A Randomized Controlled Trial [J]. Journal of medical Internet research, 2016, 18(2): e34.

[138] POPPE L, BOURDEAUDHUIJ I D, VERLOIGNE M, et al. Efficacy of a Self-Regulation-Based Electronic and Mobile Health Intervention Targeting an Active Lifestyle in Adults Having Type 2 Diabetes and in Adults Aged 50 Years or Older: Two Randomized Controlled Trials [J]. Journal of medical Internet research, 2019, 21(8): N.PAG-N.PAG.

[139] QUINN C C, SAREH P L, SHARDELL M L, et al. Mobile diabetes intervention for glycemic control: Impact on physician prescribing [J]. Journal of diabetes science and technology, 2014, 8(2): 362-70.

[140] QUINN C C, SWASEY K K, CRABBE J C F, et al. The Impact of a Mobile Diabetes Health Intervention on Diabetes Distress and Depression Among Adults: Secondary Analysis of a Cluster Randomized Controlled Trial [J]. JMIR mHealth and uHealth, 2017, 5(12).

[141] QUINN C C, SWASEY K K, TORAIN J M, et al. An mHealth Diabetes Intervention for Glucose Control: Health Care Utilization Analysis [J]. JMIR mHealth and uHealth, 2018, 6(10).

[142] RAMACHANDRAN A, SNEHALATHA C, RAM J, et al. Effectiveness of mobile phone messaging in prevention of type 2 diabetes by lifestyle modification in men in India: A prospective, parallel-group, randomised controlled trial [J]. Lancet Diabetes Endocrinol, 2013, 1(3): 191-8.

[143] RAMOS-ZAVALA M G, GROVER-PáEZ F, CARDONA-MUñOZ E G, et al. Comparison of the use of blood pressure telemonitoring versus standard medical care in the achievement of short-term therapeutic goals in blood pressure in patients with uncontrolled hypertension: An open-label clinical trial [J]. JRSM Cardiovascular Disease, 2023, 12.

[144] RHO M J, KIM S R, KIM H S, et al. Exploring the relationship among user satisfaction, compliance, and clinical outcomes of telemedicine services for glucose control [J]. Telemedicine e-Health, 2014, 20(8): 712-20.

[145] RODDY M K K, NELSON L A, GREEVY R A, et al. Changes in family involvement occasioned by FAMS mobile health intervention mediate changes in glycemic control over 12 months [J]. Journal of behavioral medicine, 2022, 45(1): 28-37.

[146] ROMERO-AROCA P, VERGES R, MAAROF N, et al. Real-world outcomes of a clinical decision support system for diabetic retinopathy in Spain [J]. BMJ Open Ophthalmology, 2022, 7(1).

[147] SáNCHEZ A, SILVESTRE C, CAMPO N, et al. Type-2 diabetes primary prevention program implemented in routine primary care: A process evaluation study [J]. Trials, 2016, 17(1).

[148] SHAHABI N, KOLIVAND M, SALARI N, et al. The effect of telenursing training based on family-centered empowerment pattern on compliance with diet regimen in patients with diabetes mellitus type 2: a randomized clinical trial [J]. BMC endocrine disorders, 2022, 22(1): 36.

[149] SIMON S R, TRINACTY C M, SOUMERAI S B, et al. Improving diabetes care among patients overdue for recommended testing: A randomized controlled trial of automated telephone outreach [J]. Diabetes care, 2010, 33(7): 1452-3.

[150] SINGH L G, LEVITT D L, SATYARENGGA M, et al. Continuous Glucose Monitoring in General Wards for Prevention of Hypoglycemia: Results From the Glucose Telemetry System Pilot Study [J]. Journal of diabetes science and technology, 2020, 14(4): 783-90.

[151] TUELUECE D, DIKICI C, SERIN E K. The effect of education of patients with type 2 diabetes at risk of covid-19 on symptoms and some metabolic outcomes: A randomized controlled study [J]. Primary care diabetes, 2023, 17(1): 3-11.

[152] VAN DEN HELDER J, MEHRA S, VAN DRONKELAAR C, et al. Blended home-based exercise and dietary protein in community-dwelling older adults: a cluster randomized controlled trial [J]. Journal of Cachexia, Sarcopenia and Muscle, 2020, 11(6): 1590-602.

[153] VAN DER WEEGEN S, VERWEY R, SPREEUWENBERG M, et al. It's LiFe! Mobile and Web-Based Monitoring and Feedback Tool Embedded in Primary Care Increases Physical Activity: A Cluster Randomized Controlled Trial [J]. Journal of medical Internet research, 2015, 17(7): e184.

[154] VAN OLMEN J, KEGELS G, KORACHAIS C, et al. The effect of text message support on diabetes self-management in developing countries – A randomised trial [J]. J Clin Transl Endocrinol, 2017, 7: 33-41.

[155] WAKI K, AIZAWA K, KATO S, et al. Dial betics with a multimedia food recording tool, food log: Smartphone-based self-management for type 2 diabetes [J]. Journal of diabetes science and technology, 2015, 9(3): 534-40.

[156] WALLER K A, KILLEDAR A A, FURBER S E, et al. Economic evaluation of a mobile phone text-message intervention for Australian adults with type 2 diabetes [J]. mHealth, 2023, 9.

[157] WU C J J, SUNG H C, CHANG A M, et al. Cardiac-diabetes self-management program for Australians and Taiwanese: A randomized blocked design study [J]. Nurs Health Sci, 2017, 19(3): 307-15.

[158] YOUNG R J, BURNS E, TAYLOR J, et al. Pro-active call center treatment support (PACCTS) to improve glucose control in type 2 diabetes - A randomized controlled trial [J]. Diabetes care, 2005, 28(2): 278-82.

[159] ALCáNTARA-ARAGóN V, RODRIGO-CANO S, LUPIANEZ-BARBERO A, et al. Web Support for Weight-Loss Interventions: PREDIRCAM2 Clinical Trial Baseline Characteristics and Preliminary Results [J]. Diabetes Technol Ther, 2018, 20(5): 380-5.

[160] ANDERSON D R, CHRISTISON-LAGAY J, VILLAGRA V, et al. Managing the space between visits: a randomized trial of disease management for diabetes in a community health center [J]. Journal of general internal medicine, 2010, 25(10): 1116-22.

[161] ASANTE E, BAM V, DIJI A K A, et al. Pilot Mobile Phone Intervention in Promoting Type 2 Diabetes Management in an Urban Area in Ghana: A Randomized Controlled Trial [J]. Diabetes Educ, 2020, 46(5): 455-64.

[162] BENSON G A, SIDEBOTTOM A, HAYES J, et al. Impact of ENHANCED (diEtitiaNs Helping pAtieNts CarE for Diabetes) Telemedicine Randomized Controlled Trial on Diabetes Optimal Care Outcomes in Patients with Type 2 Diabetes [J]. Journal of the Academy of Nutrition and Dietetics, 2019, 119(4): 585-98.

[163] BIERMANN E, DIETRICH W, RIHL J, et al. Are there time and cost savings by using telemanagement for patients on intensified insulin therapy?: A randomised, controlled trial [J]. Comput Methods Programs Biomed, 2002, 69(2): 137-46.

[164] BIERMANN E, DIETRICH W, STANDL E. Telecare of diabetic patients with intensified insulin therapy; proceedings of the Studies in health technology and informatics, F, 2000 [C]. 2001134190.

[165] BLACKBERRY I D, FURLER J S, BEST J D, et al. Effectiveness of general practice based, practice nurse led telephone coaching on glycaemic control of type 2 diabetes: The Patient Engagement and Coaching for Health (PEACH) pragmatic cluster randomised controlled trial [J]. BMJ (Online), 2013, 347(7926).

[166] BLIOUMPA C, KARANASIOU E, ANTONIOU V, et al. Efficacy of supervised home-based, real time, videoconferencing telerehabilitation in patients with type 2 diabetes: a single-blind randomized controlled trial [J]. European journal of physical and rehabilitation medicine, 2023, 59(5): 628-39.

[167] BONN S E, HUMMEL M, PEVERI G, et al. Effectiveness of a Smartphone App to Promote Physical Activity Among Persons With Type 2 Diabetes: Randomized Controlled Trial [J]. Interactive Journal of Medical Research, 2024, 13.

[168] BRADWAY M, PFUHL G, JOAKIMSEN R, et al. Analysing mHealth usage logs in RCTs: Explaining participants' interactions with type 2 diabetes self-management tools [J]. PloS one, 2018, 13(8).

[169] BUJNOWSKA-FEDAK M M, PUCHALA E, STECIWKO A. The Impact of Telehome Care on Health Status and Quality of Life Among Patients with Diabetes in a Primary Care Setting in Poland [J]. Telemedicine e-Health, 2011, 17(3): 153-63.

[170] CALIKOGLU F, BAGDEMIR E, CELIK S, et al. Telemedicine as a Motivational Tool to Optimize Metabolic Control in Patients with Diabetes in Turkey: A Prospective, Randomized, Controlled TeleDiab Trial [J]. Telemedicine e-Health, 2023, 29(4): 518-30.

[171] CARRASQUILLO O, LEBRON C, ALONZO Y, et al. Effect of a community health worker intervention among Latinos with poorly controlled type 2 diabetes: The miami healthy heart initiative randomized clinical trial [J]. JAMA internal medicine, 2017, 177(7): 948-54.

[172] CARTER E L, NUNLEE-BLAND G, CALLENDER C. A patient-centric, provider-assisted diabetes telehealth self-management intervention for urban minorities [J]. Perspect Health Inf Manag, 2011, 8: 1b.

[173] CHAMANY S, WALKER E A, SCHECHTER C B, et al. Telephone Intervention to Improve Diabetes Control: A Randomized Trial in the New York City A1c Registry [J]. American journal of preventive medicine, 2015, 49(6): 832-41.

[174] CHANTIRA C, SARANYA C, WARITHORN P, et al. Two-way SMS Reminders for Medication Adherence and Quality of Life in Adults with Type 2 Diabetes: A Randomized Controlled Trial [J]. Pacific Rim Intl J Nurs Res, 2023, 27(3): 457-71.

[175] CHO J H, CHOI Y H, KIM H S, et al. Effectiveness and safety of a glucose data-filtering system with automatic response software to reduce the physician workload in managing type 2 diabetes [J]. Journal of telemedicine and telecare, 2011, 17(5): 257-62.

[176] CHO J H, KWON H S, KIM H S, et al. Effects on diabetes management of a health-care provider mediated, remote coaching system via a PDA-type glucometer and the Internet [J]. Journal of telemedicine and telecare, 2011, 17(7): 365-70.

[177] CHRISTENSEN M B, SERIFOVSKI N, HERZ A M H, et al. Efficacy of Bolus Calculation and Advanced Carbohydrate Counting in Type 2 Diabetes: A Randomized Clinical Trial [J]. Diabetes Technol Ther, 2021, 23(2): 95-103.

[178] CROWLEY M J, EDELMAN D, MCANDREW A T, et al. Practical Telemedicine for Veterans with Persistently Poor Diabetes Control: A Randomized Pilot Trial [J]. Telemedicine journal and e-health : the official journal of the American Telemedicine Association, 2016, 22(5): 376-84.

[179] CROWLEY M J, POWERS B J, OLSEN M K, et al. The cholesterol, hypertension, and glucose education (CHANGE) study: Results from a randomized controlled trial in African Americans with diabetes [J]. Am Heart J, 2013, 166(1): 179-86.e2.

[180] DE VASCONCELOS H C A, LIRA NETO J C G, DE ARAúJO M F M, et al. Telecoaching programme for type 2 diabetes control: A randomised clinical trial [J]. Brit J Nurs, 2018, 27(19): 1115-20.

[181] DEL PRATO S, NICOLUCCI A, LOVAGNINI-SCHER A C, et al. Telecare Provides Comparable Efficacy to Conventional Self-Monitored Blood Glucose in Patients with Type 2 Diabetes Titrating One Injection of Insulin Glulisine-the ELEONOR Study [J]. Diabetes technology & therapeutics, 2012, 14(2): 175-82.

[182] DöBLER A, BELNAP B H, POLLMANN H, et al. Telephone-delivered lifestyle support with action planning and motivational interviewing techniques to improve rehabilitation outcomes [J]. Rehabilitation psychology, 2018, 63(2): 170-81.

[183] DURUTURK N, ÖZKöSLü M A. Effect of tele-rehabilitation on glucose control, exercise capacity, physical fitness, muscle strength and psychosocial status in patients with type 2 diabetes: A double blind randomized controlled trial [J]. Primary care diabetes, 2019, 13(6): 542-8.

[184] FORTMANN A L, GALLO L C, GARCIA M I, et al. Dulce digital: An mHealth SMS based intervention improves glycemic control in hispanics with type 2 diabetes [J]. Diabetes care, 2017, 40(10): 1349-55.

[185] FRANCO D W, ALESSI J, CARVALHO T R D, et al. The impact of a telehealth intervention on the metabolic profile of diabetes mellitus patients during the COVID-19 pandemic-A randomized clinical trial [J]. Primary care diabetes, 2022, 16(6): 745-52.

[186] GAGLIARDINO J J, ARRECHEA V, ASSAD D, et al. Type 2 diabetes patients educated by other patients perform at least as well as patients trained by professionals [J]. Diabetes Metab Res Rev, 2013, 29(2): 152-60.

[187] GILLANI S W. Determining effective diabetic care; A multicentre - Longitudinal interventional study [J]. Curr Pharm Des, 2016, 22: 6469-76.

[188] GONZALEZ J S, HOOGENDOORN C J, SCHECHTER C B, et al. Outcomes of New York City Care Calls: A Prospective Randomized Controlled Effectiveness Trial of Telephone-Delivered Type 2 Diabetes Self-Management Support [J]. The science of diabetes self-management and care, 2024, 50(3): 235-49.

[189] GOODARZI M, EBRAHIMZADEH I, RABI A, et al. Impact of distance education via mobile phone text messaging on knowledge, attitude, practice and self efficacy of patients with type 2 diabetes mellitus in Iran [J]. J Diabetes Metabolic Disord, 2012, 11(1): 1-8.

[190] GORDON H S, PUGACH O, SOLANKI P, et al. A brief pre-visit educational video improved patient engagement after telehealth visits; results from a randomized controlled trial [J]. PEC Innov, 2022, 1.

[191] GUNAWARDENA K C, JACKSON R, ROBINETT I, et al. The Influence of the Smart Glucose Manager Mobile Application on Diabetes Management [J]. Journal of diabetes science and technology, 2019, 13(1): 75-81.

[192] GUO M, MENG F, GUO Q, et al. Effectiveness of mHealth management with an implantable glucose sensor and a mobile application among Chinese adults with type 2 diabetes [J]. Journal of telemedicine and telecare, 2023, 29(8): 632-40.

[193] GUPTA U, GUPTA Y, JOSE D, et al. Effectiveness of a Video-Based Lifestyle Education Program Compared to Usual Care in Improving HbA1c and Other Metabolic Parameters in Individuals with Type 2 Diabetes: An Open-Label Parallel Arm Randomized Control Trial (RCT) [J]. Diabetes Ther, 2020, 11(3): 667-79.

[194] HAIDER R, HYUN K, CHEUNG N W, et al. Effect of lifestyle focused text messaging on risk factor modification in patients with diabetes and coronary heart disease: A sub-analysis of the TEXT ME study [J]. Diabetes research and clinical practice, 2019, 153: 184-90.

[195] HAN C Y, ZHANG J, YE X M, et al. Telemedicine-assisted structured self-monitoring of blood glucose in management of T2DM results of a randomized clinical trial [J]. BMC medical informatics and decision making, 2023, 23(1): 182.

[196] HARNO K, KAUPPINEN-MäKELIN R, SYRJäLäINEN J. Managing diabetes care using an integrated regional e-health approach [J]. Journal of telemedicine and telecare, 2006, 12 Suppl 1: 13-5.

[197] HEALD A H, ROBERTS S, GIMENO L A, et al. A Randomised Control Trial to Explore the Impact and Efficacy of the Healum Collaborative Care Planning Software and App on Condition Management in the Type 2 Diabetes Mellitus Population in NHS Primary Care [J]. Diabetes Ther, 2023, 14(6): 977-88.

[198] HEISLER M, CHOI H, PALMISANO G, et al. Comparison of community health worker-led diabetes medication decision-making support for low-income latino and african american adults with diabetes using E-health tools versus print materials [J]. Annals of internal medicine, 2014, 161: S13-S22.

[199] HERMANNS N, EHRMANN D, FINKE-GROENE K, et al. Use of smartphone application versus written titration charts for basal insulin titration in adults with type 2 diabetes and suboptimal glycaemic control (My Dose Coach): multicentre, open-label, parallel, randomised controlled trial [J]. Lancet Reg Health Eur, 2023, 33.

[200] HILMARSDóTTIR E, SIGURðARDóTTIR Á K, ARNARDóTTIR R H. A Digital Lifestyle Program in Outpatient Treatment of Type 2 Diabetes: A Randomized Controlled Study [J]. Journal of diabetes science and technology, 2020, 15(5): 1134-41.

[201] HU Y, WEN X, NI L, et al. Effects of telemedicine intervention on the management of diabetic complications in type 2 diabetes [J]. Int J Diabetes Dev Countries, 2021, 41(2): 322-8.

[202] ILJAZ R, BRODNIK A, ZRIMEC T, et al. E-HEALTHCARE for DIABETES MELLITUS TYPE 2 PATIENTS - A RANDOMISED CONTROLLED TRIAL in SLOVENIA [J]. Zdravstveno Varstvo, 2017, 56(3): 150-7.

[203] IZQUIERDO R E, KNUDSON P E, MEYER S, et al. A comparison of diabetes education administered through telemedicine versus in person [J]. Diabetes care, 2003, 26(4): 1002-7.

[204] JAIN V, JOSHI R, IDICULLA J, et al. Community health worker interventions in type 2 diabetes mellitus patients: Assessing the feasibility and effectiveness in Rural Central India [J]. Journal of Cardiovascular Disease Research, 2018, 9(3): 127-33.

[205] JIANG Y, RAMACHANDRAN H J, TEO J Y C, et al. Effectiveness of a nurse-led smartphone-based self-management programme for people with poorly controlled type 2 diabetes: A randomized controlled trial [J]. Journal of advanced nursing, 2022, 78(4): 1154-65.

[206] KARGARSHUROKI M, SADEGHIAN H A, FATEHI F, et al. The effect of diabetes training through social networks on metabolic control of individuals with type 2 diabetes; a randomized controlled trial [J]. Journal of preventive medicine and hygiene, 2023, 64(4): E499-e506.

[207] KARHULA T, VUORINEN A L, RääPYSJäRVI K, et al. Telemonitoring and Mobile Phone-Based Health Coaching Among Finnish Diabetic and Heart Disease Patients: Randomized Controlled Trial [J]. Journal of medical Internet research, 2015, 17(6): e153.

[208] KIM C S, PARK S Y, KANG J G, et al. Insulin dose titration system in diabetes patients using a short messaging service automatically produced by a knowledge matrix [J]. Diabetes technology & therapeutics, 2010, 12(8): 663-9.

[209] KIM H. A randomized controlled trial of a nurse short-message service by cellular phone for people with diabetes [J]. International journal of nursing studies, 2007, 44(5): 687-92.

[210] KIM H S, JEONG H S. A nurse short message service by cellular phone in type-2 diabetic patients for six months [J]. J Clin Nurs, 2007, 16(6): 1082-7.

[211] KIM H S, OH J A. Adherence to diabetes control recommendations: impact of nurse telephone calls [J]. Journal of advanced nursing, 2003, 44(3): 256-61.

[212] KIM H S, SUN C, YANG S J, et al. Randomized, Open-Label, Parallel Group Study to Evaluate the Effect of Internet-Based Glucose Management System on Subjects with Diabetes in China [J]. Telemedicine journal and e-health : the official journal of the American Telemedicine Association, 2016, 22(8): 666-74.

[213] LAKKA T A, AITTOLA K, JäRVELä-REIJONEN E, et al. Real-world effectiveness of digital and group-based lifestyle interventions as compared with usual care to reduce type 2 diabetes risk – A stop diabetes pragmatic randomised trial [J]. Lancet Reg Health Eur, 2023, 24.

[214] LEE C S, TYAGI S, LING KOH E Y, et al. Health outcomes of telemonitoring of patients with type-2 diabetes mellitus: One-year results from a randomized controlled trial (Optimizing care of Patients via Telemedicine In Monitoring and aUgmenting their control of diabetes Mellitus) [J]. Journal of telemedicine and telecare, 2024.

[215] LEE S E, PARK S K, PARK Y S, et al. Effects of Short-term Mobile Application Use on Weight Reduction for Patients with Type 2 Diabetes [J]. J Obe Met Synd, 2021, 30(4): 345-53.

[216] LEICHTER S B, BOWMAN K, ADKINS R A, et al. Impact of remote management of diabetes via computer: The 360 study - A proof-of-concept randomized trial [J]. Diabetes Technol Ther, 2013, 15(5): 434-8.

[217] LIM S, KANG S M, KIM K M, et al. Multifactorial intervention in diabetes care using real-time monitoring and tailored feedback in type 2 diabetes [J]. Acta diabetologica, 2016, 53(2): 189-98.

[218] LIM S, KANG S M, SHIN H, et al. Improved Glycemic Control Without Hypoglycemia in Elderly Diabetic Patients Using the Ubiquitous Healthcare Service, a New Medical Information System [J]. Diabetes care, 2011, 34(2): 308-13.

[219] LIN K, ZHANG W, HE F, et al. Evaluation of the Clinical Efficacy of the Treatment of Overweight and Obesity in Type 2 Diabetes Mellitus by the Telemedicine Management System Based on the Internet of Things Technology [J]. Comput Intell Neurosci, 2022, 2022.

[220] LU Z, LI Y, HE Y, et al. Internet-Based Medication Management Services Improve Glycated Hemoglobin Levels in Patients with Type 2 Diabetes [J]. Telemedicine journal and e-health : the official journal of the American Telemedicine Association, 2021, 27(6): 686-93.

[221] MAISLOS M, WEISMAN D. Multidisciplinary approach to patients with poorly controlled type 2 diabetes mellitus: A prospective, randomized study [J]. Acta diabetologica, 2004, 41(2): 44-8.

[222] MASLAKPAK M H, RAZMARA S, NIAZKHANI Z. Effects of Face-to-Face and Telephone-Based Family-Oriented Education on Self-Care Behavior and Patient Outcomes in Type 2 Diabetes: A Randomized Controlled Trial [J]. Journal of diabetes research, 2017, 2017.

[223] MIRANDA L D S P, EZEQUIEL D G A, VANELLI C P, et al. Impact of an educational intervention in the management of individuals with uncontrolled type 2 diabetes mellitus using insulin therapy [J]. Primary care diabetes, 2022, 16(4): 496-501.

[224] MORADI A, ALAVI S M, SALIMI M, et al. The effect of short message service (SMS) on knowledge and preventive behaviors of diabetic foot ulcer in patients with diabetes type 2 [J]. Diabetes Metab Syndr Clin Res Rev, 2019, 13(2): 1255-60.

[225] NEKO F Z, ZEIDI I M, MORSHEDI H, et al. Effectiveness of Theory-Based Intervention on Knowledge Level, Psychological Constructs, Metabolic Index and Physical Activity Status in Patients with Type 2 Diabetes: Application of the Health Action Process Approach (HAPA) Model [J]. Clin Diabetol, 2023, 12(5): 290-300.

[226] NICOLUCCI A, CERCONE S, CHIRIATTI A, et al. A Randomized Trial on Home Telemonitoring for the Management of Metabolic and Cardiovascular Risk in Patients with Type 2 Diabetes [J]. Diabetes technology & therapeutics, 2015, 17(8): 563-70.

[227] ODNOLETKOVA I, GODERIS G, NOBELS F, et al. Optimizing diabetes control in people with Type 2 diabetes through nurse-led telecoaching [J]. Diabetic medicine : a journal of the British Diabetic Association, 2016, 33(6): 777-85.

[228] PAMUNGKAS R A, USMAN A M, CHAMROONSAWASDI K. A smartphone application of diabetes coaching intervention to prevent the onset of complications and to improve diabetes self-management: A randomized control trial [J]. Diabetes Metab Syndr Clin Res Rev, 2022, 16(7).

[229] PARK G, LEE H, LEE Y, et al. Automated Personalized Self-care Program for Patients With Type 2 Diabetes Mellitus: A Pilot Trial * [J]. Asian Nurs Res, 2024, 18(2): 114-24.

[230] PARK S, PARK J H. Effects of digital self-care intervention for Korean older adults with type 2 diabetes: A randomized controlled trial over 12 weeks [J]. Geriatric nursing (New York, NY), 2024, 58: 155-61.

[231] QUINN C C, CLOUGH S S, MINOR J M, et al. WellDoc™ mobile diabetes management randomized controlled trial: Change in clinical and behavioral outcomes and patient and physician satisfaction [J]. Diabetes Technol Ther, 2008, 10(3): 160-8.

[232] QUINN C C, SHARDELL M D, TERRIN M L, et al. Mobile Diabetes Intervention for Glycemic Control in 45- to 64-Year-Old Persons with Type 2 Diabetes [J]. J Appl Gerontol, 2016, 35(2): 227-43.

[233] RAMADAS A, CHAN C K Y, OLDENBURG B, et al. Randomised-controlled trial of a web-based dietary intervention for patients with type 2 diabetes: Changes in health cognitions and glycemic control [J]. BMC public health, 2018, 18(1).

[234] RASMUSSEN O W, LAUSZUS F F, LOEKKE M. Telemedicine compared with standard care in type 2 diabetes mellitus: A randomized trial in an outpatient clinic [J]. Journal of telemedicine and telecare, 2016, 22(6): 363-8.

[235] RIANGKAM C, SRIYUKTASUTH A, PONGTHAVORNKAMOL K, et al. Effects of a mobile health diabetes self-management program on HbA1C, self-management and patient satisfaction in adults with uncontrolled type 2 diabetes: a randomized controlled trial [J]. Journal of Health Research, 2022, 36(5): 878-88.

[236] ROVNER B W, CASTEN R, CHANG A M, et al. Interprofessional Intervention to Reduce Emergency Department Visits in Black Individuals with Diabetes [J]. Population Health Management, 2023, 26(1): 46-52.

[237] RUISSEN M, TORRES-PEñA J D, UITBEIJERSE B, et al. An integrated e-health support of shared decision making and self-management (POWER2DM) for patients with diabetes mellitus and their healthcare professionals [J]. Atherosclerosis, 2022, 355: 23.

[238] SAITO R, SZE W T, WAKI K, et al. Effect of Step Count Measurement on Glycemic Control: Secondary Analysis of a Randomized Controlled Trial; proceedings of the Studies in health technology and informatics, F, 2024 [C]. 38269869.

[239] SHAHID M, MAHAR S A, SHAIKH S, et al. Mobile phone intervention to improve diabetes care in rural areas of Pakistan: A randomized controlled trial [J]. J Coll Phys Surg Pak, 2015, 25(3): 166-71.

[240] SHAO Y X, SHI L Z, NAUMAN E, et al. Telehealth use and its impact on clinical outcomes in patients with type 2 diabetes during the COVID-19 pandemic [J]. Diabetes Obesity & Metabolism, 2024, 26(1): 118-25.

[241] SOKOLOVSKA J, OSTROVSKA K, PAHIRKO L, et al. Impact of interval walking training managed through smart mobile devices on albuminuria and leptin/adiponectin ratio in patients with type 2 diabetes [J]. Physiol Rep, 2020, 8(13).

[242] STEVENTON A, BARDSLEY M, DOLL H, et al. Effect of telehealth on glycaemic control: analysis of patients with type 2 diabetes in the Whole Systems Demonstrator cluster randomised trial [J]. BMC health services research, 2014, 14: 334.

[243] SUNIL KUMAR D, PRAKASH B, SUBHASH CHANDRA B J, et al. Technological innovations to improve health outcome in type 2 diabetes mellitus: A randomized controlled study [J]. Clin Epidemiol Global Health, 2021, 9: 53-6.

[244] TAN N C, TYAGI S, LEE C S, et al. Effectiveness of an algorithm-driven home telemonitoring system on the metabolic control and self-care behaviour of Asian adults with type-2 diabetes mellitus: A randomised controlled trial [J]. Journal of telemedicine and telecare, 2023.

[245] TAN S H X, ANG S B, TAN N C, et al. Cost-Effectiveness of a Home Telemonitoring System for Asian Adults with Type 2 Diabetes Mellitus [J]. Telemedicine journal and e-health : the official journal of the American Telemedicine Association, 2024, 30(8): 2353-62.

[246] TILDESLEY H D, WRIGHT A M, CHAN J H M, et al. A comparison of internet monitoring with continuous glucose monitoring in insulin-requiring type 2 diabetes mellitus [J]. Canadian journal of diabetes, 2013, 37(5): 305-8.

[247] VINITHA R, NANDITHA A, SNEHALATHA C, et al. Effectiveness of mobile phone text messaging in improving glycaemic control among persons with newly detected type 2 diabetes [J]. Diabetes research and clinical practice, 2019, 158.

[248] VON STORCH K, GRAAF E, WUNDERLICH M, et al. Telemedicine-Assisted Self-Management Program for Type 2 Diabetes Patients [J]. Diabetes Technol Ther, 2019, 21(9): 514-21.

[249] WAKEFIELD B J, HOLMAN J E, RAY A, et al. Effectiveness of home telehealth in comorbid diabetes and hypertension: a randomized, controlled trial [J]. Telemedicine journal and e-health : the official journal of the American Telemedicine Association, 2011, 17(4): 254-61.

[250] WAKI K, FUJITA H, UCHIMURA Y, et al. DialBetics: A novel smartphone-based self-management support system for type 2 diabetes patients; proceedings of the Journal of diabetes science and technology, F, 2014 [C].

[251] WANG G, ZHANG Z, FENG Y, et al. Telemedicine in the Management of Type 2 Diabetes Mellitus [J]. Am J Med Sci, 2017, 353(1): 1-5.

[252] WANG J, CAI C, PADHYE N, et al. A behavioral lifestyle intervention enhanced with multiple-behavior self-monitoring using mobile and connected tools for underserved individuals with type 2 diabetes and comorbid overweight or obesity: Pilot comparative effectiveness trial [J]. JMIR mHealth and uHealth, 2018, 6(4).

[253] WANG Y, LI M, ZHAO X, et al. Effects of continuous care for patients with type 2 diabetes using mobile health application: A randomised controlled trial [J]. Int J Health Plann Manage, 2019, 34(3): 1025-35.

[254] WARREN R, CARLISLE K, MIHALA G, et al. Effects of telemonitoring on glycaemic control and healthcare costs in type 2 diabetes: A randomised controlled trial [J]. Journal of telemedicine and telecare, 2018, 24(9): 586-95.

[255] WEI N J, NATHAN D M, WEXLER D J. Glycemic control after hospital discharge in insulin-treated type 2 diabetes: A randomized pilot study of daily remote glucose monitoring [J]. Endocr Pract, 2015, 21(2): 115-21.

[256] WELCH G, ALLEN N A, ZAGARINS S E, et al. Comprehensive diabetes management program for poorly controlled Hispanic type 2 patients at a community health center [J]. The Diabetes educator, 2011, 37(5): 680-8.

[257] WIDYANATA K A J, ARIFIN H. DM-calendar app as a diabetes self-management education on adult type 2 diabetes mellitus: a randomized controlled trial [J]. J Diabetes Metabolic Disord, 2019, 18(2): 557-63.

[258] WILD S H, HANLEY J, LEWIS S C, et al. Supported Telemonitoring and Glycemic Control in People with Type 2 Diabetes: The Telescot Diabetes Pragmatic Multicenter Randomized Controlled Trial [J]. PLoS medicine, 2016, 13(7): e1002098.

[259] WILLIAMS E D, BIRD D, FORBES A W, et al. Randomised controlled trial of an automated, interactive telephone intervention (TLC Diabetes) to improve type 2 diabetes management: Baseline findings and six-month outcomes [J]. BMC public health, 2012, 12(1).

[260] ZHAI Y, YU W. A mobile app for diabetes management: Impact on self-Efficacy among patients with type 2 diabetes at a community hospital [J]. Med Sci Monit, 2020, 26.

[261] ZHOU P, XU L, LIU X, et al. Web-based telemedicine for management of type 2 diabetes through glucose uploads: a randomized controlled trial [J]. International journal of clinical and experimental pathology, 2014, 7(12): 8848-54.

[262] BENHAMOU P Y. Improving diabetes management with electronic health records and patients' health records [J]. Diabetes Metab, 2011, 37(SUPPL. 4): S53-S6.

**3. Conference abstract, review article, protocol, letter and guidance [263-276]**

[263] CASTEN R, ROVNER B, CHANG A M, et al. A randomized clinical trial of a collaborative home-based diabetes intervention to reduce emergency department visits and hospitalizations in black individuals with diabetes [J]. Contemporary clinical trials, 2020, 95: 106069.

[264] EGEDE L E, KNAPP R G, WALKER R J, et al. Randomized controlled trial of technology-intensified diabetes education study (TIDES) in African Americans with type 2 diabetes [J]. Diabetes, 2020, 69.

[265] HEUDEBERT A, EICHOLD B, ARRIETA M I, et al. Real-time tele-monitoring of glucose as adjunct to the management of type 2 diabetes in primary care [J]. Journal of Investigative Medicine, 2013, 61(2): 398.

[266] LAICHUTHAI N, SAETANG T, GREEVIROJ P, et al. The efficacy of telemonitoring and integrated personalised diabetes management in people with insulin-treated type 2 diabetes: a preliminary analysis [J]. Diabetologia, 2023, 66: S433.

[267] LEE E Y, YUN J S, CHA S A, et al. Personalized Type 2 Diabetes Management Using a Mobile Application Integrated with Electronic Medical Records: An Ongoing Randomized Controlled Trial [J]. International journal of environmental research and public health, 2021, 18(10).

[268] NOSRAT S, MYERS V, NYKAZA E, et al. IDF2022-0576 The Impact of Remote Patient Monitoring on Glycated Hemoglobin for Type 2 Diabetes: A Randomized Controlled Trial [J]. Diabetes research and clinical practice, 2023, 197.

[269] OLEAGA A, GOñI F, PASCUAL T, et al. Evaluation of the use of new communication technologies in patients with type 2 diabetes [J]. Diabetologia, 2016, 59(1): S422.

[270] OWOLABI E O, TER GOON D, AJAYI A I. High Level of Acceptability and Feasibility with Low Level of Efficacy of Daily Text-Messaging on Glycaemic Status and Self-Management: Result of a Randomised Trial Among Low-Income Earning Black South Africans [J]. Metabolism: Clinical and Experimental, 2020, 104.

[271] PARSONS S N, LUZIO S D, OWENS D R. Using structured self-monitoring of blood glucose to improve diabetes knowledge: The SMBG study [J]. Diabetologia, 2018, 61: S434-S5.

[272] SHENG T, PARKS L, BABIKIAN S, et al. Glycemic improvements following mobile-enabled remote patient monitoring: A randomized control study [J]. Diabetes, 2020, 69.

[273] SOIN G, KUNYIHA N, SHAH J, et al. A randomized trial using mobile short-text messaging to improve cardiovascular risk profile in poorly controlled diabetes in kenya [J]. Circulation, 2018, 138.

[274] TAO H, SUN X Q, HE J H, et al. An interactive telemedicine system improves diabetes management for type 2 diabetic patients in china [J]. Diabetes, 2014, 63: A619.

[275] ISLAM S M S. Effectiveness and cost effectiveness of a mobile phone text messaging intervention for prevention of cardiovascular risk factors among patients with type 2 diabetes: A randomized controlled trial [J]. Journal of Hypertension, 2018, 36: e315.

[276] ZAMANILLO-CAMPOS R, FIOL-DEROQUE M A, SERRANO-RIPOLL M J, et al. Effectiveness of DiabeText, a mHealth intervention to support diabetes self-management: randomized controlled trial in primary care [Z]. 2024.10.1101/2024.02.28.24303489

**4. Not type 2 diabetes mellitus or have other co-morbidities [277-311]**

[277] ATTIA J R, HOLLIDAY E, WEAVER N, et al. The effect of zinc supplementation on glucose homeostasis: a randomised double-blind placebo-controlled trial [J]. Acta diabetologica, 2022, 59(7): 965-75.

[278] COHEN L B, TAVEIRA T H, WU W C, et al. Pharmacist-led telehealth disease management program for patients with diabetes and depression [J]. Journal of telemedicine and telecare, 2020, 26(5): 294-302.

[279] DI MOLFETTA S, PATRUNO P, CORMIO S, et al. A telemedicine-based approach with real-time transmission of blood glucose data improves metabolic control in insulin-treated diabetes: the DIAMONDS randomized clinical trial [J]. Journal of endocrinological investigation, 2022, 45(9): 1663-71.

[280] DOBSON R, WHITTAKER R, JIANG Y, et al. Long‐term follow‐up of a randomized controlled trial of a text‐message diabetes self‐management support programme, SMS4BG [J]. Diabet Med, 2020, 37(2): 311-8.

[281] FOUNTOULAKIS S, PAPANASTASIOU L, GRYPARIS A, et al. Impact and duration effect of telemonitoring on HbA1c, BMI and cost in insulin-treated diabetes mellitus patients with inadequate glycemic control: A randomized controlled study [J]. Horm, 2015, 14(4): 632-43.

[282] FRIAS J, VIRDI N, RAJA P, et al. Effectiveness of Digital Medicines to Improve Clinical Outcomes in Patients with Uncontrolled Hypertension and Type 2 Diabetes: Prospective, Open-Label, Cluster-Randomized Pilot Clinical Trial [J]. Journal of medical Internet research, 2017, 19(7): e246.

[283] HESSELDAL L, CHRISTENSEN J R, OLSESEN T B, et al. Long term weight loss in a primary care-anchored eHealth lifestyle coaching programme in Denmark: A randomised controlled trial [J]. Diabetologia, 2022, 65: S116.

[284] IMERAJ A, OLESEN T B, LAURSEN D H, et al. Agreement Between Clinically Measured Weight and Self-reported Weight Among Patients With Type 2 Diabetes Through an mHealth Lifestyle Coaching Program in Denmark: Secondary Analysis of a Randomized Controlled Trial [J]. JMIR Form Res, 2022, 6(9).

[285] KATULA J A, DRESSLER E V, KITTEL C A, et al. Effects of a Digital Diabetes Prevention Program: An RCT [J]. American journal of preventive medicine, 2022, 62(4): 567-77.

[286] KHUNTI K, GRIFFIN S, BRENNAN A, et al. Behavioural interventions to promote physical activity in a multiethnic population at high risk of diabetes: PROPELS three-arm RCT [J]. Health Technol Assess, 2021, 25(77): i-189.

[287] MANNOUBI C, KAIRY D, MENEZES K V, et al. The Key Digital Tool Features of Complex Telehealth Interventions Used for Type 2 Diabetes Self-Management and Monitoring With Health Professional Involvement: Scoping Review [J]. Jmir Medical Informatics, 2024, 12.

[288] NAGENDRA H R, NAGARATHNA R, RAJESH S K, et al. Niyantrita Madhumeha Bharata 2017, methodology for a nationwide diabetes prevalence estimate: Part 1 [J]. International Journal of Yoga, 2019, 12(3): 179-92.

[289] NANDITHA A, THOMSON H, SUSAIRAJ P, et al. A pragmatic and scalable strategy using mobile technology to promote sustained lifestyle changes to prevent type 2 diabetes in India and the UK: a randomised controlled trial [J]. Diabetologia, 2020, 63(3): 486-96.

[290] OR C, TAO D. A 3-Month Randomized Controlled Pilot Trial of a Patient-Centered, Computer-Based Self-Monitoring System for the Care of Type 2 Diabetes Mellitus and Hypertension [J]. Journal of medical systems, 2016, 40(4): 1-13.

[291] OR C K, LIU K, SO M K P, et al. Improving Self-Care in Patients With Coexisting Type 2 Diabetes and Hypertension by Technological Surrogate Nursing: Randomized Controlled Trial [J]. Journal of medical Internet research, 2020, 22(3): e16769.

[292] PATEL M R, ZHANG G, HEISLER M, et al. A Randomized Controlled Trial to Improve Unmet Social Needs and Clinical Outcomes Among Adults with Diabetes [J]. Journal of general internal medicine, 2024.

[293] PRISCILLA S, NANDITHA A, SIMON M, et al. A pragmatic and scalable strategy using mobile technology to promote sustained lifestyle changes to prevent type 2 diabetes in India-Outcome of screening [J]. Diabetes research and clinical practice, 2015, 110(3): 335-40.

[294] ROSAS L G, LV N, XIAO L, et al. Evaluation of a culturally-adapted lifestyle intervention to treat elevated cardiometabolic risk of Latino adults in primary care (Vida Sana): A randomized controlled trial [J]. Contemporary clinical trials, 2016, 48: 30-40.

[295] SEPAH S C, JIANG L, PETERS A L. Translating the Diabetes Prevention Program into an Online Social Network: Validation against CDC Standards [J]. The Diabetes educator, 2014, 40(4): 435-43.

[296] YE H, LIN L, ZHONG D, et al. The impact of telehealth education on self-management in patients with coexisting type 2 diabetes mellitus and hypertension: a 26-week randomized controlled trial [J]. Journal of endocrinological investigation, 2024.

[297] BAE J H, PARK E H, LEE H K, et al. Enhancing Diabetes Care through a Mobile Application: A Randomized Clinical Trial on Integrating Physical and Mental Health among Disadvantaged Individuals [J]. Diabetes Metab J, 2024, 48(4): 790-801.

[298] BELL A M, FONDA S J, WALKER M S, et al. Mobile phone-based video messages for diabetes self-care support [J]. Journal of diabetes science and technology, 2012, 6(2): 310-9.

[299] BLOCK G, AZAR K M J, ROMANELLI R J, et al. Diabetes prevention and weight loss with a fully automated behavioral intervention by email, web, and mobile phone: A randomized controlled trial among persons with prediabetes [J]. Journal of medical Internet research, 2015, 17(10).

[300] CHAN J C N, THEWJITCHAROEN Y, NGUYEN T K, et al. Effect of a Web-Based Management Guide on Risk Factors in Patients with Type 2 Diabetes and Diabetic Kidney Disease: A JADE Randomized Clinical Trial [J]. JAMA network open, 2022, 5(3): E223862.

[301] CHEUNG N W, REDFERN J, THIAGALINGAM A, et al. Effect of Mobile Phone Text Messaging Self-Management Support for Patients With Diabetes or Coronary Heart Disease in a Chronic Disease Management Program (SupportMe) on Blood Pressure: Pragmatic Randomized Controlled Trial [J]. Journal of medical Internet research, 2023, 25.

[302] FRANC S, HANAIRE H, BENHAMOU P Y, et al. DIABEO System Combining a Mobile App Software With and Without Telemonitoring Versus Standard Care: A Randomized Controlled Trial in Diabetes Patients Poorly Controlled with a Basal-Bolus Insulin Regimen [J]. Diabetes technology & therapeutics, 2020, 22(12): 904-11.

[303] HANSEL B, GIRAL P, GAMBOTTI L, et al. A Fully Automated Web-Based Program Improves Lifestyle Habits and HbA1c in Patients With Type 2 Diabetes and Abdominal Obesity: Randomized Trial of Patient E-Coaching Nutritional Support (The ANODE Study) [J]. Journal of medical Internet research, 2017, 19(11): e360.

[304] HESSELDAL L, CHRISTENSEN J R, OLESEN T B, et al. Long-term Weight Loss in a Primary Care-Anchored eHealth Lifestyle Coaching Program: Randomized Controlled Trial [J]. Journal of medical Internet research, 2022, 24(9): e39741.

[305] MCLEOD M, STANLEY J, SIGNAL V, et al. Impact of a comprehensive digital health programme on HbA1c and weight after 12 months for people with diabetes and prediabetes: a randomised controlled trial [J]. Diabetologia, 2020, 63(12): 2559-70.

[306] MOLAVYNEJAD S, MILADINIA M, JAHANGIRI M. A randomized trial of comparing video telecare education vs. in-person education on dietary regimen compliance in patients with type 2 diabetes mellitus: a support for clinical telehealth Providers [J]. BMC endocrine disorders, 2022, 22(1): 116.

[307] MORAVCOVá K, SOVOVá M, OŽANA J, et al. Comparing the Efficacy of Digital and In-Person Weight Loss Interventions for Patients with Obesity and Glycemic Disorders: Evidence from a Randomized Non-Inferiority Trial [J]. Nutrients, 2024, 16(10).

[308] TORO-RAMOS T, MICHAELIDES A, ANTON M, et al. Mobile Delivery of the Diabetes Prevention Program in People With Prediabetes: Randomized Controlled Trial [J]. JMIR mHealth and uHealth, 2020, 8(7): e17842.

[309] WAKEFIELD B J, KOOPMAN R J, KEPLINGER L E, et al. Effect of home telemonitoring on glycemic and blood pressure control in primary care clinic patients with diabetes [J]. Telemedicine journal and e-health : the official journal of the American Telemedicine Association, 2014, 20(3): 199-205.

[310] WHITEHOUSE C R, KNOWLES M, LONG J A, et al. Digital Health and Community Health Worker Support for Diabetes Management: a Randomized Controlled Trial [J]. Journal of general internal medicine, 2023, 38(1): 131-7.

[311] YOO H J, PARK M S, KIM T N, et al. A Ubiquitous Chronic Disease Care system using cellular phones and the internet [J]. Diabet Med, 2009, 26(6): 628-35.

**5. Not RCT (pilot studies also excluded) [312-324]**

[312] ALRIGE M, ALHARBEY R, CHATTERJEE S. The Effect of a Customized Nutrient-Profiling Approach on the Glycated Hemoglobin Levels of Patients with Type 2 Diabetes: Quasi-Experimental Study [J]. Journal of medical Internet research, 2020, 22(5).

[313] CALLAN J A, SEREIKA S M, CUI R F, et al. Cognitive Behavioral Therapy (CBT) Telehealth Augmented With a CBT Smartphone Application to Address Type 2 Diabetes Self-Management: A Randomized Pilot Trial [J]. Sci Diabetes Self Manag Care, 2022, 48(6): 492-504.

[314] FARIDI Z, LIBERTI L, SHUVAL K, et al. Evaluating the impact of mobile telephone technology on type 2 diabetic patients' self-management: The NICHE pilot study [J]. J Eval Clin Pract, 2008, 14(3): 465-9.

[315] HU L, SHI Y, WYLIE-ROSET J, et al. Feasibility of a family-oriented mHealth intervention for Chinese Americans with type 2 diabetes: A pilot randomized control trial [J]. PloS one, 2024, 19(3 March).

[316] JAFAR N, HURIYATI E, HARYANI, et al. Enhancing knowledge of Diabetes self-management and quality of life in people with Diabetes Mellitus by using Guru Diabetes Apps-based health coaching [J]. J Public Health Res, 2023, 12(3).

[317] MYERS A, PRESSWALA L, BISSOONAUTH A, et al. Telemedicine for Disparity Patients With Diabetes: The Feasibility of Utilizing Telehealth in the Management of Uncontrolled Type 2 Diabetes in Black and Hispanic Disparity Patients; A Pilot Study [J]. Journal of diabetes science and technology, 2020, 15(5): 1034-41.

[318] NAGREBETSKY A, LARSEN M, CRAVEN A, et al. Stepwise self-titration of oral glucose-lowering medication using a mobile telephone-based telehealth platform in type 2 diabetes: A feasibility trial in primary care [J]. Journal of diabetes science and technology, 2013, 7(1): 123-34.

[319] OH S W, KIM K K, KIM S S, et al. Effect of an Integrative Mobile Health Intervention in Patients With Hypertension and Diabetes: Crossover Study [J]. JMIR mHealth and uHealth, 2022, 10(1): e27192.

[320] ONYIA A U, BERHIE G, CECCHETTI A, et al. The Use of Digital Telehealth for the Self-Management of Type 2 Diabetes Patients in Hinds County, Mississippi: A Pilot Study [J]. Journal of Patient Experience, 2023, 10.

[321] SANI M, MAKEEN A, ALBASHEER O B A, et al. Effect of telemedicine messages integrated with peer group support on glycemic control in type 2 diabetics, Kingdom of Saudi Arabia [J]. Int J Diabetes Dev Countries, 2018, 38(4): 495-501.

[322] SHUKLA A P, KARAN A, GRAVES M, et al. A Randomized Controlled Pilot Study Of The Food Order Behavioral Intervention In Prediabetes [J]. Journal of the Endocrine Society, 2022, 6: A293.

[323] VAUGHAN E M, NAIK A D, AMSPOKER A B, et al. Mentored implementation to initiate a diabetes program in an underserved community: a pilot study [J]. BMJ open diabetes research & care, 2021, 9(1).

[324] WHITTEMORE R, VILAR-COMPTE M, DE LA CERDA S, et al. ¡sí, Yo Puedo Vivir Sano con Diabetes! A Self-Management Randomized Controlled Pilot Trial for Low-Income Adults with Type 2 Diabetes in Mexico City [J]. Curr Dev Nutr, 2020, 4(5).

**6. Not in English [325-328]**

[325] KIM K A, HWANG S Y. Effects of a daily life-based physical activity enhancement program for middle-aged women at risk for cardiovascular disease [J]. J Korean Acad Nurs, 2019, 49(2): 113-25.

[326] LANGE I, CAMPOS S, URRUTIA M, et al. Effect of a tele-care model on self-management and metabolic control among patients with type 2 diabetes in primary care centers in Santiago, Chile [J]. Rev Med Chile, 2010, 138(6): 729-37.

[327] PARK G, LEE H, KHANG A R. The Development of Automated Personalized Self-Care (APSC) Program for Patients with Type 2 Diabetes Mellitus [J]. J Korean Acad Nurs, 2022, 52(5): 535-49.

[328] SUKCHAISON N, PICHAYAPINYO P, LAGAMPAN S, et al. Effectiveness of the Mindfulness-Based Diabetes Self- and Family Management Support Program among Adults with Uncontrolled Diabetes: A Randomized Controlled Trial [J]. Pacific Rim Intl J Nurs Res, 2022, 26(3): 517-32.

# Section 4: Supplementary tables

**Table S1.** GRADE summary of findings: telemedicine vs. usual care.

| **Certainty assessment** | | | | | | | **№ of patients** | | **Effect** | | **Certainty** | **Importance** |
| --- | --- | --- | --- | --- | --- | --- | --- | --- | --- | --- | --- | --- |
| **№ of studies** | **Study design** | **Risk of bias** | **Inconsistency** | **Indirectness** | **Imprecision** | **Other considerations** | **Telemedicine** | **Usual care** | **Relative (95% CI)** | **Absolute (95% CI)** |  |  |
| **△HbA1c** | | | | | | | | | | | | |
| 51 | randomised trials | serious | serious | not serious | not serious | none | 5131 | 4842 | - | MD **0.38 lower** (0.49 lower to 0.27 lower) | ⨁⨁◯◯ Low | CRITICAL |
| **△FBG** | | | | | | | | | | | | |
| 16 | randomised trials | serious | serious | not serious | not serious | none | 1436 | 1359 | - | MD **11.29 mg/dL lower** (17.65 lower to 4.93 lower) | ⨁⨁◯◯ Low | IMPORTANT |
| **△weight** | | | | | | | | | | | | |
| 19 | randomised trials | serious | serious | not serious | not serious | none | 1955 | 1756 | - | MD **1.33 kg lower** (2.23 lower to 0.44 lower) | ⨁⨁◯◯ Low | IMPORTANT |
| **△BMI** | | | | | | | | | | | | |
| 22 | randomised trials | serious | serious | not serious | not serious | none | 2621 | 2419 | - | MD **0.43 lower** (0.72 lower to 0.13 lower) | ⨁⨁◯◯ Low | IMPORTANT |
| **△SBP** | | | | | | | | | | | | |
| 20 | randomised trials | serious | not serious | not serious | not serious | none | 2448 | 2280 | - | MD **2.14 mmHg lower** (3.02 lower to 1.26 lower) | ⨁⨁⨁◯ Moderate | IMPORTANT |
| **△DBP** | | | | | | | | | | | | |
| 19 | randomised trials | serious | not serious | not serious | not serious | none | 1890 | 1720 | - | MD **1.24 mmHg lower** (2.02 lower to 0.46 lower) | ⨁⨁⨁◯ Moderate | IMPORTANT |
| **△LDL-c** | | | | | | | | | | | | |
| 8 | randomised trials | serious | serious | not serious | not serious | none | 765 | 752 | - | MD **0.69 mg/dL lower** (11.69 lower to 10.31 higher) | ⨁⨁◯◯ Low | IMPORTANT |
| **△HDL-c** | | | | | | | | | | | | |
| 7 | randomised trials | serious | serious | not serious | not serious | none | 681 | 632 | - | MD **3.41 mg/dL lower** (2.67 lower to 9.49 higher) | ⨁⨁◯◯ Low | IMPORTANT |

**Table S2.** Summary of descriptive characteristics of included articles.

| Author, year | Country | Setting | Sample size I^a^/C^b^ (n) | Gender (proportion of male, %) | Age (mean, SD/ median, IQR/ age group, n, %/ range) | Intervention | Type of telemedicine | Intervention duration | Control | Outcomes |
| --- | --- | --- | --- | --- | --- | --- | --- | --- | --- | --- |
| Anzaldo-Campos et al (2016) [1] | Mexico | Family Medical Unit | 102/100 | I: 38.2% C: 38% | NR^c^ | Project Dulce technology enhanced with mobile tools (PD-TE) | Remote monitoring | 10 months | Standard care outlined by Mexican Institute of Social Security (IMSS) guidelines via recently introduced DiabetIMSS group medical visits or one-on-one visits with a family physician. | HbA1c^d^; BMI^e^; SBP^f^; DBP^g^; LDL-c^h^; HDL-c^i^ |
| Arora et al (2014) [2] | USA | Hospital | 64/64 | I: 40% C: 31% | I: 50.5±10.3 C: 51.0±10.2 | Text message–based mobile health intervention (TExT-MED) | Asynchronous | 6 months | Usual care | HbA1c |
| Azelton et al (2021) [3] | USA | Family Medical Unit | 16/14 | I: 40% C: 60% | NR | Healthy at Home, a 12-week phone and SMS-based (short message service) digital health coaching program | Remote monitoring | 12 weeks | Received care at the family medicine residency clinic which included a physicians’ office visit at the beginning and end of the study period (every 12 weeks). | HbA1c; Weight; SBP; DBP |
| Basudev et al (2016) [4] | UK | NR | 93/115 | I: 54.8% C: 59.6% | I: 60.5±12.3 C: 59.3±12.0 | Diabetes virtual clinic | NR | 12 months | Usual diabetes care according to local and national diabetes pathways | HbA1c; BMI; SBP; DBP |
| Bentley et al (2016) [5] | UK | NR | 9/9 | I: 77.8% C: 0 | I: 50.9±8.1 C: 55.7±9.9 | An mHealth supported weight loss intervention (AiperMotion 500) | Remote monitoring | 12 weeks | Advice on diet and exercise | HbA1c; Weight |
| Capozza et al (2015) [6] | USA | Primary care | 58/35 | I: 40% C: 31% | I: 52±11.2 C: 54.5±10.7 | Text-message program (Care4Life) | Asynchronous | 6 months | Usual care | HbA1c |
| Cho et al (2017) [7] | South Korea | Hospital | 244/240 | I: 63.5% C: 63.3% | I: 52.9±9.2 C: 53.4±8.7 | A health gateway with Internet-based communication, to which a glucose meter and electronic manometer could be mechanically linked and data automatically transferred | Remote monitoring | 24 weeks | Conventional outpatient management | HbA1c; Weight; FBG^j^; BMI; SBP; DBP; LDL-c; HDL-c |
| Christensen et al (2022) [8] | Denmark | Hospital | 81/55 | I: 37.0% C: 41.8% | I: 53.9±9.2 C: 53.0±11.6 | A telehealth lifestyle-coaching program (Liva) | Asynchronous | 24 months | Usual care | HbA1c; Weight |
| Christensen et al (2022) [9] | Denmark | Primary care | 100/70 | I: 51% C: 54.29% | I: 56.12±7.32 C: 57.07±9.94 | A telehealth lifestyle-coaching program (Liva) | Synchronous and asynchronous | 6 months | Standard care in Denmark: Quarterly visits to the general practitioners | HbA1c; Weight; BMI; SBP; DBP |
| Dale et al (2009) [10] | UK | Primary care | 44/97 | I: 52.4% C: 64.0% | Age group: I: Under 50 years-old: 14.3% 51–69 years-old: 59.5% Over 70 years-old: 26.2% C: Under 50 years-old: 13.5% 51–69 years-old: 60.7% Over 70 years-old: 25.8% | General practices | Synchronous | 6 months | Encouraged to follow the advice of their general practitioner or practice nurse. | HbA1c |
| Dario et al (2017) [11] | Italy | Local Health Authority (LHA) | 208/91 | I: 57.0% C: 53.0% | I: 73.05±5.79 C: 73.04±5.28 | HEALTH (RENEWING HEALTH) project, called Model for Assessment of Telemedicine (MAST) | Remote monitoring | 12 months | UC group had access to usual care and shared the paper log books of glucometer tests with their general practitioner or specialist during planned visits. | HbA1c |
| Dunkel et al (2023) [12] | Germany | NR | 86/65 | I: 80.2% C: 83.1% | I: 59.66±6.24 C: 58.80±7.33 | Programme initiative.diabetes | Synchronous and remote monitoring | 12 months | Diabetes care as usual | HbA1c; BMI |
| Eakin et al (2014) [13] | Australia | Primary care | 151/151 | I: 55.6% C: 57.0% | I: 57.7±8.1 C: 58.3±9.0 | TelephoneDelivered Weight Loss and Physical Activity Intervention | Synchronous | 18 months | Usual-care participants were mailed a brief summary of their results following each assessment, as well as standard, diabetes self-management education brochures. | HbA1c; Weight; SBP; DBP; LDL-c; HDL-c |
| Farmer et al (2021) [14] | Southern Africa | Primary care | 558/561 | I: 30.1% C: 30.1% | I: 56.8±11.6 C: 57.4±11.1 | SMS-Text Adherence Support for Type 2 Diabetes (StAR2D) trial | Asynchronous | 12 months | Usual care | HbA1c; BMI; SBP |
| Franc et al (2019) [15] | France | Hospital | C-G1^k^:62 I-G2: 64 I-G3: 63 | G1: 61.3% G2: 64.1% G3: 68.3% | G1: 59.6±9.3 G2: 58.1±10.3 G3: 58.4±9.2 | G2: Interactive voice response system (IVRS) | Synchronous | 13 months | Patients receiving the standard procedure for insulin initiation; Face-to-face visit in the fourth month (approximately 30 minutes) | HbA1c |
|  |  |  |  |  |  | G3: Diabeo-BI app software | Synchronous |  |  |  |
| Gerber et al (2023) [16] | USA | Primary care | 109/112 | I: 29.4% C: 31.3% | I: 56.0±9.3 C: 54.5±9.6 | Mobile health (mHealth) tools | Synchronous | 12 months | Participants received routine health care from their patients’ primary care physicians. | HbA1c |
| Gong et al (2020) [18] | Australia | NR | 93/94 | I: 47.3% C: 36.2% | I: 55.4±9.7 C: 58.4±10.5 | My Diabetes Coach (MDC) program, an app-based interactive embodied conversational agent | Synchronous and remote monitoring | 12 months | Participants in the control arm were encouraged to continue their routine diabetes self-care, including access to health care services, resources accessed via NDSS, and the diabetes not-for-profit organizations in their states. | HbA1c |
| Greenwood et al (2015) [19] | USA | Primary care | 45/45 | I: 75% C: 79% | I: 53.9±10.4 C: 57.5±10.6 | Care Innovations Guide, a telehealth remote monitoring system | Remote monitoring | 6 months | Participants in usual care received diabetes education booklets and referral for formal diabetes education as needed. | HbA1c |
| Haghighinejad et al (2022) [20] | Iran | Hospital | I-G1: 50 I-G2: 50 C-G3:46 | G1: 32% G2: 40% G3: 50% | G1: 57.1±7.4 G2: 54.0±8.5 G3: 57.5±9.0 | G1: Distance education based on mobile technology | Asynchronous | 3 months | The control group also underwent routine care at the family physician clinic. | HbA1c; FBG |
|  |  |  |  |  |  | G2: Short message service (SMS) |  |  |  |  |
| Hee-Sung et al (2007) [21] | South Korea | Hospital | 12/15 | I: 50% C: 40% | I: 43.4±7.9 C: 46.2±9.1 | Short message service (SMS) and wire Internet | Synchronous | 12 weeks | As customary in outpatient clinic for diabetic patients | HbA1c; FBG |
| Hoda et al (2023) [22] | India | Hospital | 50/50 | I: 38% C: 46% | I:  30 – 40: 14% 40 – 50: 32% 50 – 60: 38% 60 – 70: 16% C: 30 – 40: 12% 40 – 50: 22% 50 – 60: 34% 60 – 70: 32% | mhealth | Synchronous and asynchronous | 3 months | Routine diabetes care | HbA1c |
| Holmen et al (2014) [23] | Norway | Hospital | I-G1: 50 I-G2: 50 C-G3:50 | G1: 33% G2: 50% G3: 40% | G1: 58.6±11.8 G2: 57.4±12.1 G3: 55.9±12.2 | G1: Few Touch Application Intervention | Remote monitoring | 12 months | All participants received usual care by their GP according to national guidelines. | HbA1c; Weight |
|  |  |  |  |  |  | G2: Few Touch Application with Health Counseling Intervention | Synchronous and remote monitoring |  |  |  |
| Hsu et al (2016) [24] | USA | Hospital | 20/20 | NR | I: 53.3 C: 53.8 | Cloud-based diabetes management program | Synchronous and asynchronous | 12 ±2 weeks | Standard face-to-face care and phone follow-up | HbA1c |
| Jantraporn et al (2019) [58] | Thailand | Primary care | 26/27 | I: 34.6% C: 33.3% | I: 52.8±5.7 C: 53.4±4.4 | Telemonitoring | Synchronous | 12 weeks | Routine care including drug therapy and health education provided by the nurses in the clinic | HbA1c |
| Jarab et al (2012) [25] | Jordan | Hospital | 85/86 | I: 64% C: 62% | I: 63.4±10.1 C: 65.3±9.2 | A pharmacist-led pharmaceutical care intervention program | Synchronous | 6 months | Patients receive the usual care provided by the medical and nursing staff, which included patient assessment, a 3- or 6-month review at which blood glucose and blood pressure were measured, advice on self-monitoring of blood glucose (SMBG), and nutrition counseling. | HbA1c; BMI; SBP; DBP; LDL-c; HDL-c |
| Jeong et al (2018) [26] | South Korea | Hospital | I-G1: 113 I-G2: 112 C-G3:113 | G1: 66.37% G2: 68.75% G3: 67.26% | G1: 53.65±9.10 G2: 52.46±8.48 G3: 53.16±9.06 | G1: Telemonitoring | Remote monitoring | 24 weeks | Standard diabetes self-care education | HbA1c; BMI; Weight; SBP; DBP; LDL-c; HDL-c |
|  |  |  |  |  |  | G2: Telemedicine | Synchronous and remote monitoring |  |  |  |
| Kempf et al (2017) [27] | Germany | Institute | 93/74 | I: 55% C: 53% | I: 59±9 C: 60±8 | Telemedical Lifestyle intervention Program (TeLiPro) | Synchronous and remote monitoring | 12 weeks | Quarterly visits with their attending physician for routine health care visits as defined by the Disease Management Programs [DMP] for Type 2 Diabetes in Germany | HbA1c; BMI; FBG; Weight; SBP; DBP; LDL-c; HDL-c |
| Kempf et al (2023) [28] | Germany | Institute | 192/275 | NR | NR | Tultimodal Telemedical Lifestyle Intervention Program (TeLIPro) | Synchronous and asynchronous | 12 months | Routine care, quarterly DMP visits with their attending physician | HbA1c; BMI; SBP; DBP |
| Khanna et al (2014) [29] | USA | Primary care | 38/37 | I: 50% C: 68% | I: 51±12 C: 53±12 | Automated telephone nutrition support (ATNS) counseling | Synchronous | 12 weeks | Usual care | HbA1c; BMI; SBP; DBP |
| Kitazawa et al (2024) [30] | Japan | Institute | 86/82 | I: 80.2% C: 80.4% | I: 49.19±8.75 C: 47.38±7.49 | Lifestyle Intervention with Smartphone App and Intermittently scanned continuous glucose monitoring (isCGM) | Remote monitoring | 12 weeks | The patient does not receive any intervention, only the information from the examination. | HbA1c; Mean glucose; BMI; weight; SBP; DBP |
| Kleinman et al (2016) [31] | India | NR | 45/45 | NR | 48.4±9.2 | Gather mHealth System | NR | 6 months | Usual care | HbA1c |
| Klingeman et al (2017) [32] | USA | Primary care | 30/30 | I: 50% C: 56.7% | I: 54.43±9.6 C: 54.3±9.8 | Remote clinical interventions | Synchronous and asynchronous | 1 year | Standard endocrinology clinics | HbA1c |
| Kooiman et al (2018) [33] | USA | Healthcare organizations | 40/32 | NR | I: 56.8±11.4 C: 55.8±11.4 | An online self-tracking program | Remote monitoring | 12 weeks | Visit a diabetes nurse and/or physician every 3 months | HbA1c; BMI; Weight |
| Lauffenburger et al (2019) [34] | USA | Health insurer | 700/700 | I: 65.4% C: 60.2% | I: 54.9±8.1 C: 54.6±8.4 | Behaviorally-tailored telephone intervention | Synchronous | 12 months | Usual care | HbA1c |
| Lee et al (2022) [35] | South Korea | Hospital | I-G1: 91 I-G2: 91 C-G3:87 | G1: 44% G2: 42% G3: 43% | G1: 51.3±13.1 G2: 53.6±11.7 G3: 52.6±12.1 | G1: Mobile diabetes self-care | Remote monitoring | 26 weeks | Received Usual care according to the standard care for patients with T2DM by the South Korean Diabetes Association | HbA1c; FBG; BMI; Weight; SBP; DBP; LDL-c |
|  |  |  |  |  |  | G2: Mobile diabetes self-care with personalized, bidirectional feedback from physicians | Synchronous and remote monitoring |  |  |  |
| Lee et al (2020) [36] | Malaysia | Primary care | 120/120 | I: 44.2% C: 45.8% | I: 56.1±9.2 C: 56.3±8.6 | Telemonitoring (TG) | Remote monitoring | 6 months | Usual care participants continued to receive care from their doctors as they had in the past and monitor their blood glucose as required using a glucometer. | HbA1c; FBG; SBP; DBP |
| Leong et al (2022) [37] | China | Hospital | 91/90 | I:73.6% C: 63.3% | I: 59.0±11.4 C: 58.1±11.9 | LINE Oriented Video Education—delivered through a social media app | Synchronous and asynchronous | 3 months | Patient consultations with physicians, access to nurses in the outpatient services, as well as medication consultations with pharmacists upon receiving prescriptions | HbA1c |
| Lim et al (2021) [38] | Singapore | Primary care | 99/105 | I: 66.7% C: 62.9% | I: 51.6±9.4 C: 50.8±10.0 | A Smartphone App | Asynchronous and remote monitoring | 6 months | Receive standard diabetes care from their usual health care professionals | HbA1c; FBG; BMI; weight; SBP; DBP; LDL-c; HDL-c |
| Liou et al (2014) [39] | China | Primary care | 54/41 | I: 56.9% C: 48.8% | I: 56.6±7.7 C: 57.0±7.5 | Shared care combined with telecare | Synchronous | 6 months | Received one diabetes education session conducted individually by a licensed practical nurse | HbA1c; BMI; SBP; DBP |
| Lorig et al (2008) [40] | Spain | Primary care | 219/198 | I: 42.9% C: 32.8% | I: 52.9±13.2 C: 52.8±13.4 | Peerled Spanish Diabetes Self-Management Program (SDSMP) | Synchronous | 6 months | Usual care | HbA1c |
| Luley et al (2011) [41] | Germany | Hospital | 35/35 | I: 43% C: 54% | I: 57±9 C: 58±7 | Telemonitoring plus a diet combination - the Active Body Control (ABC) Program | Remote monitoring | 6 months | Conventional low-fat diet and standard care according to recommendations issued by the Deutschen Diabetes-Gesellschaft | HbA1c; Weight; BMI; HDL-c |
| Gómez et al (2022) [17] | Colombia | Hospital | 41/45 | I: 55% C: 54.2% | I: 58.6±10.6 C: 60.5±12.8 | ClouDi is a telemedicine platform, a web version, a version for mobile devices and a desktop application. | Synchronous and asynchronous | 3 months | Standard care | HbA1c |
| Mitchell et al (2023) [42] | USA | Primary care | 158/151 | NR | I: 55.54±11.5 C: 55.20±9.6 | An Immersive TelemedicinePlatform | Synchronous | 6 months | In-person diabetes medical group visits | HbA1c |
| Orsama et al (2013) [43] | Finland | NR | 24/24 | I: 54% C: 54% | I: 62.3±6.5 C: 61.5±9.1 | Technology for monitoring and remote reporting of diabetes health-related parameters from home | Remote monitoring | 10 months | Diabetes education, annual checkups, and diabetes guidance and education given by a doctor or nurse during patient-initiated visits to their health center. | HbA1c |
| Oseran, Rao et al (2022) [44] | USA | Primary care | 130/130 | I: 53.1% C: 55.4% | I: 62.6±11.6 C: 61.3±11.0 | Electronic consultation | Synchronous | 6 months | Standard care | HbA1c |
| Parsons et al (2019) [45] | UK | Hospital | 148/151 | I: 60% C: 58% | I: 61.6±9.82 C: 60.7±10.98 | ‘TeleCare’ support | Synchronous | 12 months | Routine HbA1c results were used to facilitate glycaemic management by their general practitioner as per usual care. | HbA1c; BMI; weight |
| Quinn et al (2011) [46] | USA | Primary care | 62/56 | I: 50% C: 50% | I: 52.0±8.0 C: 53.2±8.4 | Coach primary care provider portal with decisionsupport (CPDS) | Remote monitoring | 12 months | Usual care | HbA1c; SBP; DBP |
| Sachmechi et al (2023) [47] | USA | Hospital | 39/39 | I: 55.3% C: 43.4% | I: 58.9±10.3 C: 64.5±13.6 | A remote patient monitoring system | Synchronous | 12 weeks | Office visits at the beginning and end of the trial (12-week visit), with the participants instructed to measure and log their BG levels daily | HbA1c |
| Sarayani et al (2018) [48] | Iran | Hospital | 50/50 | I: 54.9% C: 62.0% | I: 53.4±10.3 C: 56.7±11.5 | A telephone‐based intervention | Synchronous | 3 months | Usual care | HbA1c |
| Sun et al (2019) [49] | China | Hospital | 44/47 | I: 43% C: 38% | Median, IQR I: 67.9 (66-71) C: 68.04 (66-72) | mHealth management app | Synchronous and remote monitoring | 3 months | Conventional outpatient clinic appointments | HbA1c |
| Tang et al (2013) [50] | USA | Institute | 202/213 | I: 58.9% C: 61.0% | I: 54.0±10.7 C: 53.5±10.2 | Online disease management of diabetes | Asynchronous and remote monitoring | 12 months | Usual care | HbA1c |
| Torbjørnsen et al (2014) [51] | Norway | Study center | 50/50 | I: 50% C: 60% | I: 57.4±12.1 C: 55.9±12.2 | Few touch application with health counseling intervention | Synchronous and asynchronous | 4 months | Received usual care according to the Norwegian clinical guidelines | HbA1c |
| Turnin et al (2021) [52] | France | Hospital | 128/135 | I: 64.1% C: 62.2% | I: 59.8±9.2 C: 59.3±10.0 | A remote monitoring programme including lifestyle education software | Remote monitoring | 1 year | Routine follow-up | HbA1c |
| Vaughan et al (2021) [53] | USA | Clinic | 44/45 | I: 22.73% C: 33.3% | I: 55.99±7.12 C: 53.86±9.07 | TIME (Telehealth-supported, Integrated care with CHWs, and MEdication-access) | Synchronous | 6 months | Usual care in the clinic included diabetes management with physicians (quarterly) and clinical pharmacists (monthly) in addition to routinely offered nutrition classes. | HbA1c |
| Yin et al (2022) [54] | China | Clinic | 52/47 | I: 43% C: 38% | I: 47.5 (43.0-51.0) C: 47.0 (42.0-51.0) | Telemedicine app | Remote monitoring | 6 months | Received traditional health education, which included diet, exercise, and medication guidance, during clinic visits. | HbA1c; BMI; FBG; weight; SBP; DBP; LDL-c; HDL-c |
| Zhang et al (2024) [55] | China | Primary care | 1,038/1,034 | I: 43.3% C: 46.5% | I: 61.4±7.1 C: 61.6±6.9 | SMARTDiabetes platform | Remote monitoring | 24 months | Usual care | HbA1c; FBG; SBP; DBP; LDL-c |
| Yang et al (2022) [56] | China | Primary care | 50/50 | I: 38% C: 42% | I: 66.9±6.06 C: 67.34±5.33 | Mobile phone–based telemedicine management | Synchronous and asynchronous | 12 months | Patients in the control group came to see the outpatient doctor per standard of care during the study period and received simple dietary advise or exercise recommendation each visit. | HbA1c; FBG; BMI; weight; SBP; DBP |
| Yang et al (2020) [57] | South Korea | Primary care | 150/97 | I: 53.3% C: 46.0% | I: 54.1±10.1 C: 60.6±10.2 | A mobile phone–based glucose-monitoring and feedback system | Asynchronous | 3 months | Visited the outpatient clinic and received face-to-face consultations for individual management target of risk factors | HbA1c; FBG; BMI; weight; SBP; DBP; LDL-c; HDL-c |
| Abbreviations: I^a^: Intervention group; C^b^: Control group; NR^c^: Not reported; HbA1c^d^: Glycated hemoglobin; BMI^e^: Body Mass Index; SBP^f^: Systolic blood pressure; DBP^g^: Diastolic blood pressure; LDL-c^h^: Low-density lipoprotein cholesterol; LDL-c^i^: High-density lipoprotein cholesterol; FBG^j^: Fasting blood glucose; G^k^: group. | | | | | | | | | | |

**Table S3.** Summary of telemedicine tools and intervention components by study.

| Author, year | Tools | Intervention components | | | | | | | | | | | |
| --- | --- | --- | --- | --- | --- | --- | --- | --- | --- | --- | --- | --- | --- |
|  |  | Consulting | | Treatment | | Education | | Coaching | Monitoring | Reminders and Notifications | Medication management | Supervision | Feedback |
| Anzaldo-Campos et al (2016) [1] | A MyGlucoHealth glucose meter; text; video |  |  | | ※ | |  | | ※ |  |  |  |  |
| Arora et al (2014) [2] | Text |  |  | | ※ | |  | |  | ※ |  |  |  |
| Azelton et al (2021) [3] | Telephone calls; text |  |  | |  | | ※ | | ※ | ※ |  |  |  |
| Basudev et al (2016) [4] | NR |  | ※ | |  | |  | |  |  |  |  |  |
| Bentley et al (2016) [5] | Monitoring device; email |  |  | |  | |  | | ※ | ※ |  |  |  |
| Capozza et al (2015) [6] | Text |  |  | | ※ | |  | |  | ※ |  |  |  |
| Cho et al (2017) [7] | Monitoring device |  |  | |  | |  | | ※ |  |  |  |  |
| Christensen et al (2022) [8] | Application |  |  | |  | | ※ | |  |  | ※ |  |  |
| Christensen et al (2022) [9] | Application |  |  | |  | | ※ | |  | ※ |  |  | ※ |
| Dale et al (2009) [10] | Telephone calls |  |  | |  | |  | |  |  |  | ※ |  |
| Dario et al (2017) [11] | Monitoring device |  |  | |  | |  | | ※ |  |  |  |  |
| Dunkel et al (2023) [12] | Monitoring device; telephone calls |  |  | |  | | ※ | | ※ |  |  |  | ※ |
| Eakin et al (2014) [13] | Telephone calls | ※ |  | |  | |  | |  |  |  | ※ |  |
| Farmer et al (2021) [14] | Text |  |  | | ※ | |  | |  | ※ |  |  |  |
| Franc et al (2019) [15] | Telephone calls | ※ |  | |  | |  | |  |  |  |  |  |
|  | Telephone calls; application | ※ | ※ | |  | |  | |  |  |  |  |  |
| Gerber et al (2023) [16] | Audio or video | ※ |  | | ※ | |  | |  |  |  |  |  |
| Gong et al (2020) [18] | Application; website; remote device | ※ |  | |  | | ※ | | ※ |  |  |  |  |
| Greenwood et al (2015) [19] | Remote device; video | ※ |  | | ※ | |  | |  |  |  |  | ※ |
| Haghighinejad et al (2022) [20] | Power-point presentations |  |  | | ※ | |  | |  |  |  |  |  |
|  | Text |  |  | | ※ | |  | |  |  |  |  |  |
| Hee-Sung et al (2007) [21] | Text |  | ※ | | ※ | |  | | ※ |  |  |  |  |
| Hoda et al (2023) [22] | Telephone calls; text | ※ |  | |  | |  | |  |  |  |  |  |
| Holmen et al (2014) [23] | Application; remote device |  |  | |  | |  | | ※ |  |  |  |  |
|  | Application; remote device; telephone calls | ※ |  | |  | |  | | ※ |  |  |  |  |
| Hsu et al (2016) [24] | Audio, video, and shared screen control | ※ |  | |  | |  | | ※ |  |  |  |  |
| Jantraporn et al (2019) [58] | Telephone calls |  |  | |  | |  | | ※ |  |  |  | ※ |
| Jarab et al (2012) [25] | Telephone calls | ※ |  | |  | |  | |  |  |  |  |  |
| Jeong et al (2018) [26] | Remote device; text |  | ※ | |  | |  | | ※ |  | ※ |  | ※ |
|  | Remote device; video conferencing | ※ | ※ | |  | |  | | ※ |  |  |  | ※ |
| Kempf et al (2017) [27] | Telephone calls; remote device |  |  | |  | | ※ | | ※ |  |  |  |  |
| Kempf et al (2023) [28] | Remote monitoring device; telephone calls |  |  | |  | | ※ | | ※ |  |  |  |  |
| Khanna et al (2014) [29] | Telephone calls | ※ |  | | ※ | |  | |  |  |  |  |  |
| Kitazawa et al (2024) [30] | Remote monitoring device; application |  |  | |  | |  | | ※ |  |  |  |  |
| Kleinman et al (2016) [31] | Application | ※ |  | |  | |  | |  |  |  |  |  |
| Klingeman et al (2017) [32] | Telephone calls; text |  |  | | ※ | |  | | ※ | ※ |  |  |  |
| Kooiman et al (2018) [33] | Application |  |  | | ※ | |  | |  |  |  | ※ |  |
| Lauffenburger et al (2019) [34] | Telephone calls | ※ |  | |  | |  | |  |  |  |  |  |
| Lee et al (2022) [35] | Application; text |  |  | | ※ | |  | | ※ |  |  |  |  |
|  | Application; text | ※ |  | | ※ | |  | | ※ |  |  |  |  |
| Lee et al (2020) [36] | Remote monitoring device; text | ※ |  | | ※ | |  | | ※ |  | ※ |  |  |
| Leong et al (2022) [37] | Application; video; audio or video call | ※ |  | | ※ | |  | |  |  |  |  |  |
| Lim et al (2021) [38] | Application; remote monitoring device; video | ※ |  | | ※ | |  | |  |  |  |  |  |
| Liou et al (2014) [39] | Videoconference | ※ |  | | ※ | |  | |  |  | ※ |  |  |
| Lorig et al (2008) [40] | Telephone calls | ※ |  | |  | |  | |  |  |  |  |  |
| Luley et al (2011) [41] | Remote monitoring device; email |  |  | |  | |  | | ※ |  |  |  | ※ |
| Orsama et AL (2013) [43] | Remote monitoring device; telephone; application | ※ |  | |  | |  | | ※ |  |  | ※ | ※ |
| Oseran, Rao et al (2022) [44] | NR | ※ |  | |  | |  | |  |  |  |  |  |
| Parsons et al (2019) [45] | Telephone calls | ※ |  | |  | |  | |  |  |  |  |  |
| Quinn et al (2011) [46] | Website |  |  | |  | | ※ | |  |  |  |  |  |
| Sachmechi et al (2023) [47] | Application; telephone calls | ※ |  | | ※ | |  | |  |  | ※ |  |  |
| Sarayani et al (2018) [48] | Telephone calls | ※ |  | |  | |  | |  |  |  |  |  |
| Sun et al (2019) [49] | Application; telephone calls |  |  | | ※ | |  | |  | ※ |  |  |  |
| Tang et al (2013) [50] | Remote monitoring device; text; video |  |  | | ※ | |  | | ※ |  |  |  |  |
| Torbjørnsen et al (2014) [51] | Application; remote monitoring device; telephone calls; text | ※ |  | |  | |  | | ※ |  |  |  |  |
| Turnin et al (2021) [52] | Remote monitoring device; application; website |  |  | | ※ | |  | | ※ |  |  |  |  |
| Vaughan et al (2021) [53] | Videoconference; telephone calls; text | ※ |  | |  | | ※ | |  |  |  |  |  |
| Yin et et al (2022) [54] | Application; remote monitoring device |  |  | | ※ | |  | | ※ |  |  |  |  |
| Zhang et al (2024) [55] | Application | ※ |  | | ※ | |  | | ※ |  | ※ |  |  |
| Yang et al (2022) [56] | Application; telephone calls |  |  | | ※ | |  | | ※ | ※ |  |  |  |
| Yang et al (2020) [57] | Application; text | ※ |  | |  | |  | | ※ |  |  |  | ※ |

**Table S4.** Risk-of-bias summary according to the Cochrane risk-of-bias tool for randomized trials.

| Author, year | Random sequence generation (selection bias) | Allocation concealment (selection bias) | Blinding of participants and personnel  (performance bias) | Blinding of outcome assessment (detection bias) | Incomplete outcome data (attrition bias) | Selective (reporting bias) | other |
| --- | --- | --- | --- | --- | --- | --- | --- |
| Anzaldo-Campos et al (2016) [1] | + | - | × | + | + | + | - |
| Arora et al (2014) [2] | - | - | × | + | + | + | × |
| Azelton et al (2021) [3] | + | - | × | + | + | + | × |
| Basudev et al (2016) [4] | + | + | + | + | + | + | × |
| Bentley et al (2016) [5] | + | + | + | + | + | + | - |
| Capozza et al (2015) [6] | + | - | × | + | + | + | × |
| Cho et al (2017) [7] | + | - | × | + | + | + | - |
| Christensen et al (2022) [8] | + | - | - | + | + | + | + |
| Christensen et al (2022) [9] | + | + | × | + | × | + | + |
| Dale et al (2009) [10] | + | + | - | + | + | + | - |
| Dario et al (2017) [11[ | + | + | - | + | × | + | - |
| Dunkel et al (2023) [12] | - | - | - | + | + | + | + |
| Eakin et al (2014) [13] | + | - | - | + | + | + | × |
| Farmer et al (2021) [14] | + | + | + | + | + | + | - |
| Franc et al (2019) [15] | - | - | × | + | + | + | × |
| Gerber et al (2023) [16] | + | - | × | + | + | + | × |
| Gómez et al (2022) [17] | + | + | × | + | + | + | + |
| Gong et al (2020) [18] | + | + | × | + | + | + | + |
| Greenwood et al (2015) [19] | + | + | × | + | - | + | × |
| Haghighinejad et al (2022) [20] | + | + | - | + | + | + | + |
| Hee-Sung et al (2007) [21] | + | + | + | + | + | + | - |
| Hoda et al (2023) [22] | + | + | × | + | + | + | + |
| Holmen et al (2014) [23] | + | - | × | + | + | + | + |
| Hsu et al (2016) [24] | + | - | - | + | + | + | + |
| Jantraporn et al (2019) [58] | - | - | - | + | + | + | - |
| Jarab et al (2012) [25] | + | - | - | + | + | + | + |
| Jeong et al (2018) [26] | - | - | - | + | + | + | - |
| Kempf et al (2017) [27] | + | + | + | + | + | + | - |
| Kempf et al (2023) [28] | + | + | × | + | + | + | + |
| Khanna et al (2014) [29] | + | - | - | + | + | + | - |
| Kitazawa et al (2024) [30] | + | + | × | + | + | + | + |
| Kleinman et al (2016) [31] | - | - | - | + | × | + | - |
| Klingeman et al (2017) [32] | - | - | × | + | + | + | × |
| Kooiman et al (2018) [33] | + | - | - | + | + | + | + |
| Lauffenburger et al (2019) [34] | + | - | + | + | + | + | × |
| Lee et al (2022) [35] | + | + | × | + | + | + | - |
| Lee et al (2020) [36] | + | + | × | + | + | + | × |
| Leong et al (2022) [37] | + | - | × | + | + | + | × |
| Lim et al (2021) [38] | + | × | × | + | + | + | × |
| Liou et al (2014) [39] | - | - | - | + | - | + | - |
| Lorig et al (2008) [40] | - | - | × | + | + | + | + |
| Luley et al (2011) [41] | - | - | - | + | + | + | - |
| Mitchell et al (2023) [42] | + | - | - | + | + | + | × |
| Orsama et al (2013) [43] | + | - | × | + | + | + | - |
| Oseran, Rao et al (2022) [44] | + | - | × | + | + | + | - |
| Parsons et al (2019) [45] | + | - | × | + | + | + | - |
| Quinn et al (2011) [46] | + | - | - | + | + | + | + |
| Sachmechi et al (2023) [47] | + | × | × | + | + | + | + |
| Sarayani et al (2018) [48] | + | + | × | + | + | + | × |
| Sun et al (2019) [49] | + | - | - | + | × | + | × |
| Tang et al (2013) [50] | - | - | - | + | + | + | + |
| Torbjørnsen et al (2014) [51] | + | - | × | + | + | + | + |
| Turnin et al (2021) [52] | + | - | × | + | + | + | × |
| Vaughan et al (2021) [53] | + | - | - | + | + | + | + |
| Yin et al (2022) [54] | + | - | - | + | + | + | × |
| Zhang et al (2024) [55] | + | × | × | + | + | + | + |
| Yang et al (2022) [56] | + | - | - | + | + | + | × |
| Yang et al (2020) [57] | + | - | × | + | + | + | × |
| “+”: Low risk of bias  “×”: High risk of bias  “-”: Unclear risk of bias | | | | | | | |

**Table S5.** Summary of effect sizes and heterogeneity for various outcomes in meta-analysis.

| Outcome | No. of Included Studies (Comparisons) | Effect Size (MD, 95% CI) | Heterogeneity (I², τ², p-value) | Significance (p-value) | Interpretation of Between-Group Results |
| --- | --- | --- | --- | --- | --- |
| FBG^a^ | 13 (16) | -11.29 [-17.65, -4.93] | I²: 99%; τ²: 137.24; p < 0.001 | p < 0.001 | Greater significant reduction, high heterogeneity |
| Weight | 18 (21) | -1.33 [-2.23, -0.44] | I²: 97%; τ²: 3.84; p < 0.001 | P=0.004 | Greater significant reduction, high heterogeneity |
| BMI^b^ | 20 (22) | -0.43 [-0.72, -0.13] | I²: 93%; τ²: 0.4; p < 0.001 | P=0.004 | Greater significant reduction, high heterogeneity |
| SBP^c^ | 20 (21) | -2.14 [-3.02, -1.26] | I²: 64%; τ²: 1.18; p < 0.001 | p < 0.001 | Greater significant reduction, substantial heterogeneity |
| DBP^d^ | 19 (20) | -1.24 [-2.02, -0.46] | I²: 50%; τ²: 1.07; p=0.006 | P=0.002 | Greater significant reduction, moderate heterogeneity |
| LDL-c^e^ | 8 (10) | -0.69 [-11.69, 10.31] | I²: 100%; τ²: 303.82; p < 0.001 | P=0.900 | No significant difference, high heterogeneity |
| HDL-c^f^ | 7 (8) | 3.41 [-2.67, 9.49] | I²: 99%; τ²: 75.68; p < 0.001 | P=0.270 | No significant difference, high heterogeneity |
| Abbreviations: FBGᵃ: fasting blood glucose; BMIᵇ: body mass index; SBPᶜ: systolic blood pressure; DBPᵈ: diastolic blood pressure; LDL-cᵉ: low-density lipoprotein cholesterol; HDL-cᶠ: high-density lipoprotein cholesterol. | | | | | |

# Section 5: Supplementary figures

A

B

C

**Figure S1.** A, Studies on telemedicine published by country; B, Studies on telemedicine published by continent; C, The number of articles published over the years illustrates the trend in article publication.


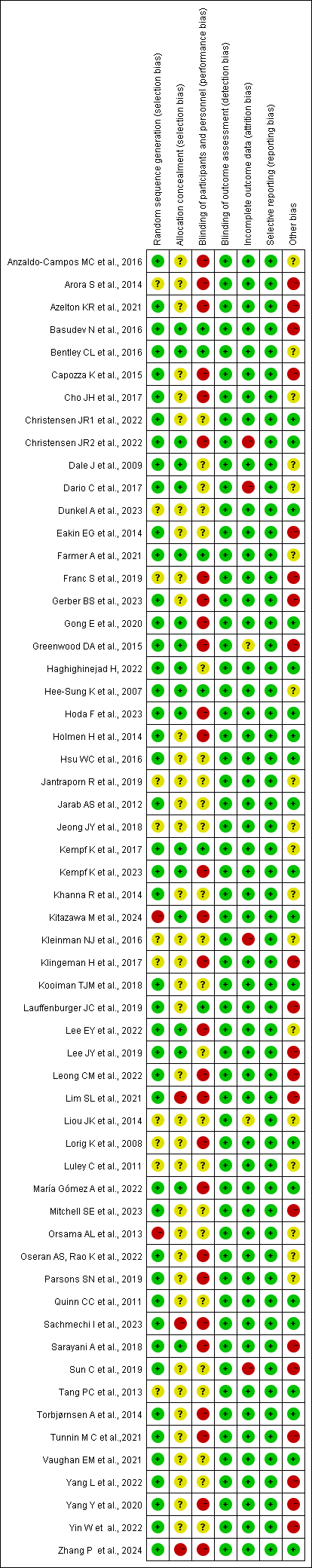


**Figure S2.** RoB assessment: traffic light plot. RoB: risk of bias (58 studies [1-58]).


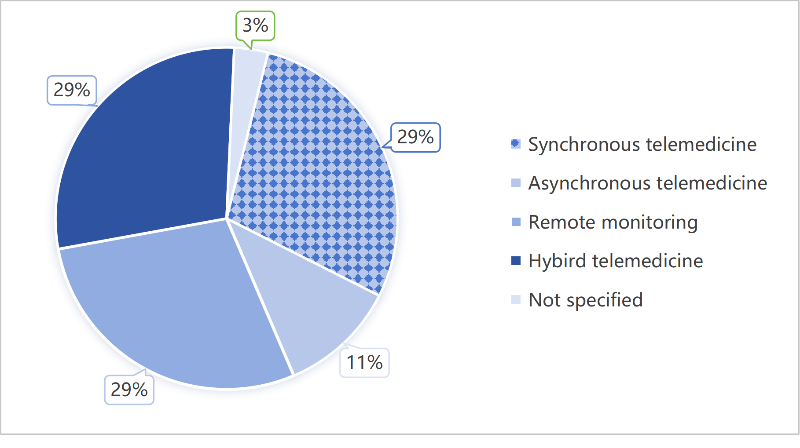


A


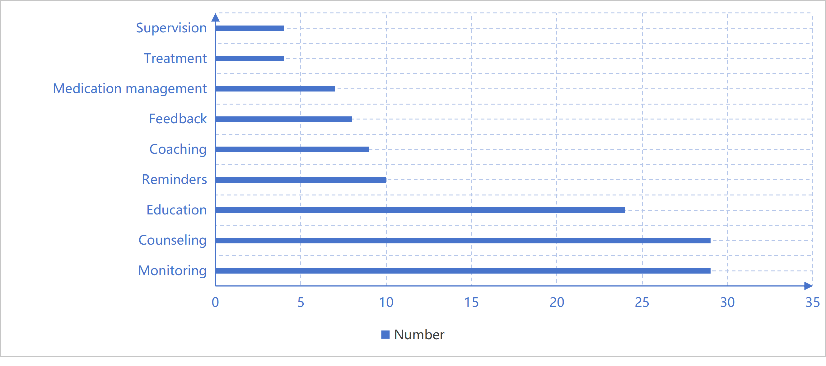


B


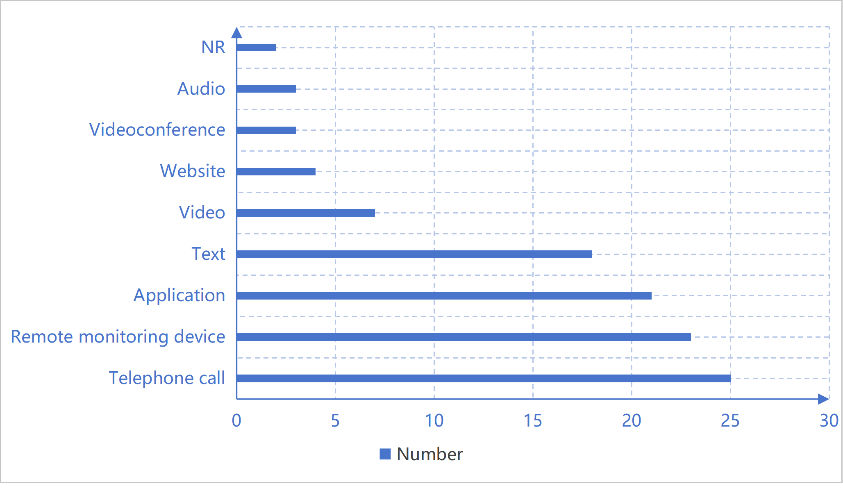


C

**Figure S3.** Overview of telemedicine classifications in type 2 diabetes management. (A) Classification according to the type of telemedicine. (B) Classification according to the aim of telemedicine. (C) Classification according to the tools of telemedicine.


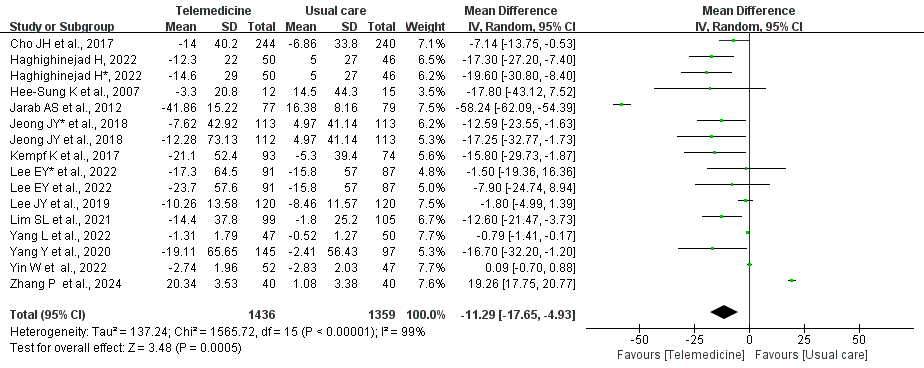

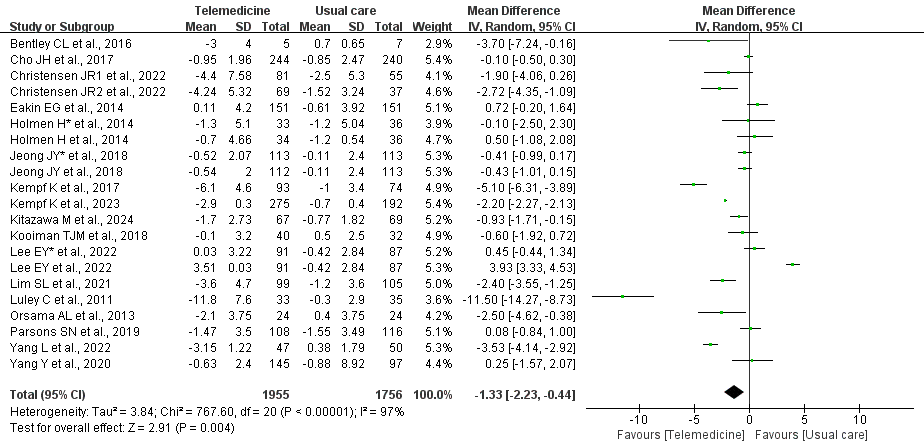


A B


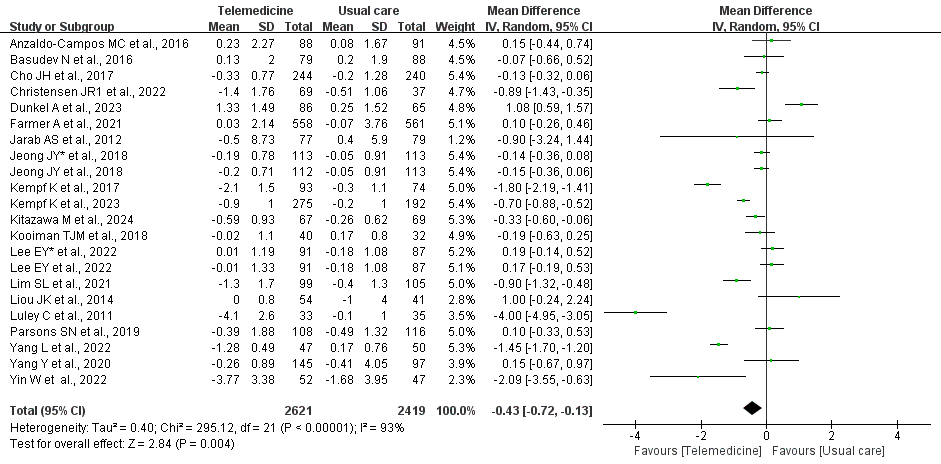

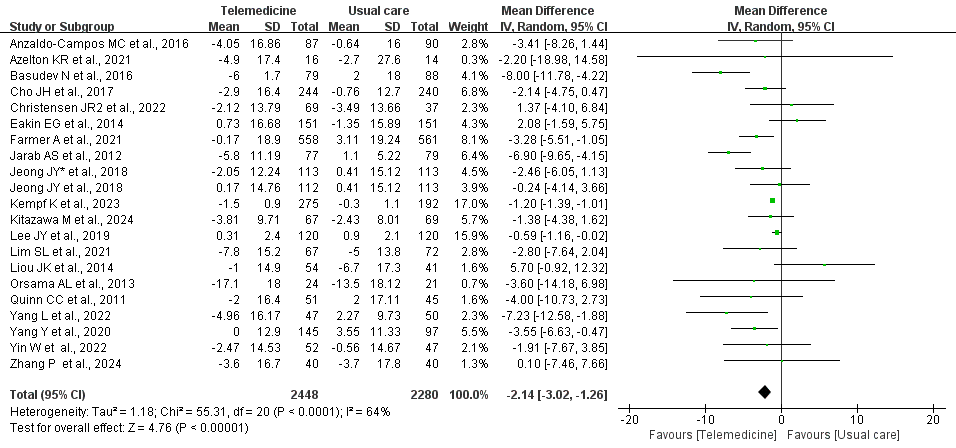


C D


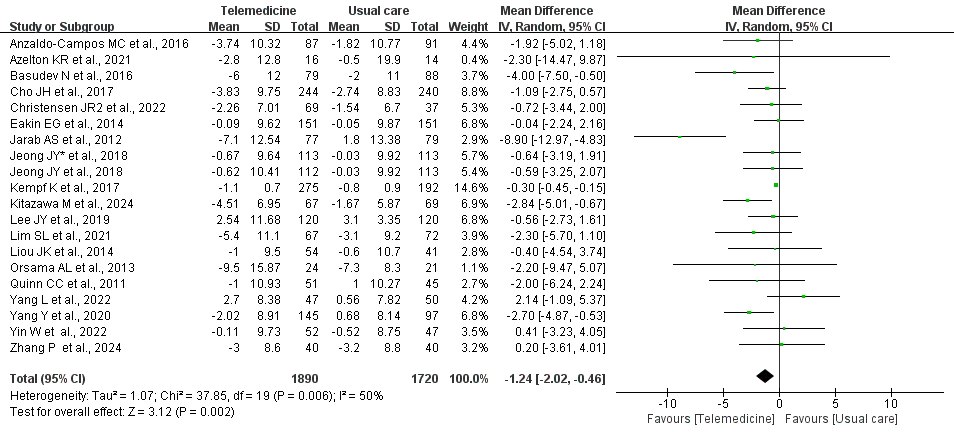

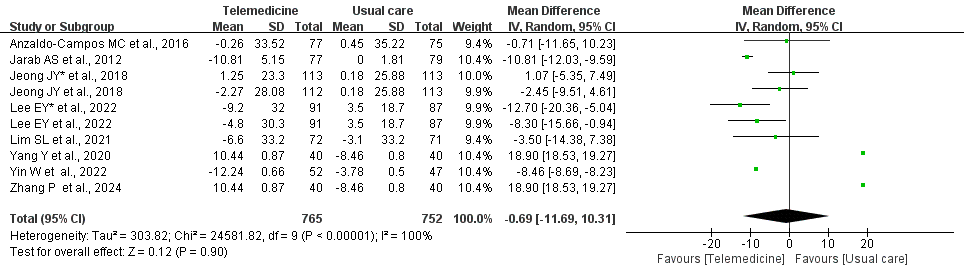


E F


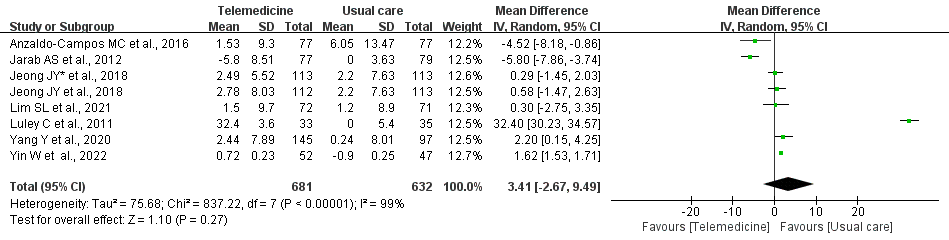


G

**Figure S4.** Forest plots: Effect of telemedicine interventions on clinical outcomes. A, Meta-analysis of △FBG between intervention and control group (13 studies [7,20-21,25-27,35,36,38,54-57); B, Meta-analysis of △weight between intervention and control group (19 studies [5,7-9,13,23,26-28,30,33,38,41,43,45,56,57); C. Meta-analysis of △BMI between intervention and control group (20 studies [1,4,7,9,12,14,25-28,30,33,38,39,41,45,54,56,57]); D, Meta-analysis of △SBP between intervention and control group (20 studies [1,3,4,7,9,13,14,25,26,28,30,36,38,39,43,46,54-57]); E, Meta-analysis of △DBP between intervention and control group (19 studies [1,3,4,7,9,13,25,26,28,30,36,38,39,43,46,54-57]); F, Meta-analysis of △LDL-c between intervention and control group (8 studies [1,25,26,35,38,54,55,57]); G: Meta-analysis of △HDL-c between intervention and control group (7 studies [1,25,26,38,54,57]). *: The study used two different telemedicine tools for the intervention, which are used here to differentiate; *: A study used two different telemedicine tools for the intervention, which are used here to differentiate.


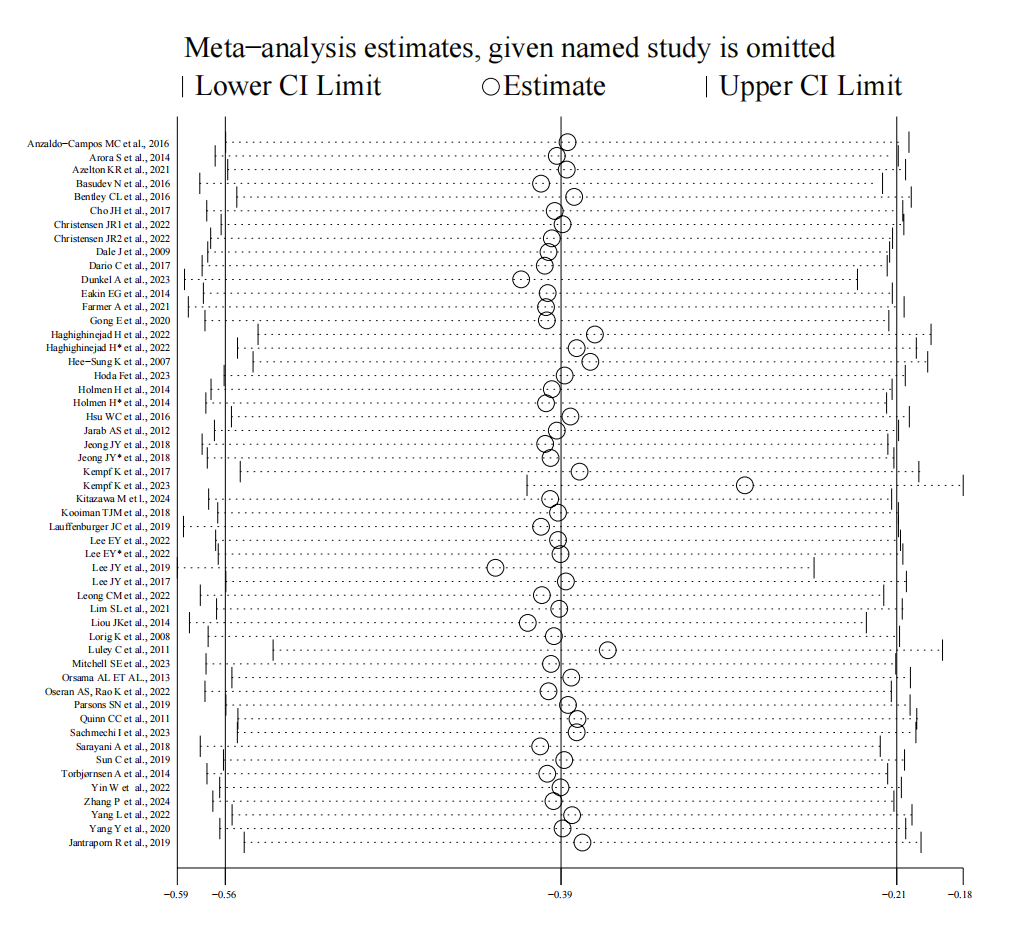


**Figure S5.** Sensitivity analysis for the impact of telemedicine on HbA1c levels. *: A study used two different telemedicine tools for the intervention, which are used here to differentiate (47 studies [1-5,7-14,18,20-28,30,33-49,51,54-58]).
